# Kinetic measurement system use in individuals following anterior cruciate ligament reconstruction: a scoping review of methodological approaches

**DOI:** 10.1186/s40634-021-00397-0

**Published:** 2021-09-26

**Authors:** Wasim Labban, Meredith Stadnyk, Mark Sommerfeldt, Stephanie Nathanail, Liz Dennett, Lindsey Westover, Thaer Manaseer, Lauren Beaupre

**Affiliations:** 1 Faculty of Rehabilitation Medicine, University of Alberta, Edmonton, Alberta Canada; 2 Mirdif Center for Physiotherapy and Rehabilitation, Dubai, United Arab Emirates; 3 Faculty of Medicine & Dentistry, University of Alberta, Edmonton, Alberta Canada; 4 Division of Orthopedic Surgery, Department of Surgery, Faculty of Medicine & Dentistry, University of Alberta, Edmonton, Alberta Canada; 5 Glen Sather Sports Medicine Clinic, University of Alberta, Edmonton, Alberta Canada; 6 Orthopedic Surgery, Alberta Health Services, Edmonton, Alberta Canada; 7 John W. Scott Health Sciences Library, University of Alberta, Edmonton, Alberta Canada; 8 Faculty of Engineering, University of Alberta, Edmonton, Alberta Canada

**Keywords:** Knee joint, Physical functional performance, Anterior cruciate ligament reconstruction, Athletes, Kinetics, Force plate, Data reporting, Return to sport

## Abstract

**Purpose:**

Our primary objectives were to (1) describe current approaches for kinetic measurements in individuals following anterior cruciate ligament reconstruction (ACLR) and (2) suggest considerations for methodological reporting. Secondarily, we explored the relationship between kinetic measurement system findings and patient-reported outcome measures (PROMs).

**Methods:**

We followed the PRISMA extension for scoping reviews and Arksey and O’Malley’s 6-stage framework. Seven electronic databases were systematically searched from inception to June 2020. Original research papers reporting parameters measured by kinetic measurement systems in individuals at least 6-months post primary ACLR were included.

**Results:**

In 158 included studies, 7 kinetic measurement systems (force plates, balance platforms, pressure mats, force-measuring treadmills, Wii balance boards, contact mats connected to jump systems, and single-sensor insoles) were identified 4 main movement categories (landing/jumping, standing balance, gait, and other functional tasks). Substantial heterogeneity was noted in the methods used and outcomes assessed; this review highlighted common methodological reporting gaps for essential items related to movement tasks, kinetic system features, justification and operationalization of selected outcome parameters, participant preparation, and testing protocol details. Accordingly, we suggest considerations for methodological reporting in future research. Only 6 studies included PROMs with inconsistency in the reported parameters and/or PROMs.

**Conclusion:**

Clear and accurate reporting is vital to facilitate cross-study comparisons and improve the clinical application of kinetic measurement systems after ACLR. Based on the current evidence, we suggest methodological considerations to guide reporting in future research. Future studies are needed to examine potential correlations between kinetic parameters and PROMs.

**Supplementary Information:**

The online version contains supplementary material available at 10.1186/s40634-021-00397-0.

## Introduction

The decision to return to sport (RTS) following anterior cruciate ligament reconstruction (ACLR) is a complex process [6]. Common criteria used to make this decision include: time from ACLR, functional performance, clinical examination findings, hop tests results, muscular strength, knee range of motion, neuromuscular control, and patient-reported outcome measures (PROMs) [37]. However, the validity of these criteria, when studied individually or combined, has been increasingly questioned [28, 42, 77, 106]. This mandates researchers and clinicians to incorporate objective and accurate biomechanical assessment systems to inform RTS decision-making post-ACLR [48].

Biomechanical assessment systems include kinetic and kinematic measurement systems. These systems can be synchronized with electromyography to examine muscular activity when required [162]. Kinetic and kinematic measurement systems are used to measure force (e.g., force plates) and joint angles (i.e., motion capture systems), respectively. Moreover, these two systems can be used together to measure important kinetic parameters such as joint moments. With the rapid advancement in the field of biomechanics, recent studies are examining the use of different motion capture systems to estimate kinetic parameters such as ground reaction forces and joint moments [74, 75, 132]. However, the methodologies used to estimate joint moments are debatable [29] and kinetic measurement systems are still considered the gold standard when measuring forces [108].

Different kinetic measurement systems have emerged as instruments to objectively assess various functions such as jumping [5, 148], postural control [1, 51], and gait [157]. These systems use force sensors to quantify forces exerted during performance of activities or tasks [32]. They are utilized by clinicians and researchers to assess functional progression throughout rehabilitation and may assist in determining the ability to RTS in post-ACLR individuals [64]. Previous studies have examined various kinetic parameters in the ACLR population; however, there is a lack of consistency in the literature regarding which parameters to assess and what assessment protocol(s) to follow. Thus, the primary objectives of this scoping review were to (1) describe the approaches for kinetic measurements in individuals following primary ACLR and (2) propose methodological reporting considerations for future studies. The secondary objective was to explore how commonly kinetic measurement system findings were related to PROMs. This review will provide clinicians and researchers with further information on the use of kinetic measurement systems in the ACLR population and may also inform future studies which, ultimately, may advance this field of study.

## Methods

The current review followed the six-stage methodological framework by Arksey and O’Malley (Table 1) [7] while considering the recommendations by Levac et al. [93], and the Joanna Briggs Institute Manual for Scoping Reviews [134]. It was conducted and reported according to The PRISMA Extension for Scoping Reviews (PRISMA-ScR) [169]. The current refined review’s protocol was uploaded on the University of Alberta Education and Research Archive: https://doi.org/10.7939/r3-e9fz-et12.

**Table 1:** Arksey and O’Malley 6-stage methodological framework

Stage 1	Identify the scope and inquiries
Stage 2	Identify data sources and search
Stage 3	Record screening and study selection
Stage 4	Data charting
Stage 5	Collate, summarize, analyze and report the results
Stage 6	Stakeholders’ consultation

### Stage 1: identifying the scope and inquiries

The primary research questions that guided this scoping review were:

What are the current approaches for kinetic measurements in individuals following ACLR?

Is there a need to propose methodological reporting considerations for future studies?

#### Eligibility criteria

All inclusion and exclusion criteria are reported in Table 2. The constructs of “participants”, “primary ACLR”, and “kinetic measurement systems” are operationalized in Table 3.

**Table 2:** Inclusion and Exclusion Criteria

Inclusion Criteria	Exclusion Criteria
Human participants	Animal models or cadavers
• Primary study design (quantitative & mixed methods) with original published data	• Qualitative studies and not primary study design or original data (conference proceedings or abstracts, editorials, commentaries, opinion-based papers and systematic, scoping, or narrative reviews) • Theses and Dissertations • Case Studies
• Studies with participants post-ACLR	• Studies with participants post-ACL repair (i.e., surgical reattachment of the ACL, instead of performing a reconstruction) [187]
• Studies with a population of primary ACLR participants	• Studies with only secondary ACLR participants • Studies where participants have other significant comorbidities, including; musculoskeletal, neurologic and/or systemic disorders • Studies where more than 50% of the participants had meniscal procedures at the same time as the ACLR
• Studies with kinetic measurement systems outcomes	• Studies with no kinetic measurement systems outcomes. Studies that included force plates only to confirm foot contact with ground (confirmatory kinetic measurement system)
• Only studies with extractable data of individuals who were at least 6 months following a primary ACLR (i.e., following completion of standard rehabilitation) were considered	• Reported data before 6 months post-ACLR
Theses and dissertations were excluded at the full-text review stage as many identified dissertations were published separately and included in this review.

**Table 3:** Definitions

*Participants*	Any individual with primary ACLR; no limitation to a specific age group, sex, sport or activity level.
*Primary ACLR*	A first time ACLR; surgical tissue graft replacement of the anterior cruciate ligament to restore its function after injury [100].
*Kinetic measurement systems*	This review included all platforms that use similar kinetic measurement systems technologies including force plates, balance platforms, pressure platforms, force measuring treadmills, Wii balance boards, contact mats connected to jump systems (computer software or device), and single-sensor insoles.

### Stage 2: identifying data sources and search

#### Information sources

Potentially relevant studies were identified through literature searches of the following electronic databases: MEDLINE (Medical Literature Analysis and Retrieval System Online), EMBASE (Excerpta Medica dataBASE), CINAHL (Cumulative Index of Nursing and Allied Health Literature), SPORTDiscus, Scopus, Web of Science, and ProQuest Dissertations and Theses Global for unpublished theses. These databases were searched since inception with no language limitations.

#### Search strategy

The search strategy was developed by an experienced librarian scientist (LD) with refinement of the search terms through iterative discussions between the study team and research collaborators to ensure identification of relevant records. The search terms included keywords and subject headings (MeSH) that have emerged in this research field, as appropriate. (Supplementary File 1 shows the search strategy.)

### Stage 3: record screening and study selection

Potentially relevant records were exported into a reference management software (EndNote X9.3.3) where duplicates were removed [25]. The titles and corresponding abstracts of remaining records were independently screened by 2 raters (WL, MMS) using Covidence (Veritas Health Innovation, Melbourne, Australia; available at www.covidence.org). Initially, the 2 raters (WL, MMS) independently screened a random sample of 100 titles and abstracts to assess the appropriateness of the selection criteria and determine the inter-rater agreement between reviewers using a Microsoft® Excel workbook explicitly designed for screening [175]. The raters reached substantial agreement (Cohen Kappa 90% = 0.75; 95% CI 0.60–0.90). The study team further refined the selection criteria prior to commencing full title and abstract screening. Finally, the 2 raters independently performed full-text review to determine final study selection. Disagreement on study eligibility during the title and abstract screening and full-text review stages were resolved through discussion between the two raters; a third rater (MFS) was approached if necessary, until consensus was reached.

### Stage 4: data charting

Table 4 outlines the data items extracted from each study. Prior to data extraction, the form was assessed through comparison of data extracted by the 2 raters independently (WL, MMS), using a purposive sample of 10 studies of various designs. Discrepancies in charted data were resolved through discussions between raters.

**Table 4:** Data Items

Category	Item(s)
Study characteristics	Author(s), year of publication, language, study design and location of investigation
Study objectives	Study objectives and purposes
Participant sample characteristics	Sample size disaggregated by sex, age, reported activity and activity level
Primary ACL surgical details	Graft type, side of surgery (dominant/non-dominant), time from surgery
Testing protocol details	Activity measured or assessed (jumping/landing, balance, gait, or other functional activities) Testing equipment used (force plate, balance platform, etc.), sampling frequency, testing protocol and tasks performed, number of trials per test
Outcomes	Testing equipment parameters, clinical assessment tools Self-reported outcome measures related to function, physical activity, readiness to return to sports, quality of life, and kinesiophobia

### Stage 5: collate, summarize, analyze and report the results

We conducted a descriptive and numerical analysis of the extracted variables. To align our results with our research questions, we collected the reported objectives and methods for each paper and categorized the outcomes (parameters) based on the movements assessed by the kinetic measurement systems (i.e., jumping, landing, step-over, stop-jump, lunges, cutting movement, squatting, gait, and standing balance). We reported the parameters as defined by the authors of the included studies. We recorded testing protocols, including: the testing environment setup, participants’ preparation, testing conditions, protocol details, number of repetitions, and duration of tasks, as applicable (see Supplementary File 2). We also identified studies that included PROMs and kinetic measurement system parameters. An iterative process was followed to suggest methodological reporting considerations. Specifically, the primary author (WL) drafted methodological reporting considerations based on study findings and team recommendations. Subsequently, the study team met and provided comments and feedback, resulting in the final version of the suggested methodological reporting considerations.

### Stage 6: consultation

To employ an integrated knowledge translation and dissemination approach, we engaged a knowledge user (a biomechanist) and a research collaborator (an engineer with expertise in force plates and balance platforms) for their input on the study findings.

## Results

### Identification of studies

**Fig. 1:**
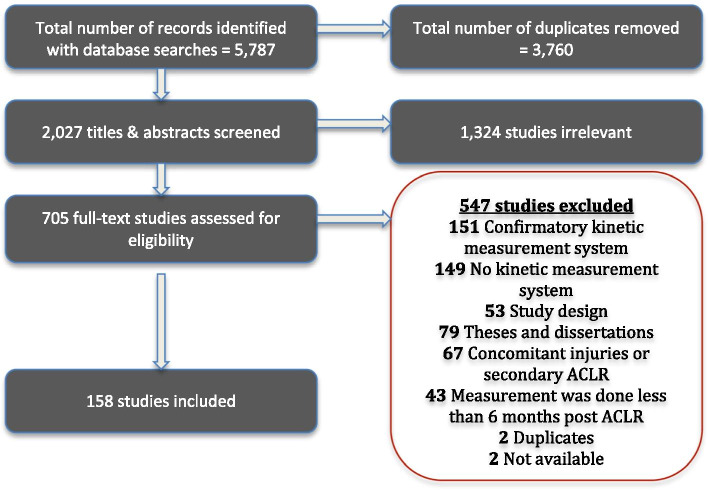
Search Results and Study Selection

An overview of the study identification process is provided in Fig. 1. Of 5787 identified records, 2027 unique records underwent title/abstract screening, 705 were reviewed in full, and 158 studies were included. Papers evaluating the same cohort with different (a) aims, (b) tasks evaluated, or (c) outcomes were treated independently.

### Characteristics of studies

The characteristics of the 158 included studies are summarized in Supplementary File 2. All studies were quantitative including 111 (70.25%) cross-sectional studies, 35 (22.15%) longitudinal, 10 (6.33%) interventional, and 2 (1.27%) case-control studies. Studies were published between 1990 and 2020 with 99 (62.66%) studies published since 2015. Studies were conducted in 28 countries with the highest number conducted in the United States (58 [36.7%]). Overall, 7909 participants were included (female = 2787 [35.2%]; male = 5122 [64.8%]). The mean age of participants ranged from 15.6 (± 1.7) to 48.2 (± 5.5) years. 5570 participants (70.4%) had ACLR, 158 (2.0%) were ACL deficient (ACLD), and 2331 (27.6%) were healthy controls. Participants represented a variety of physical activity and sport participation levels. Healthy control groups existed in 91 (58%) studies.

### Movement tasks

We identified 7 different types of kinetic measurement systems that assessed 9 different movements (tasks) in 4 main categories: landing/jumping, standing balance, gait, and other functional tasks. Table 5 contains full descriptions of movements and categories, identified kinetic measurement systems, and frequency of use across the studies. The majority of studies assessed landing, jumping, standing balance, and gait parameters. The force plate was the most commonly used system and the only system with potential to measure all identified movement tasks.

**Table 5:** Frequency of different kinetic measurement systems used to assess different movements (tasks) across the studies

Kinetic Measurement Systems	Movement Tasks								
	Landing/Jumping		Standing Balance	Gait	Other Functional Tasks				
	Landing	Jumping			Cutting Movement	Squatting	Stop Jump	Step-Over	Lunges
Force Plates	56^a^	6^a^	23	26	8	5	2	1	1
Balance Platforms	0	1	28	0	0	0	0	0	0
Pressure Mats	0	1	2	1	0	1	0	0	0
Force Measuring Treadmills	0	0	0	6	0	0	0	0	0
Wii Balance Board	0	0	4	0	0	0	0	0	0
Contact Mats Connected to Jump system	0	3	0	0	0	0	0	0	0
Single-Sensor Insoles	2	0	0	0	0	0	0	0	0

^a^Three studies assessed both jumping and landing and were included under both “Landing” and “Jumping” columns, bringing the total number of studies assessing landing and jumping using force plate to 59

Data was collected and reported, where possible, for the parameters identified, system setup (kinetic measurement system type, sampling frequency), participants’ preparation (warm up, barefoot/shoed, hand position), and protocol details (movement platform, movement direction, movement type, single/double-leg jumping, single/double-leg landing, task after landing, eyes open/closed, single/dual task, number of repetitions). Overall, there was substantial heterogeneity among studies in the parameters examined and the protocols used. Below, we summarize the identified parameters and protocols according to the 4 main movement categories.

#### Landing/jumping

Sixty-six studies examined landing and/or jumping tasks, with 43 (65.2%) published during the last 5 years. Studies included data from 3307 participants: 981 (29.7%) females, 2326 (70.3%) males; 2170 (65.6%) ACLR, 64 (1.9%) ACLD, and 1073 (32.4%) healthy controls.

Fifty-three unique kinetic variables were identified using 5 different measurement systems (force plates, contact mats connected to jump systems, single-sensor insoles, balance platforms, and pressure mats; Table 5). The following sections describe the parameters identified (as defined and reported by the authors of the included studies) and the measurement protocols used for each measurement system.

*Force Plate (Measurement System 1):* Force plates were used in 59/66 (89.4%) studies. Of the 59 included studies, 53 (89.8%) assessed landing only [12, 13, 30, 31, 41, 43, 49, 52, 54–56, 60–62, 68, 76, 79, 80, 84–90, 102, 103, 107, 110, 115–118, 120, 127, 129, 142, 146, 149, 150, 153, 156, 158–160, 163, 170, 172, 176, 177, 181, 182, 184], 3 studies assessed jumping only [16, 53, 144], and the remaining 3 studies assessed jumping and landing together [83, 114, 122].

*Force Plate Parameters:* Forty-six unique parameters were identified. Vertical Ground Reaction Force (vGRF) and peak vGRF were the most frequent parameters, each identified in 16 (27.1%) [30, 43, 49, 55, 68, 79, 80, 115, 127, 146, 153, 158, 160, 163, 172] 15 (25.4%) [30, 31, 41, 84, 102, 110, 116, 117, 122, 149, 159, 160, 172, 176, 181, 182] and 15 (25.4%) [30, 31, 41, 84, 102, 110, 116, 117, 122, 149, 159, 160, 172, 176, 181, 182] studies, respectively, followed by the peak Ground Reaction Force (GRF) in 6 (10.2%) studies [52, 62, 110, 118, 122, 184]. The remaining parameters were each measured between 1 to 5 times with a median of 1. (Supplementary File 2-Table 1).

*Testing Protocol:* This includes system setup, participants’ preparation, and jumping/landing protocol details.

Related to *system setup,* force plate sampling frequencies were reported in 48 (81.4%) studies and ranged between 50 Hz and 5000 Hz. The most frequently used sampling frequencies were 1000 Hz and 1200 Hz in 17 (28.8%) [16, 52, 53, 68, 76, 79, 80, 83, 120, 142, 144, 146, 150, 158–160, 163] and 12 (20.3%) studies [13, 43, 49, 54, 102, 103, 115, 116, 127, 149, 172, 176], respectively.

Regarding *participants’ preparation,* participants were asked to warm up prior to testing in 18 (30.5%) studies [16, 31, 43, 49, 53, 55, 68, 76, 79, 80, 85, 86, 107, 117, 118, 146, 163, 184]. There was substantial heterogeneity in warm up duration and components across the studies. Participants were barefoot in 5 (8.5%) studies [16, 68, 102, 103, 129], and wore shoes in 20 (33.9%) studies [13, 49, 52, 60, 61, 76, 79, 80, 84–86, 88, 89, 120, 146, 149, 159, 176, 177, 181]. The remaining 34 (57.6%) studies did not specify whether participants wore shoes or not [12, 30, 31, 41, 43, 53–56, 62, 83, 87, 90, 107, 110, 114–118, 122, 127, 142, 144, 150, 153, 156, 158, 160, 163, 170, 172, 181, 184]. Of 22 (37.3%) papers that reported hand placement while testing, 18 studies requested participants to keep hands on hips [16, 41, 49, 53, 56, 68, 76, 79, 83, 88, 89, 120, 129, 142, 144, 172, 181, 182], 2 instructed participants to cross their arms on their chest [43, 60] and 2 studies, by the same author, had participants hold a short rope behind their back [102, 103].

Finally, *jumping/landing protocols* varied substantially in terms of the jumping platforms, jumping directions, type of jump, number of jumping/landing tasks per study, use of single−/double-leg to jump or land, movement after landing, and number of trials.

Different *jumping platforms* were used across the included studies*.* In 37 (62.7%) studies [12, 13, 30, 31, 41, 43, 49, 54–56, 60, 62, 68, 76, 79, 80, 87, 90, 107, 115, 116, 127, 129, 146, 149, 150, 153, 156, 158–160, 170, 172, 176, 181, 182], participants jumped off a box that ranged in height from 10 to 60 cm, with a median height of 30 cm, onto force plates. The box was placed just behind the force plate in 27 (45.8%) studies [41, 43, 49, 54, 56, 60, 62, 68, 76, 79, 80, 87, 107, 114–116, 127, 129, 146, 150, 156, 158–160, 170, 181, 182], and at a distance that ranged between 10 cm to 50% of participant’s height in 10 (16.9%) studies [12, 13, 30, 31, 55, 90, 149, 153, 172, 176]. Participants in 21 (35.6%) studies jumped from the floor [12, 13, 30, 31, 53, 55, 83–86, 88–90, 117, 118, 142, 144, 149, 153, 172, 176], and from an inclined surface in 1 study [52]. The horizontal distances between the starting line and the force plates was reported in only 4 (18.2%) studies and varied substantially (100 cm [110], 70 cm [177], 75% of the body height [120], and a predetermined maximum distance [61]).

Likewise, different *jumping directions* were reported across the studies. Participants dropped/stepped down off a box onto a force plate in 25 (42.4%) studies [41, 43, 49, 54, 56, 60, 62, 68, 76, 79, 80, 87, 107, 114–116, 127, 129, 146, 150, 156, 158–160, 170], jumped forward off a box onto a force plate in 10 (16.9%) studies [12, 13, 30, 31, 55, 90, 149, 153, 172, 176], jumped forward from the floor in 7 (16.9%) studies [16, 61, 69, 110, 120, 122, 184], jumped to the side in 3 (5.1%) studies [102, 103, 163], and jumped vertically from the floor, from a box, and from an inclined surface in 11 (18.6%) [53, 83–86, 88, 89, 117, 118, 142, 144], 2 (3.4%) [181, 182], and 1 (1.7%) study [52], respectively.

While most studies (*n* = 43 [72.9%]) assessed only 1 jumping/landing task [12, 13, 16, 30, 41, 43, 49, 53–56, 60–62, 68, 76, 83, 87–90, 102, 103, 107, 110, 115–117, 120, 127, 142, 144, 149, 153, 158–160, 170, 172, 176, 177, 181, 184], 12 (20.3%) studies assessed 2 tasks [31, 84–86, 114, 118, 122, 129, 150, 156, 163, 182], 1 (1.7%) study assessed 3 tasks [146], and 3 (5.1%) studies assessed 4 tasks [52, 79, 80].

Of the studies that reported on jump type, 8 studies requested participants to perform counter movement jumps (CMJ) [24, 83, 88, 89, 117, 118, 142, 144], 3 required participants to perform squat jumps [16, 52, 53], 1 study reported vertical jumps while not allowing for countermovement [86], and 3 studies requested participants to do lateral jumps over hurdles of different heights (15 to 24 cm) and then rebound [102, 103, 163]. Of those studies that performed a drop landing, only 2 studies instructed participants to land on their toes [49, 107].

In studies reporting landing on 2 legs (*n* = 32 [54.2%]), participants took-off from double- and single-leg stances in 27 studies [12, 30, 31, 43, 49, 52, 54, 55, 62, 68, 83–86, 88–90, 107, 110, 114, 116, 127, 144, 146, 149, 150, 176] and 3 studies [158–160], respectively. The remaining 2 studies did not report on the take-off stance position [79, 80]. When landing on a single leg (*n* = 25 [42.4%]), 18 studies reported jumping from a single-leg stance position [16, 41, 53, 56, 61, 76, 102, 103, 115, 120, 122, 142, 156, 163, 170, 181, 182, 184], 6 studies reported jumping from a double-leg stance position [60, 117, 118, 129, 172, 177], while 1 study did not report the starting position [153]. After landing, activities varied across studies according to the landing strategy (i.e., single- vs. double-leg landing). Maintaining balance was most commonly reported after landing on a single leg (10/25 studies [40.0%]) [87, 102, 103, 129, 142, 156, 177, 181, 182, 184] while maximum vertical jump was the most reported activity performed after landing on both legs (19/32 [59.4%]) [12, 30, 31, 54, 55, 62, 68, 79, 88, 89, 114, 116, 127, 149, 158–160, 170, 176]. Other activities such as “cut and run” [110] or “pivot and run” [90] were each reported once, following participants’ landing on both legs. Repetitions/trials were completed between 1 and 10 times across studies, with a median of 3 trials per study. (Supplementary File 2-Table 1).

*Balance Platforms (Measurement System 2):* One study assessed jumping using a balance platform to identify the number of jumps and the peak and minimum values of GRF [39]. Jumping was performed on a single leg with no information given on testing conditions. (Supplementary File 2-Table 1).

*Pressure Mats (Measurement System 3):* One study used pressure mats to assess peak load and flight time during jumping. Participants were requested to jump barefoot on single and double legs [40]. No further information was provided regarding warm up or testing conditions. (Supplementary File 2-Table 1).

*Contact Mats and Jump Systems (Measurement System 4):* Three studies reported on contact mats synchronized with jump systems (i.e., computer software or device) to assess jumping [24, 125, 135]. Jump height [24, 135], total power [24], relative power [24], and limb symmetry index [125] were the 4 unique parameters identified.

Several protocol items were inconsistent across 2 studies [24, 135], while 1 study did not provide protocol information [125]. Two studies did not report whether participants warmed up or not, were shoed or barefoot, nor did they discuss hand placement. One study described the jumping activity as 3 consecutive double-leg CMJs with the aid of the arms with a 10-s break between trials [24]. The other study had participants perform 3 10-s jumping trials (for maximum number and height possible) while keeping hands on hips [135]. The best trials were used for analysis in both studies. (Supplementary File 2-Table 1).

*Single-Sensor Insoles (Measurement System 5):* Two recent studies using the same cohort of individuals with ACLR and healthy controls reported on single-sensor insoles to assess landing [130, 131], using the same variables and protocols to address different aims. One evaluation compared knee bracing and no bracing conditions during landing [130], while the other compared hop distance and loading symmetry [131]. Participants were requested to hop as far as possible taking off and landing on 1 leg (single hop), to hop 3 consecutive times (triple hop), and to hop 3 consecutive times while laterally crossing over a 6-in.-wide strip with each hop and progressing forward. Each test was repeated twice [130, 131]. (Supplementary File 2-Table 1).

#### Standing balance

We identified 57 studies examining standing balance published between 1994 and 2020, with 28 (49.1%) papers published since 2015. These studies included 3173 participants; 1206 (38.0%) females, 1967 (62.0%) males; 2148 (67.7%) ACLR, 103 (3.2%) ACLD, and 922 (29.1%) healthy controls.

Forty-eight balance parameters were identified using 4 different kinetic systems (force plates, balance platforms, Wii balance boards, and pressure mats). Each protocol described the kinetic measurement systems used, participant preparation (barefoot or shoed), standing position (single−/double-leg stance, hand placement, looking at a target (yes/no)) and testing conditions (eyes open/closed, single/dual tasks, static/dynamic task).

*Force Plate (Measurement System 1):* Of 57 studies assessing standing balance, 23 (40.3%) used force plates [1, 14, 21–23, 26, 45–47, 51, 55, 56, 59, 64–67, 87, 123, 136, 165, 166, 187]. Center of pressure (CoP) velocity was the most commonly measured parameter (*n* = 10 [43.4%]) [1, 21, 22, 26, 45, 51, 55, 59, 123, 187]. CoP displacement in anterior-posterior and medio-lateral directions [1, 14, 22, 23, 51, 123], and CoP length of path [14, 67, 87, 136, 165, 166] were the second most frequently used parameters, where each was measured in 6/23 (26.1%) studies. The CoP sway area was measured in 5/23 (21.7%) studies [1, 45, 51, 59, 136]. The remaining parameters were each used in 1 to 2 of the 23 studies. (Supplementary File 2-Table 2).

*Testing Protocol:* This includes system setup, participants’ preparation and balance testing protocol details. Protocols for measuring standing balance using force plates demonstrated limited consistency across studies and lack of reporting for important items. The following sections discuss consistency or lack thereof in protocol reporting.

*System setup* varied among studies assessing balance using force plates. While most studies reported asking participants to stand directly on the force plate, 1 study placed foam [14] and another placed a wobble board on top of the force plate [1]. Force plates were sampled at frequencies ranging from 40 Hz to 2000 Hz, with a median of 100 Hz. Three studies did not report the frequency used [59, 66, 187].

Likewise, *participants’ preparation* varied amongst studies and lacked detailed reporting. Warm-up sessions were reported in 6 (26.1%) studies [45, 55, 64, 65, 67, 187]. Participants were requested to be barefoot in 10 (43.5%) studies [23, 46, 47, 51, 55, 123, 136, 165, 166, 187], shoed in 1 (4.3%) study [65], while the remaining 12 (52.2%) studies did not report this detail [1, 14, 21, 22, 26, 45, 56, 59, 64, 66, 67, 87]. Hand placement was also inconsistent; hands were placed on the hips in 7 (30.4%) studies [45, 55, 56, 64–67], crossed on the chest in 6 (26.1%) [22, 23, 26, 59, 165, 166], placed free at the side of the body in 5 (21.7%) [46, 47, 51, 187], and not reported in the remaining 5 (21.7%) studies [1, 14, 21, 87, 123].

Finally, *balance testing protocols* were heterogeneous in terms of testing conditions (single−/double-leg stance, focusing on a target or not, eyes open/closed, single/dual tasks). Standing balance was assessed under both single- and double-leg stance in 5 (21.7%) studies [22, 23, 46, 47, 51] and in double-leg stance in 3 (13.0%) studies [1, 136, 165]. The remaining 15 (65.2%) studies assessed single-leg standing balance only [14, 21, 26, 45, 55, 56, 59, 64–67, 87, 123, 166, 187]. Participants were asked to look at a target in 7 (30.4%) studies [1, 51, 56, 64, 67, 123, 166]. In 6 (26.1%) studies, balance was tested in eyes open and closed conditions [14, 26, 66, 123, 136, 187], while 5 (21.7%) studies assessed balance under eyes closed conditions only [22, 23, 45, 55, 59, 165], and the remaining 11 (47.8%) studies had the participants’ eyes open. Most studies (22/23 (95.6%)) assessed balance using a single task [1, 14, 21–23, 26, 45–47, 51, 55, 56, 59, 64–67, 87, 123, 136, 165, 166, 187]; only 1 study used dual tasks (a concurrent physical and cognitive task) [1]. (Supplementary File 2-Table 2).

*Balance Platforms (Measurement System 2):* Balance platforms were used in 28 studies [2–4, 10, 11, 44, 57, 63, 73, 81, 92, 95, 105, 111, 112, 119, 121, 124, 126, 128, 140, 141, 147, 161, 171, 173, 185, 186]. Stability index was the most widely used parameter, reported in 12 (42.9%) studies [10, 11, 92, 95, 105, 111, 112, 124, 140, 173, 185, 186], followed by anterio-posterior and medio-lateral stability indices, reported in 9 (32.1%) studies [3, 10, 105, 111, 112, 124, 140, 173, 185]. The remaining parameters were reported only 1 to 4 times across all studies. (Supplementary File 2-Table 2).

The *testing protocols* for balance platforms were described in all but one study [186]. In general, most protocols included information on participants’ preparation, and the testing protocol used. However, many studies did not report important protocol items.

With regard to *participants’ preparation*, participants had warm-up sessions in 3 (10.7%) studies [73, 105, 185]. They were requested to participate barefoot in 11 (39.3%) studies [2, 3, 10, 57, 92, 95, 111, 112, 121, 124, 126] and remain shoed in 1 study [128]. The remaining 16 (69.6%) did not specify whether participants were barefoot or not. Further, of 13 (46.4%) papers reporting hand position, 7 studies requested participants to cross arms on chest [3, 63, 73, 119, 128, 140, 171], 4 placed hands on hips [92, 95, 111, 112] and 2 studies reported participants’ hands hanging by their sides [10, 57].

The *testing conditions and protocols details* were heterogeneous and lacked sufficient reporting when assessing standing balance using balance platforms systems. The majority of papers (*n* = 17 [67.9%]) reported assessing single-leg standing balance [2, 3, 63, 73, 81, 92, 95, 111, 112, 119, 121, 124, 126, 128, 140, 161, 185], while 5 (17.9%) assessed balance in double-leg stance [4, 11, 57, 141, 173] and 5 (10.7%) reported investigating balance in both conditions [10, 44, 105, 147, 171]. Only 6 (21.4%) studies compared standing balance under eyes open and closed conditions [2, 63, 81, 111, 112, 126], while 11 (47.8%) papers had participants focusing on targets while attempting to maintain balance [3, 63, 73, 95, 105, 111, 112, 126, 161, 171, 173]. Most studies (*n* = 20 (71.4%)) assessed either static (*n* = 9 (32.1%)) [2, 63, 73, 95, 121, 126, 140, 141, 161], or dynamic balance (*n* = 11 (39.3%)) [3, 4, 10, 92, 105, 111, 112, 119, 124, 128, 173], while 7 (25.0%) studies compared both conditions [11, 44, 57, 81, 147, 171, 185]. Only 1 study added a cognitive task while participants were trying to maintain balance [2]. (Supplementary File 2-Table 2).

*Wii Balance Boards (Measurement System 3):* Wii balance boards were utilized to assess standing balance in 4 (7.0%) studies published between 2013 and 2017, reporting 8 different parameters [34, 35, 38, 70]. CoP displacement in anterior-posterior and medio-lateral directions [34, 38], CoP length of path [34, 70], CoP velocity [35, 38] and standard deviation [35, 38] were each calculated in 2 (50%) studies. Other parameters such as CoP amplitude [35], CoP fast/slow sway [34], discrete wavelet transform and sample entropy of the CoP trace [35], were each calculated once across studies. (Supplementary File 2-Table 2).

There was reasonable consistency among the 4 reported *testing protocols*. Participants were barefoot in all studies. Hands were placed on hips in 2 studies [35, 70], crossed on chest in 1 study [38], and not reported in the remaining study [34]. In 1 (25.0%) study, participants were asked to move their arms to measure balance under a dual task condition [70]. All participants had their eyes open; however, in 2 studies, they were instructed to look forward at a target [35, 70]. Three studies [35, 38, 70] investigated single-leg balance and 1 study assessed double-leg balance [34].

*Pressure Mats (Measurement System 4):* Pressure mats were used by only 2 (3.5%) studies to assess standing balance [33, 82]. Five parameters were identified including ellipse area [33, 82], CoP standard deviation in anterior-posterior and medio-lateral directions, CoP path length, CoP velocity, and sway area [82].

While participants in 1 study were barefoot [82], the other study did not report whether they were shoed or not [33]. Likewise, 1 study reported the arms being free at participants’ sides [33], while the other didn’t specify [82]. Participants in both studies were asked to look forward during testing; however, 1 study also assessed balance under an eyes-closed condition [33]. Both studies investigated balance in both single- and double-leg stances. (Supplementary File 2-Table 2).

#### Gait

Thirty-three studies examining gait were published between 1997 and 2020 with 27 (81.1%) published since 2015. They represented data from 1261 participants: 708 (56.1%) males, 553 (43.9%) females, 1059 (84.0%) ACLR, 10 (0.8%) ACLD, and 192 (15.2%) healthy controls.

Forty-four unique variables were identified to assess gait using 3 different systems (force plates, force-measuring treadmills, and pressure mats; Table 5). The following section discusses the parameters identified, and the measurement protocols used for each of those systems including, where applicable; system setup (sampling frequency), participants’ preparation (barefoot/shoed) and protocol details (self-selected/predetermined speed, single/dual task, testing condition, distance and duration). (Supplementary File 2-Table 3).

*Force Plates (Measurement System 1):* Force plates were used in 26 (78.8%) studies. Overall, there was a lack of consistency in the measured parameters across studies using force plates to assess gait. Important protocol items such as gait speed and shoe wear conditions were reported in 20/26 (70%) [17, 19, 20, 27, 72, 99, 101, 109, 114, 133, 137–139, 154, 155, 159, 168, 179, 180, 183], and 14/26 (53.8%) [17, 18, 72, 99, 109, 133, 137–139, 150, 159, 164, 179, 180], respectively.

*Force Plates Parameters in Gait Assessments:* Thirty-six parameters were identified in the 26 studies that assessed gait using force plates. Peak vGRF was the most frequently measured variable in 8 (30.8%) studies [18–20, 72, 138, 139, 159, 168], followed by vGRF, which was measured in 6 (23.1%) studies [109, 154, 164, 179, 180, 183]. (Supplementary File 2-Table 3).

*Gait Testing Protocol:* This includes force plate system setup, participants’ preparation, as well as the gait testing protocols using force plates. Regarding *system setup*, the sampling frequency was reported in 22 (84.6%) of 26 studies [9, 17–20, 27, 36, 72, 94, 99, 101, 133, 138, 139, 149, 150, 154, 155, 159, 164, 168, 183]. Sampling frequency ranged between 400 Hz and 1200 Hz with a median of 1080 Hz. The most commonly reported frequencies were 1200 Hz and 1000 Hz in 9 [17–20, 72, 94, 99, 138, 149] and 5 studies [9, 139, 150, 159, 168], respectively.

Related to *participants’ preparation,* only 2 studies reported asking participants to warm-up prior to testing [94, 101], and only half of the studies reported whether their participants were shoed (*n* = 3) [133, 159, 164] or not (*n* = 10) [17, 72, 99, 109, 138, 139, 149, 150, 179, 180].

Different *testing conditions and protocols* were followed across the studies. Most studies assessed only walking gait (*n* = 21 (80.8%)) [9, 17–20, 36, 72, 99, 101, 138, 139, 149, 150, 154, 155, 159, 168, 179, 180, 183], while 2 (7.7%) studies assessed only running gait [133, 164], and 3 (11.5%) studies assessed both walking and running gaits [94, 109, 114]. Of 21 (80.8%) studies that reported speed, 16 (76.2%) reported that participants walked at a self-selected speed [17, 19, 20, 27, 36, 72, 101, 109, 133, 138, 139, 149, 154, 179, 180, 183], while 4 (19.0%) studies used a pre-determined speed [99, 155, 159, 168], and 1 (4.8%) study indicated testing participants in both conditions [114]. No study tested gait in a dual task condition. Participants in 5 (19.2%) studies were asked to look forward at a target [36, 99, 133, 138, 139]. Walking distance greatly varied in the 6 (23.1%) studies, with reported distances ranging from 3 to 20 m (median = 6.5 m) [17, 18, 27, 101, 138, 139]. (Supplementary File 2-Table 3).

*Force-Measuring Treadmills (Measurement System 2):* Six studies used force-measuring treadmills in gait assessment and reported 8 *parameters* including vGRF [50, 58, 98], vGRF limb symmetry index [98], peak vGRF [96, 97], peak vGRF normalized to body weight [113], instantaneous vGRF loading rate [96, 98], instantaneous vGRF loading rate normalized to body weight [97] instantaneous vGRF loading rate limb symmetry index [97], and root mean square error between actual vGRF and biofeedback target vGRF [96].

*Testing protocols* and reporting standards varied among the studies measuring gait using force-measuring treadmills. Only 1 study reported a warm-up session prior to testing [58]. Two studies reported that participants had their shoes on during testing [58, 113] while the remaining studies did not specify [50, 96–98]. While 4 studies examined walking at a predetermined speed [50, 96–98], 1 study assessed walking and running at a predetermined speed [113], and 1 study assessed running at a self-selected running speed [58]. Only 1 study assessed gait with and without real time biofeedback about participants’ GRF (as a dual task and a single task) [96]. (Supplementary File 2-Table 3).

*Pressure Mats (Measurement System 3):* One study used a pressure mat with a sampling frequency of 150 Hz to identify spatiotemporal parameters including velocity, cadence, step length and width. Participants walked at both self-selected normal and fast speeds for 8.5 m. It was not specified whether participants were shoed or barefoot [8]. (Supplementary File 2-Table 3).

#### Other functional movements

In addition to the aforementioned movement tasks, our review identified papers assessing other functional movements including; *cutting movements, squatting, stop-jumps, step-overs, and lunges.*

First, *cutting movements:* Eight studies used *force plates* to assess *cutting movements* (change in direction) kinetics. They were published between 2011 and 2020, with 6 (75%) papers published in the last 5 years. The studies represented data from 536 participants: 404 (75.4%) male, 132 (24.6%) female; 386 (72.0%) ACLR, 10 (1.9%) ACLD, and 140 (26.1%) healthy controls.

Nine different *parameters* were identified, mostly related to GRF. Identified parameters included GRF [78, 94], time to peak GRF [15, 110], peak vGRF [30, 31, 110], peak vGRF normalized to body weight [110], vGRF loading rate [30], vGRF normalized to body weight (in vertical, medial and posterior directions) [80], and Lyapunov exponent [91]. (Supplementary File 2-Table 4).

*Testing protocol items:* Sampling frequencies used were heterogeneous, ranging between 1000 Hz to 5000 Hz with a median of 1200 Hz. Protocols were also heterogeneous with several studies not reporting on important protocol items. For example, of 8 studies, only 5 (62.5%) reported having participants warm-up prior to testing [30, 31, 78, 80, 94], and only 3 (37.5%) reported that participants wore shoes [15, 78, 80]. The movements or conditions preceding the cutting movement (jumping over a hurdle [30, 31], standing [78], and landing after jumping [110]) were only reported in 4 studies, 2 of which were by the same author and included the same cohort [30, 31]. The cutting movement direction was planned in 3 (37.5%) studies [30, 31, 94], not planned in 1 (12.5%) study [110], while 2 (25%) studies (by the same author) tested cutting movements in both planned and unplanned conditions [78, 80]. One study investigated the effect of vision on participants’ performance by testing them under both full and disturbed vision conditions [15]. (Supplementary File 2-Table 4).

Second, *squatting:* Eight variables were identified in 6 studies that assessed squatting, utilizing 2 kinetic measurement systems; force plates were used in 5 (83.3%) studies [150–153, 178], and a pressure mat was used in the remaining study [40]. These papers were published between 2003 and 2020, with 4 [66.7%] published since 2015. The studies represented data from 207 participants (63 [30.4%] female, 144 [69.6%] male; 142 [68.6%] ACLR, 65 [31.4%] healthy controls).

*Force plates (Measurement System 1)* were used in 5 (83.3%) studies [150–153, 178]. Six different *parameters* were identified across studies including: first vertical maximum [150], peak vGRF [151], anterior-posterior GRF [152], medio-lateral GRF [152], vGRF [152, 153, 178], and weight bearing symmetry [178].

*Squatting testing protocol:* Overall, protocols of measuring squatting kinetics using force plates were heterogeneous and lacked sufficient reporting. Among the 5 studies using force plates, 2 reported the sampling frequency as 1000 Hz [150, 152], 2 did not report [153, 178], and 1 reported a sampling frequency of 600 Hz [151]. Only 1 study reported asking participants to warm up for 5 min on a stationary bike [151], and only 1 study reported that participants were barefoot [152]. Squatting speed was predetermined in 2 studies [150, 178], self-selected in 2 studies [151, 152], and not reported in the remaining study [153]. Participants squatted with both legs in 4 (80.0%) studies [150–152, 178], and with a single leg in 1 (20%) study [153]. The terminal squatting position was consistent across 3 studies, where participants were asked to descend until the posterior thigh was parallel to the floor [150, 151, 178]. In the remaining 2 studies, participants were asked to squat to a comfortable position while keeping the torso upright [152], or to squat as deep as possible [153]. (Supplementary File 2-Table 5).

*Pressure Mats (Measurement System 2):* One study used pressure mats to assess double- and single-leg squatting in barefoot participants [40]. The study did not report on the squat speed or the terminal squatting position. The pressure mat measured peak load while squatting. (Supplementary File 2-Table 5).

Third, *stop-jump:* Of the 158 studies, only 2 assessed a stop-jump task [143, 145]. The 2 papers represented data from 67 participants; 32 (47.8%) females, 35 (52.2%) males; 45 (67.2%) ACLR, and 22 (32.8%) healthy controls. The 2 papers reported using force plates to assess stop-jumps. Nine different *parameters* were identified including peak vGRF ratio index, peak vGRF gait asymmetry index, peak vGRF symmetry index, peak vGRF symmetry angle, peak vGRF normalized symmetry index [143], peak vGRF, peak posterior vGRF, loading rate, and impulse [145].

For the stop-jump task, there were several similarities in the study *testing protocol details*. In addition to using the same sampling frequency of 2400 Hz, participants in both studies were asked to approach the force plate as quickly as possible, stop, then jump as high as possible. No information was given about landing. Neither studies reported whether participants had a warm-up, or whether they had their shoes on or were barefoot [143, 145]. One study reported having participants jump off one foot, land on two, and perform a subsequent 2-footed jump [145]. (Supplementary File 2-Table 6).

Finally, *step-over and lunges* were both reported in only 1 paper which included 36 participants; 13 (36.1%) female, 23 (63.9%) male; 18 (50%) ACLR, and 18 (50%) healthy controls [104]. The study used a force plate for kinetic measurements. Three unique *parameters* were identified while performing the step-over task, including the lift up index, movement time, and impact index. In addition, the study reported 4 other parameters while performing lunge tasks including lunge distance, contact time, impact index, and force impulse [104].

For the *step-over* task, with shoes on, individuals were asked to perform a 5-min treadmill warm-up and then to step up onto a 30 cm box while the lagging leg was carried up and over to land on the opposite side of the starting position. For the *lunge* task, participants were requested to lunge forward with one leg on a long force plate and then return to the original standing position [104]. (Supplementary File 2-Table 6).

### Kinetic measurement systems and PROMs

Of 158 studies, only 6 studies reported on both kinetic measurement system findings and PROMs [9, 92, 98, 117, 158, 161]. The earliest study was published in 1996 and evaluated the association between standing balance and PROMs (Cincinnati Scale and satisfaction score) [161]. The remaining 5 studies were published in 2018 [9, 98], 2019 [92, 158] and 2020 [117]. There was inconsistency in the reported parameters and/or PROMs across these 6 studies (Table 6).

**Table 6:** Studies examined the association between kinetic measurement systems variables and PROMs

Reference	Studied Variables	
	Kinetic measurement systems variables	Patient Reported Outcome Measures
Shiraishi et al. 1996 [136]	CoP length of path	Cincinnati Scale & Satisfaction
Azus et al. 2018 [167]	GRF	Knee Injury and Osteoarthritis Outcome Score (KOOS)
Luc-Harkey et al. 2018 [172]	Peak vGRF normalized to body weight Instantaneous vGRF loading rate Linear vGRF loading rate vGRF LSI	Tampa Scale of Kinesiophobia
Lee et al. 2019 [123]	Overall Stability Index	Tegner Activity Scale
Shimizu et al. 2019 [68]	vGRF	KOOS & Marx Activity Scale Score
Niederer et al. 2020 [57]	LSI	Return to sport after injury-ACL (ACL-RSI) Fear of re-injury Visual Analog Scale (VAS)

### Methodological reporting considerations

Based on the substantial heterogeneity seen across studies in the methodological details and outcomes reported, we created a table of methodological reporting considerations for researchers designing studies using kinetic measurement systems (Table 7). The goal of this information is to improve standardized reporting of methodological approaches and kinetic measurements, which should facilitate cross-study comparisons to advance this burgeoning field of research and improve the clinical application of findings. We developed these methodological reporting considerations as they relate to the movement tasks, kinetic system features and selected outcome parameters, participant preparation, and protocol details.

**Table 7:** Methodological Reporting Considerations

Methodological Reporting Considerations				
Movement Tasks	Parameters	Testing Protocol Items		
Jumping/Landing	Definition Justifications of use	System Setup		System type Sampling frequency
		Participant preparation		Warm-up details Shoes/no shoes Hand placement
		Protocol details		Jumping platform Box (height and distance) Floor (distance from system) Inclined surface Jumping direction Drop jump/step down Forward jump Vertical jump Lateral jump Jump type (CMJ, squat, etc.) Number of jumping tasks Single/double leg Task after landing Number of trials
Standing Balance	Definition Justifications of use	System Setup		System type Platform surface Sampling frequency
		Participant preparation		Warm-up details Shoes/no shoes Hand placement
		Protocol details		Single/double leg stance Eyes open/closed Single/dual task Static/dynamic Task duration Number of trials
Gait	Definition Justifications of use	System Setup		System type Platform (floor, treadmill, etc.) Sampling frequency
		Participant preparation		Warm-up details Shoes/no shoes
		Protocol details		Speed Single/dual task Focus on a target Distance and duration
Other Functional Movements	Definition Justifications of use	System Setup		System type Platform surface Sampling frequency
		Participant preparation		Warm-up details Shoes/no shoes Hand placement
		Protocol details	Cutting	Movement preceding cutting Planned/unplanned movement Visual condition
			Squatting	Squatting speed Single/double leg Terminal squat position
			Stop-Jump	Landing condition after jumping Single/double leg Stop-jump procedure
			Step over and lunges	Step/hurdle height Step-over and lunges procedure

## Discussion

The primary purpose of this scoping review was to describe the approaches for kinetic measurements in individuals following ACLR. While force platforms can be used in conjunction with motion capture systems to measure kinetic variables such as joint moments, the intent of the study was to describe approaches and parameters using kinetic measurement systems only. Results of our evaluation demonstrate a substantial increase in the evaluation of kinetic measures in this patient group in recent years. Further, we noted marked heterogeneity in parameters evaluated and protocols followed, in addition to inconsistencies in reporting. In this review, we highlighted the current gaps in reporting and have generated a table of suggested methodological considerations to facilitate improved reporting when using kinetic measurement systems in the post-ACLR population.

In 1976, the first commercially available force plate was constructed to be used for gait analysis [71]. Technology advancements in recent years have facilitated kinetic assessments allowing more extensive measurement of movements/tasks. While the earliest included paper in this review was published in 1990, more than 66% of the included studies were published since 2015. This is likely related to the tremendous improvement in both hardware and software of kinetic technology. For example, advancement from uniaxial to triaxial force plates has allowed researchers and clinicians to evaluate variables such as multidimensional CoP displacement that cannot be measured with uniaxial force plate technology. Similarly, variables that integrate force and time, such as impulse and loading rate, would have been difficult to assess before recent technology developments that permit efficient calculations of large datasets.

However, with these advances have come a plethora of approaches and parameters to measure. This review identified important heterogeneity and methodological gaps in the current published literature that may limit the clinical application of this research. The first methodological gap is the inconsistency in the selection of parameters as well as their operationalization. For instance, some studies assessed jumping and landing using vGRF only, while others measured both vGRF and posterior GRF, without justifying their selection. All selected parameters may have relevance, but researchers should justify their selection to readers in light of their objectives. The lack of operationalization of commonly reported parameters also creates confusion. For example, using “vGRF” and “peak vGRF” made it challenging to discern if these parameters were the same or different measures across studies (i.e., did “vGRF” consider multiple points in time across the force-time curve, or only the time at which maximal vGRF was achieved?). Together, the heterogeneity and the lack of operationalization for evaluating specific parameters makes it challenging to determine the most clinically relevant parameters in the ACLR population.

The second methodological gap was the heterogeneity in the kinetic measurement systems setup, as the type of selected system and sampling frequency varied across studies assessing the same task(s). Other important methodological gaps include the inconsistency in reporting important protocol items such as participant preparation (e.g., warm-up details, hand position, shoed vs. barefoot) and protocol details (e.g., starting/ending positons, eyes open vs. closed, and single vs. double-leg landing). These methodological considerations can influence the reported outcomes. For instance, a gluteal warm up program may enhance force production while performing squat jumps after 8 min of recovery [167]. Similarly, arm swings while performing vertical counter movement jumps can increase jump height by 38% [174]. Therefore, when assessing a task such as CMJs using a force plate, our methodological reporting consideration may guide future papers to define the parameters of interest, justify parameter selections, report on the force plate details, and report the sampling frequency used. Authors should also report warm-up program details, whether participants were shoed or not, and participants’ hand placement while performing the CMJs. When reporting on the CMJ activity, we recommend authors report on the direction of jump, single−/double-leg jumping or landing, and the immediate tasks performed after landing. Researchers need to consider and justify their approaches a priori and ensure that they report them as such. Our findings underscore the need to develop standardized reporting guidelines to enhance the quality of future studies and advance this field of research.

Though we aimed to describe the use of kinetic measurement systems in post-ACLR individuals, it was not our intent to make recommendations regarding which kinetic parameters to examine to inform RTS decisions following ACLR. We did not examine reported outcomes in our included studies, but rather conducted a detailed review of the reported approaches. The findings from the current review may have implications for future research and, consequently, clinical application. The suggested methodological considerations (Table 7) will assist in standardizing the reporting of important protocol details in future studies, to allow future meta-analyses which may better inform clinical practice.

The secondary purpose of the current review was to explore papers studying potential associations between kinetic measures and PROMs. Our findings highlighted an evidence gap as we identified only 6 studies that investigated this potential relationship [9, 92, 98, 117, 158, 161]. The identified studies demonstrated inconsistencies in the parameters measured and the types of PROMs utilized. Of the 6 studies, 5 were published since 2018 [9, 92, 98, 117, 158]. This may indicate an emerging research area acknowledging psychosocial factors that may interact with kinetic measurement outcomes; future studies are needed to further understand the extent of this relationship. Due to the heterogeneity in kinetic parameters and PROMs used, and the limited number of papers identified, a systematic review to examine the association between specific kinetic parameters and specific PROMs may not produce clinically useful findings at the current time, but this appears to be a developing field of investigation.

### Strengths and limitations

To our knowledge, this is the first review detailing different parameters and methodological protocols applied to assess various tasks utilizing kinetic measurement systems in the ACLR patient population. In this scoping review, we followed a systematic approach, suggested by the framework of Arksey and O’Malley [7]. We searched for peer-reviewed published literature and did not restrict by publication date or language; this allowed us to identify the widest base of relevant studies on the use of kinetic measurement systems in individuals following ACLR and additionally identify the methodological gaps in the reported literature. The study team was a multidisciplinary group, including individuals with diverse expertise in research methodology, evidence synthesis, orthopaedic surgery, sport and exercise therapy, knee injury rehabilitation, kinesiology, and engineering. This reduced ambiguity and uncertainties related to study selection and reporting [93].

This review, however, has limitations. We reported only methodological considerations, and therefore cannot state what impact those methodologies had on study outcomes. Prior to comparing outcomes, we must first understand the various methodological approaches. Our intent was not to settle on a single agreement for methodological approach or outcomes post-ACLR, but rather to emphasize the need for clear and detailed methodology reporting to allow comparisons across studies to advance our understanding of the current evidence.

### Future direction

**T**he suggested methodological considerations (Table 7) in this review provide important information to support further research aimed at developing and validating a methodological reporting standard checklist for kinetic measurement systems to assess individuals following ACLR. Standardizing reporting of methodology will improve our understanding as to which kinetic measurement systems and protocols may be most clinically relevant in the ACLR population. These reporting considerations can subsequently be applied in future work to objectively inform patients and clinicians when discussing RTS decisions following ACLR. This review highlights areas for potential future systematic reviews to identify the most useful parameters, tasks, and approaches to use in individuals following ACLR.

## Conclusion

There has been substantial advancement in utilizing kinetic measurement systems in individuals post-ACLR. However, this advancement has been challenged by heterogeneity in approaches and methodological gaps in reporting. Clear and accurate reporting in clinical outcome research is important to demonstrate valid outcomes and to compare outcomes across studies. Therefore, our study suggests methodological considerations as a mechanism to assist authors in the reporting of essential items needed to improve reproducibility and subsequent quality of research in this area. Moreover, our review recommends future systematic reviews to examine the most useful kinetic parameters and approaches to follow when assessing specific functional tasks performed by individuals following ACLR. However, a systematic review to examine the association between specific kinetic parameters and specific PROMs may not produce clinically useful findings at the current time due to the scarcity and heterogeneity in the available evidence.

## Supplementary Information

**Additional file 1.** Search Strategies.

**Additional file 2.** Data Extraction Tables.

## Data Availability

All included papers and data extraction spreadsheet are available upon request.
