# Supplementary material for: Kinetic measurement system use in individuals following anterior cruciate ligament reconstruction: a scoping review of methodological approaches
Source: J Exp Orthop. 2021 Sep 26;8:81. doi: 10.1186/s40634-021-00397-0 (PMC8473525; doi:10.1186/s40634-021-00397-0)
Supplement: Supplementary file 1 — Additional file 1. Search Strategies. [file 40634_2021_397_MOESM1_ESM.docx]

**Supplementary File I - Search Strategies**

**Ovid MEDLINE(R) ALL 1946 to June 04, 2020**

Date searched: June 5, 2020

Results: 1194

1. exp anterior cruciate ligament reconstruction/

2. ((Anterior cruciate ligament or ACL) adj8 (repair or reconstruct* or surgery or post-operativ* or postoperativ*)).mp.

3. 1 or 2

4. (forceplate* or force plate* or force platform* or balance platform* or balance board* or wii balance or unstable platform or KAT-2000 or KAT2000 or platform system or biodex stability system or biodex balance system or centre of gravity or center of gravity or Neruocom balance master or Kistler or GRF or GRFs or VGRF or VGRFs or ground reaction force* or kinetic* or center of pressure).mp.

5. (Reactive strength index-modified or RSImod or vertical impulse or rate of force development or force production or jump duration or flight time or peak force* or fatigue index or reactive strength index or limb-impulse* or phase specific or knee-extensor-power or muscle-power or time curve).mp.

6. (postural stability or postural instability or postural balance or postural control or postural sway or postural impairment* or dynamic balance or dynamic stability or dynamic control or static balance or static stability or static control or standing balance or balance impairment* or stabilometric).mp.

7. (functional-test* or quiet standing or hop test or single leg hop or single leg squat or landing or (jump adj2 height) or ((Bilateral or unilateral or countermovement or squat or drop or vertical) adj4 jump*) or ((Leg or legs or limb or limbs or knee or knees or functional or strength or muscle or index or indices) adj4 (asymmetr* or symmetr*))).mp.

8. 3 and (or/4-7)

9. limit 8 to (address or autobiography or bibliography or biography or clinical trial, veterinary or clinical trials, veterinary as topic or dictionary or directory or editorial or interview or news or newspaper article or observational study, veterinary)

10. 8 not 9

**Embase 1974 to 2020 June 04 (OVID interface)**

Date searched:June 5, 2020

Results: 1268

1. anterior cruciate ligament reconstruction/

2. ((Anterior cruciate ligament or ACL) adj8 (repair or reconstruct* or surgery or post-operativ* or postoperativ*)).mp.

3. 1 or 2

4. (forceplate* or force plate* or force platform* or balance platform* or balance board* or wii balance or unstable platform or KAT-2000 or KAT2000 or platform system or biodex stability system or biodex balance system or centre of gravity or center of gravity or Neruocom balance master or Kistler or GRF or GRFs or VGRF or VGRFs or ground reaction force* or kinetic* or center of pressure).mp.

5. (Reactive strength index-modified or RSImod or vertical impulse or rate of force development or force production or jump duration or flight time or peak force* or fatigue index or reactive strength index or limb-impulse* or phase specific or knee-extensor-power or muscle-power or time curve).mp.

6. (postural stability or postural instability or postural balance or postural control or postural sway or postural impairment* or dynamic balance or dynamic stability or dynamic control or static balance or static stability or static control or standing balance or balance impairment* or stabilometric).mp.

7. (functional-test* or quiet standing or hop test or single leg hop or single leg squat or landing or (jump adj2 height) or ((Bilateral or unilateral or countermovement or squat or drop or vertical) adj4 jump*) or ((Leg or legs or limb or limbs or knee or knees or functional or strength or muscle or index or indices) adj4 (asymmetr* or symmetr*))).mp.

8. 3 and (or/4-7)

9. limit 8 to conference abstract status

10. 8 not 9

11. limit 10 to editorial

12. 10 not 11

**CINAHL Plus with Full Text (EBSCOhose interface)**

Date searched: June 5, 2020

Results: 966

S1 (MH "Anterior Cruciate Ligament Reconstruction") OR ( (Anterior cruciate ligament or ACL) N8 (repair or reconstruct* or surgery or post-operativ* or postoperativ*)) )

S2 forceplate* or force-plate* or force-platform* or balance-platform* or balance-board* or wii-balance or unstable-platform or KAT-2000 or KAT2000 or platform-system or stability-system or balance-system or centre-of-gravity or center-of-gravity or balance-master or Kistler or GRF or GRFs or VGRF or VGRFs or ground-reaction-force* or kinetic* or center-of-pressure or centre-of-pressure or Reactive-strength-index-modified or RSImod or vertical-impulse or rate-of-force-development or force-production or jump-duration or flight-time or peak-force* or fatigue-index or reactive-strength-index or limb-impulse* or phase-specific or knee-extensor-power or muscle-power or time-curve or postural-stability or postural-instability or postural-balance or postural-control or postural-sway or postural-impairment* or dynamic-balance or dynamic-stability or dynamic-control or static-balance or static-stability or static-control or standing-balance or balance-impairment* or stabilometric or functional-test* or quiet-standing or hop-test or single-leg-hop or single-leg-squat or landing or (jump N2 height) or ((Bilateral or unilateral or countermovement or squat or drop or vertical) N4 jump*) or ((Leg or legs or limb or limbs or knee or knees or functional or strength or muscle or index or indices) N4 (asymmetr* or symmetr*))

S3 S1 AND S2

S4 S3 Limiters - Publication Type: Biography, Book Review, Editorial, Obituary, Pamphlet, Pamphlet Chapter, Proceedings

S5 S3 NOT S4

**SPORTDiscus with Full Text (EBSCOhose interface)**

Date searched: June 5, 2020

Results: 846

S1 ( ( (Anterior cruciate ligament or ACL) N8 (repair or reconstruct* or surgery or post-operativ* or postoperativ*)) ) AND ( forceplate* or force-plate* or force-platform* or balance-platform* or balance-board* or wii-balance or unstable-platform or KAT-2000 or KAT2000 or platform-system or stability-system or balance-system or centre-of-gravity or center-of-gravity or balance-master or Kistler or GRF or GRFs or VGRF or VGRFs or ground-reaction-force* or kinetic* or center-of-pressure or centre-of-pressure or Reactive-strength-index-modified or RSImod or vertical-impulse or rate-of-force-development or force-production or jump-duration or flight-time or peak-force* or fatigue-index or reactive-strength-index or limb-impulse* or phase-specific or knee-extensor-power or muscle-power or time-curve or postural-stability or postural-instability or postural-balance or postural-control or postural-sway or postural-impairment* or dynamic-balance or dynamic-stability or dynamic-control or static-balance or static-stability or static-control or standing-balance or balance-impairment* or stabilometric or functional-test* or quiet-standing or hop-test or single-leg-hop or single-leg-squat or landing or (jump N2 height) or ((Bilateral or unilateral or countermovement or squat or drop or vertical) N4 jump*) or ((Leg or legs or limb or limbs or knee or knees or functional or strength or muscle or index or indices) N4 (asymmetr* or symmetr*)) )

S2 S1 Limiters - Publication Type: Audio, Audiocassette, CD-ROM, Computer Disk or Diskette, Conference Proceeding, Newspaper, Newswire, Proceeding, Trade Publication, Video, Video Recording, Videocassette, URL

S3 S1 NOT S2

**SCOPUS**

Date searched:June 5, 2020

Results: 1395

TITLE-ABS-KEY ( ( anterior-cruciate-ligament OR acl ) W/8 ( repair OR reconstruct* OR surgery OR post-operativ* OR postoperativ* ) ) AND TITLE-ABS-KEY ( forceplate* OR force-plate* OR force-platform* OR balance-platform* OR balance-board* OR wii-balance OR unstable-platform OR kat-2000 OR kat2000 OR platform-system OR stability-system OR balance-system OR centre-of-gravity OR center-of-gravity OR balance-master OR kistler OR grf OR grfs OR vgrf OR vgrfs OR ground-reaction-force* OR kinetic* OR center-of-pressure OR centre-of-pressure OR reactive-strength-index-modified OR rsimod OR vertical-impulse OR rate-of-force-development OR force-production OR jump-duration OR flight-time OR peak-force* OR fatigue-index OR reactive-strength-index OR limb-impulse* OR phase-specific OR knee-extensor-power OR muscle-power OR time-curve OR postural-stability OR postural-instability OR postural-balance OR postural-control OR postural-sway OR postural-impairment* OR dynamic-balance OR dynamic-stability OR dynamic-control OR static-balance OR static-stability OR static-control OR standing-balance OR balance-impairment* OR stabilometric OR functional-test* OR quiet-standing OR hop-test OR single-leg-hop OR single-leg-squat OR landing OR ( jump W/2 height ) OR ( ( bilateral OR unilateral OR countermovement OR squat OR drop OR vertical ) W/4 jump* ) OR ( ( leg OR legs OR limb OR limbs OR knee OR knees OR functional OR strength OR muscle OR index OR indices ) W/4 ( asymmetr* OR symmetr* ) ) ) AND ( EXCLUDE ( DOCTYPE , "cp" ) OR EXCLUDE ( DOCTYPE , "no" ) OR EXCLUDE ( DOCTYPE , "ed" ) )

**Web of Science** Indexes=SCI-EXPANDED, SSCI, A&HCI, ESCI

Date searched: June 5, 2020

Results: 1259

TS=( ( anterior-cruciate-ligament OR acl ) NEAR/8 ( repair OR reconstruct* OR surgery OR post-operativ* OR postoperativ* ) ) AND TS=( ( anterior-cruciate-ligament OR acl ) NEAR/8 ( repair OR reconstruct* OR surgery OR post-operativ* OR postoperativ* ) ) AND DOCUMENT TYPES: (Article OR Book OR Book Chapter OR Correction OR Data Paper OR Letter OR Note OR Retracted Publication OR Retraction OR Review)

Indexes=SCI-EXPANDED, SSCI, A&HCI, ESCI Timespan=All years

**Dissertations and Theses Global(Proquest interface)**

Date searched:June 5, 2020

Results: 118

noft(((anterior-cruciate-ligament OR acl) NEAR/8 (repair OR reconstruct* OR surgery OR post-operativ* OR postoperativ*))) AND (forceplate* or force-plate* or force-platform* or balance-platform* or balance-board* or wii-balance or unstable-platform or KAT-2000 or KAT2000 or platform-system or stability-system or balance-system)
