# Supplementary material for: Kinetic measurement system use in individuals following anterior cruciate ligament reconstruction: a scoping review of methodological approaches
Source: J Exp Orthop. 2021 Sep 26;8:81. doi: 10.1186/s40634-021-00397-0 (PMC8473525; doi:10.1186/s40634-021-00397-0)
Supplement: Supplementary file 2 — Additional file 2. Data Extraction Tables. [file 40634_2021_397_MOESM2_ESM.docx]

**Supplementary File II- Data Extraction Tables**

Table 1 Data Extraction Table for Studies Assessing Landing/Jumping

| Study Characteristic  (author, year, design, country, language) | **Sample Characteristic**  **(Physical Activity (PA), Activity level, time since surgery (mean**$\boldsymbol{\pm}$**SD), sample size by sex and age (mean**$\boldsymbol{\pm}$**SD)** | **Study Objectives** | **Parameters** | | **Sampling Frequency** | **Number of Repetitions** | | - **Testing Condition or Challenges** - **Protocol Summery** | |
| --- | --- | --- | --- | --- | --- | --- | --- | --- | --- |
| Studies used Force Plates (n=56) | | | | | | | | | |
| Bell et al, 2014 [26] Cross Sectional USA  In English | PA: Soccer (8), flag football (3), basketball (2), gymnastics (2), softball (1), long jump (1), volleyball (1), handball (1) Level: N/R Time Since ACLR: 26.3$\pm$12.6 months ACLR: (m=0), (f=19); Age: 19.6$\pm$1.3 y/o Control: N/A ACLD: (m=0), (f=1); Age: N/R y/o | To examine anterior knee laxity and 3- dimensional hip and knee kinematics and kinetics across the menstrual cycle in a population of women with previous unilateral, noncontact ACL injuries | GRF | 1440 Hz | | 5 | Participants completed 5 trials of a jump-landing task on a box that was 30 cm high and positioned at 50% of their heights from the edge of a force plate. They jumped forward and landed on both feet with the test limb on the force plate. Immediately after landing, participants jumped as high as possible | |  |
| Birchmeier et al, 2019 [27] Cross Sectional USA  In English | PA: N/R Level: N/R Time Since ACLR: 37.6$\pm$23.7 months ACLR: (m=19), (f=33); Age: 22.94$\pm$5.0 y/o Control: N/A ACLD: N/A | To assess the association of isometric knee extension strength characteristics and plyometric characteristics measured during a single-leg drop landing(amortization time and RSI), with single-leg hop performance in individuals with a history of ACLR | Ground contact time | 1200 Hz | | 3 | Shoes on  Participants stood on a 30-cm box placed 40 cm from the middle of the embedded force plate. They were instructed to jump to a target in the middle of the force place, land on a single leg, then immediately perform a maximal vertical jump off the same leg | |  |
| **Blache et al, 2017 [79] Cross Sectional France  In English | PA: Soccer, Basketball, Handball Level: 2-3 times/week (level N/R) Time Since ACLR: 7.3 (range 5-9) months ACLR: (m=12), (f=0); Age: 23.9$\pm$5.8 y/o Control: (m=12), (f=0); Age: 25.5$\pm$4.5 y/o ACLD: N/A | to evaluate the inter-joint coordination asymmetry between IL and NIL in patients after ACL-R during single-leg vertical jumping in comparison to a healthy population | Vertical jump height was obtained from vGRF | 1000 Hz | | 3 | 15-min warm-up, including single-leg squat jumps  Barefoot, on 1 leg, arms akimbo  Three single-leg squat jumps with the right and left legs on force plate. Participants asked to jump as high as possible without any countermovement | |  |
| Chang et al, 2018 [28] Quasi Experimental South Korea  In English | PA: N/R Level: N/R Time Since ACLR: ACLR: 35.2$\pm$13.2 months ACLR: (m=0), (f=18); Age: ACLR: 19.9$\pm1.2$ Control: (m=0), (f=12); Age: 21.0$\pm$2.6 y/o ACLD: N/A | To compare the landing biomechanics of ACLR females who pass or fail an FTB to the matched-limb landing biomechanics of healthy females before and after completion of a sustained exercise protocol | Peak vGRF | 1560 Hz | | 3 | 5-min submaximal warm-up on a stationary bike.  For double leg jump landing, participants stood atop a 30-cm high box placed 50% of their height from the front edge of the force plate. They were instructed to jump forward off the box and land on the force plates with both feet then immediately jump vertically. For single leg jump landing, participants stood on the floor behind a line marked 50% of their height from the front edge of the force plate. Then, they were instructed to jump over a 17-cm high hurdle placed 25% of their height from the force plate, land on 1 foot (testing foot), and then cut as quickly as possible to the other direction of the landing foot (e.g. right foot landing then cut to the left side). | |  |
| Chang et al, 2020 [29] Cross Sectional South Korea  In English | PA: N/R Level: N/R Time Since ACLR: 35.2$\pm$18.4 months ACLR: (m=0), (f=18); Age: 19.9$\pm$1.2 y/o Control: (m=0), (f=12); Age: 21.0$\pm$2.6 y/o ACLD: N/A | To compare the knee joint landing and cutting biomechanics asymmetry of ACLR females that pass and fail an FTB with healthy females before and after the completion of a sustained exercise protocol. It was hypothesized that there would be no differences in landing and cutting mechanics asymmetry between ACLR females that pass an FTB (ACLR-pass) and healthy females; but that ACLR females that fail an FTB (ACLR-fail) would exhibit different landing and cutting mechanics asymmetry compared to ACLR-pass and healthy females. | Peak vGRF vGRF loading rate | 1560 Hz | | 3 | For double leg jump landing, participants stood atop a 30-cm high box placed 50% of their height from the front edge of the force plate. They were instructed to jump forward off the box and land on the force plates with both feet then immediately jump vertically. For single leg jump landing, participants stood on the floor behind a line marked 50% of their height from the front edge of the force plate. Then, they were instructed to jump over a 17-cm high hurdle placed 25% of their height from the force plate, land on 1 foot (testing foot), and then cut as quickly as possible to the other direction of the landing foot (e.g. right foot landing then cut to the left side). | |  |
| Dashti Rostami et al, 2020 [30] Cross Sectional Iran  In English | PA: N/R Level: exercise for at least 3 sessions per week for 30 minutes per session Time Since ACLR: 26.55$\pm$4.31 months ACLR: (m=20), (f=0); Age: 26.77$\pm$3.75 y/o Control: N/A ACLD: N/A | To evaluate relationships between lower extremity muscle activity (quadriceps, hamstrings, gastrocnemius, and quadriceps to hamstring [Q: H] co-activation ratio) and peak vertical and posterior ground reaction forces (vGRF and pGRF) during a single leg drop-landing task. | Peak vertical GRF Peak posterior GRF | 2000 Hz | | 3 | Hand on the hips Contralateral knee in flexion, and tibia is not touching the other leg.  Following a verbal cue, participants dropped off the platform and landed upon the force plate. Participants performed 3 practice trials followed by 3 test trials. Trials were considered valid if participants successfully landed with the entire foot on the force plate and maintained balance upon the injured limb only. | |  |
| **deFontenay et al, 2014 [80] Cross Sectional France  In English | PA: N/R Level: N/R Time Since ACLR: 7.3$\pm$1.1 months ACLR: (m=11), (f=0); Age: 23.3$\pm$3.8 y/o Control: N/A ACLD: N/A | To highlight the alterations observed in the IL during the performance of a dynamic movement after ACL reconstruction. | Push off phase duration | 1000 Hz | | 3 | Warm-up and jump-training sessions to become familiar with the task.  Countermovements were not allowed. Hands stay on hips.  Each participant performed 6 maximal single-legged squat jumps: 3 jumps on the injured limb and 3 jumps on the uninjured limb in randomized order. The initial position was the preferred position they chose, and they were instructed to jump as high as possible without downward movement. | |  |
| Decker et al, 2002 [31] Cross Sectional USA  In English | PA: Athlete Level: Recreational Time Since ACLR: More than 1 year (mean$\pm$SD: N/R) ACLR: (n=11), (Sex: N/R); Age: 27.3 (SD: N/R) y/o Control: (n=11), (Sex: N/R); Age: 26.9 (SD: N/R) y/o ACLD: N/A | To evaluate and compare the kinetic and kinematic landing performances of healthy and hamstring ACLR individuals. | vGRF | 1200 Hz | | 8 | Warm-up on the treadmill for 5 minutes  Hands folded on chest  The landing task consisted of stepping off a 60-cm box onto a landing platform. The subjects were instructed to step off the box, without jumping up or stepping down, and to land as naturally as possible with both feet on the landing platform. One foot landed upon a force plate, and the other landed next to the force plate on the landing platform. | |  |
| Elias et al, 2015 [32] Cross Sectional USA  In English | PA: Basketball, skiing, soccer, volleyball, football and other Level: Recreational Time Since ACLR: 23.6$\pm$14 months ACLR: (m=14), (f=20); Age: 21.9$\pm$4.5 y/o Control: N/A ACLD: N/A | To examine training-induced changes in quadriceps and hamstring muscle activity following instruction for what has been reported as a preferred strategy for impact attenuation during a single-leg landing task in persons who have undergone ACLR. | vGRF | 1200 Hz | | 5 | Warm-up on treadmill for 5 minutes  Shoes on  Before instructions: Subjects stood approximately 10 cm from the edge of a 20-cm box with their hands on their hips, and were instructed to gain their balance on a single leg before hopping forward off the box with their eyes looking forward.   After instructions: To land as softly and quietly as possible by hitting toes first and bending their knees during landing. Participants were instructed to keep their chest over their knees and their knees over their toes during landing. | |  |
| Flanagan et al, 2008 [33] Cross Sectional Ireland  In English | PA: Field sports such as rugby and soccer Level: N/R Time Since ACLR: 27.0$\pm$14.5 months ACLR: (m=8), (f=2); Age: 23.8$\pm$6 y/o Control: (m=8), (f=2); Age: 23.3$\pm$3.1 y/o ACLD: N/A | To determine whether rehabilitated ACL-R individuals were left with residual deficits in performance after their rehabilitation programs. | Peak ground reaction force Flight time  Contact time | 1000 | | 3 | Setting on a 30 degrees inclined sledge.  Shoes on Movement is only in the sagittal plane  Participants were asked to perform squat jumps, countermovement Jumps,  drop Jumps and rebound Jumps | |  |
| Ford et al, 2016 [34] Cross Sectional USA  In English | PA: N/R Level: N/R Time Since ACLR: 8.3$\pm$2.5 months ACLR: (m=37), (f=64); Age: 16.7$\pm$3.0 y/o Control: (m=15), (f=42); Age: 17.2$\pm$2.5 y/o ACLD: N/A | To objectively classify the preferred landing leg during a bipedal landing task in athletes previously injured and uninjured  To determine if limb asymmetries during single leg hops would be observed within ACLR and control groups based on group allocation. | The absolute time difference between the initial contacts for each leg was calculated and compared between ACLR and control groups. | 1200 Hz | | 3 | Both feet at the same time  Drop down of a box on a force plate and jump vertically. | |  |
| Frank et al, 2014 [35] Quasi Experimental USA  In English | PA: N/R Level: N/R Time Since ACLR: 35.0$\pm$16.9 months ACLR: (m=0), (f=14); Age: 19.6$\pm$1.5 y/o Control: N/A ACLD: N/A | To investigate the effects of fatigue on lower extremity biomechanics and postural control in active females with ACLR | vGRF | 1400 Hz | | 5 Double leg jump landings 3 single leg balance | 5-min of a light, self-directed stationary bike warm up followed by 5-min of stretching before testing  Double-Leg Jump Landing Jump of a 30-cm box placed at a distance equal to one half the participant’s height from the leading edge of the force plate. On landing, participants were instructed to jump up as high as possible.   Single-Leg Balance Eyes closed while standing unshod atop the center of a force plate. Participants were instructed to place their hands on their hips for the duration of the balance task. Each participant attempted to balance on their ACLR limb for 20 seconds while center-of-pressure (CoP) data were recorded. | |  |
| Furlanetto et al, 2016 [36] Cross sectional Brazil  In English | PA: N/R Level: N/R Time Since ACLR: 6 months ACLR: (n=20), (Sex: N/R); Age: 29.2$\pm$8.1 y/o Control: (n=20), (Sex: N/R); Age: 27.8$\pm$4.0 y/o ACLD: N/A | Evaluate and compare proprioception, postural control and knee function in subjects with and without unilateral ACL reconstruction | Postural control: Amplitude of CoP (anterior-posterior and medial-lateral directions) Step up and down: first peak vGRF, load application rate (LAR) | 2000 Hz | | 3 trials each (PC), 5 trials each (SUD) | Postural control (PC): Right and left uni-podal support lasting for 30 sec each. The individual was asked to remain still in the indicated position with his/her hands on the anterio-superior iliac crests (ASIC), silently, gaze fixed on a target, located 1m from the eye level of each participant.   Step up and down (SUD): 30 cm high wooden box placed on 1st force plate. The test started off the force platform with the patient in static position, legs together and hands on anterior superior iliac spine. The individual climbed the step with 1 of his/her lower limbs and descended it by stepping on 2nd force plate in a continuous single movement. | |  |
| Gokeler et al, 2010 [37] Cross Sectional The Netherlands  In English | PA: Level 1-2 sports (jumping, pivoting, hard-cutting, lateral motion) Level: N/R Time Since ACLR: 6.75$\pm$0.38 months ACLR: (m=6), (f=3); Age: 28.4$\pm$9.7 y/o Control: (m=8), (f=3); Age: 26.3$\pm$5.5 y/o ACLD: N/A | To assess the bilateral lower limb joint kinematics and kinetics and onset time of EMG activity during the single leg hop test in ACL-reconstructed patients during the single leg hop for distance. These data will be compared with a control group. | Horizontal and vertical GRF (normalized to body weight) | 750 Hz | | 10 | Single leg, wearing own shoes  Force plate was placed at maximum hop distance for that subject, and the subject was instructed hop with 1 limb and land onto the center of the force plate. | |  |
| Gokeler et al, 2016 [38] Cross Sectional The Netherlands  In English | PA: N/R Level: Hours sport per week (prior to injury) = 6.8$\pm$3.8 Time Since ACLR: 8.9$\pm$2.3 months ACLR: (m=10), (f=10); Age: 23.5$\pm$4.3 y/o Control: (m=10), (f=10); Age: 22.7$\pm$2.3 y/o ACLD: N/A | To evaluate the influence of immersion in virtual reality on movement patterns in patients after ACLR while performing a step-down task. | GRF normalized to body weight | N/R | | 8 | Shoes on, arms across chest. Virtual reality and non-virtual reality conditions  Step-down from a 20-cm-high box onto 2 force plates of 40 × 60 cm that were embedded in the floor in front of the box. Virtual reality environment condition customized with CAREN | |  |
| Grooms et al, 2018 [39] Cross Sectional USA  In English | PA: N/R Level: N/R Time Since ACLR: 36.18$\pm$26.50 months ACLR: (m=7), (f=8); Age: 21.4$\pm$2.6 y/o Control: (m=7), (f=8); Age: 23.2$\pm$3.5 y/o ACLD: N/A | To investigate the effects of stroboscopic visual-feedback disruption (SVFD) on drop vertical-jump (DVJ) landing mechanics and determine the influence of ACLR history on the effect of SVFD on neuromuscular control | Peak GRF (as % body mass) | N/R | | 3 | 3 conditions: full vision, low SVFD, and high SVFD  For the DVJ assessment, participants fell forward from a 30-cm box, immediately performed a vertical jump, raised both upper extremities, and hit a target set at 90% of their maximal jump height. The SPARQ Vapor Strobe goggles (Nike, Inc, Beaverton, OR) imposed the SVFD condition | |  |
| Holsgaard-Larsen et al, 2014 [40] Cross Sectional Denmark  In English | PA: N/R Level: N/R Time Since ACLR: 26.5$\pm$6.6 months ACLR: (m=23), (f=0); Age: 27.2$\pm$7.5 y/o Control: (m=25), (f=0); Age: 27.2$\pm$5.4 y/o ACLD: N/A | To conduct concurrent assessments of lower limb loading patterns during bilateral and unilateral vertical jumping, isolated mechanical muscle function and functional outcome in ACL reconstructed patients and to test the hypothesis that patients demonstrate greater between-limb asymmetry in these parameters compared with age-matched controls. Additionally, to more closely investigate the origin of between-limb asymmetry by means of kinematic/kinetic movement analysis. | vGRF | 1000 Hz | | 3 | A standardized warm-up consisting of two times 10 toe rises, 10 bilateral squats, 10 unilateral squats (for each leg) and two to three submaximal vertical jumps (countermovement jumps: CMJ).  Double leg stance, hands on hips, barefoot  Starting from a full erect standing position, the subjects were instructed to perform a fast downward movement (to about 90° knee flexion) immediately followed by a fast upward movement, and to jump as high as possible. | |  |
| Kilic et al, 2018 [41] Cross Sectional Turkey  In English | PA: Soccer Level: Amature Time Since ACLR: (Range 6-15) months ACLR: (m=11), (f=0); Age: 23.1$\pm$3.62 y/o Control: (m=9), (f=0); Age: 22.2$\pm$2.48 y/o ACLD: N/A | To determine how ground reaction forces, moments and knee flexion angles differ between healthy controls and reconstructed subjects during single leg landing phases. | Phase vGRF | 1000 Hz | | 3 | 5-min warm-up  Shoes on  Drop jump from a custom-made takeoff platform from 20cm vertical height that was placed next to the edge of a force plate. For each landing task all participants began with a standard takeoff position by standing on a takeoff platform with hands placed on the hips, legs shoulder width apart, and the toes of both feet aligned with the edge of the takeoff platform. Participants were then instructed to stand on their dominant leg, drop off, and land as naturally as possible with their dominant foot only centered on the force plate and jump vertically as soon as possible. The participants were asked to keep their hands on their hips when landing to reduce any variability from swinging arms. | |  |
| King et al, 2018 [42] Cross Sectional  Ireland  In English | PA: Multidirectional field sport Level: N/R Time Since ACLR: 8.8$\pm$0.7 months ACLR: (m=156), (f=0); Age: 24.8$\pm$4.8 y/o Control: N/A ACLD: N/A | To identify biomechanical and performance variable differences between ACLR and non-ACLR limbs 9 months after surgery across a number of jump tests | GRF (vertical, posterior) | 1000 Hz | | 3 trials of each limb and jump | A standardized warm-up: a 2-min jog, 5 bodyweight squats, 2 submaximal and 3 maximal double leg countermovement jumps.  Double legs drop jump (DLDJ). Single leg drop jump (SLDJ), single leg hop for distance (SLHD) and hurdle hop (HH) tests. Wearing own footwear, hands on hips  DLDJ (30cm step), SLDJ (20cm step): subject was asked to roll from the step and upon hitting the ground, to jump as high as possible while spending as little time as possible on the force plate. For the DLDJ the subject started with their feet approximately hip width apart and landed with 1 foot on each of the force plates. HH (15cm hurdle): starting by standing on the leg to be tested then jumping over the hurdle towards the contralateral side and then rebound over the hurdle again to the start position | |  |
| King et al, 2019 [85] Cross Sectional  Ireland  In English | PA: Multidirectional field sports Level: N/R Time Since ACLR: 9.4$\pm$0.7 months ACLR: (m=156), (f=0); Age: 24.8$\pm$4.2 y/o Control: (m=62), (f=0); Age: 24.7$\pm$3.9 y/o ACLD: N/A | To identify differences in asymmetry of biomechanical and performance variables during jump and change of direction testing between athletes who were 9 months post-ACLR and a matched healthy cohort | Ground reaction force (N/kg) in vertical, medial and posterior directions | 1000 Hz | | 3 | A standardized warm-up: a 2-min jog, 5 bodyweight squats, 2 submaximal and 3 maximal double leg counter movement jumps.  Wearing own athletic footwear  The testing protocol included the DLDJ from 30 cm, the SLDJ from 20 cm, the SLHD, and 90° planned and unplanned change of direction | |  |
| *Krafft et al, 2017 [82] Cohort Germany  In English | PA: Athletes Level: Recreational Time Since ACLR: 6 months ACLR: (m=N/R), (f=); Age: 32.0$\pm$13.3 y/o Control: (m=N/R), (f=N/R); Age: 33.3$\pm$13.4 y/o ACLD: N/A | To examine the functional state of ACL reconstructed subjects comprehensively by the combination of self-evaluating questionnaires, functional clinical as well as static and dynamic functional performance testing (FPTs) and in comparison to matched healthy control subjects | Jumping height (absolute value) Acceleration impulse during take-off (LSI) Deceleration impulse during landing (LSI) | 1000 Hz | | 3 | Double leg support  Double leg support Subjects performed 3 countermovement jumps (CMJs) akimbo | |  |
| Królikowska et al, 2018 [44] Case Control Poland  In English | PA: Different sports Level: Recreational Time Since ACLR: 25.8$\pm$10.3 months ACLR: (m=38), (f=0); Age: 29.7$\pm$8.6 y/o Control: (m=38), (f=0); Age: 26.3$\pm$4.1 y/o ACLD: N/A | To investigate whether double-leg and single-leg vertical hop landing between-limb symmetry in males, an average of 2 years after ACLR, is associated with the duration of postoperative physiotherapy supervision. | Peak vGRF vGRF normalized to the body mass (BM) The vGRF BM limb symmetry index (LSI) | N/R | | 10 | Sport outfit and sport shoes. While single hopping, the contralateral knee was flexed 90◦  The participant placed his right foot on the middle of the right force plate and left foot on the middle of the left plate. Then, he performed 10 continuous double-leg vertical hops. Next, the vGRF values during the single-leg vertical hops were measured, starting with the uninvolved leg in ACL-reconstructed patients and with the dominant limb in the control group. The examined participant placed the foot of the studied leg in the middle of the force plate and performed 10 continuous single-leg vertical hops | |  |
| Królikowska et al, 2018 [45] Cross Sectional Poland  In English/Polish | PA: Different sports Level: Recreational Time Since ACLR: 28.1$\pm$11.0 months ACLR: (m=15), (f=0); Age: 29.8$\pm$4.5 y/o Control: (m=15), (f=0); Age: 23.1$\pm$1.7 y/o ACLD: N/A | To assess the lower limb loading asymmetry in the landing phase of hops in males, 2 years after ACLR and the assessment of the supervised postoperative physiotherapy during the period shorter than 6 months. | vGRF normalized to the body mass The vGRF BM limb symmetry index (LSI) | N/R | | 10-Jun | Warm-up on a cycle ergometer.  Sport outfit and sport shoes. While single hopping, the contralateral knee was flexed 90◦  The participant placed his right foot on the middle of the right force plate and left foot on the middle of the left plate. Then, he performed 6-10 continuous double-leg vertical hops. Next, the vGRF values during the single-leg vertical hops were measured, starting with the uninvolved leg in ACL-reconstructed patients and with the dominant limb in the control group. The examined participant placed the foot of the studied leg in the middle of the force plate and performed continuous single-leg vertical hops | |  |
| Krolikowska et al, 2018 [46] Case Control Poland  In English | PA: Different sports Level: Recreational Time Since ACLR: 7.5$\pm1.7$ months ACLR: (m=35), (f=0); Age: 25.8$\pm$5.2 y/o Control: (m=20), (f=0); Age: 22.5$\pm$1.8 y/o ACLD: N/A | To investigate the vertical jump landing limb symmetry at 7 months after ACL reconstruction between a group of patients receiving a longer supervised physiotherapeutic procedure and a group of patients who followed a shorter supervised physiotherapy. | Body mass peak vertical ground reaction force Limb symmetry index (LSI) | N/R | | 10-Jun | Warm-up on cycle ergometer.  Sport outfit and sport shoes. While single hopping, the contralateral knee was flexed 90◦  Each jump was performed in the upright position. The protocol did not allow for countermovement, and arm movement during the jumps was restricted. First, VGRF values during two-legged vertical jumps landing were measured. At the beginning of the measurement the participant placed his right foot on the middle of the right force plate and left foot on the middle of the left plate. The participant then performed 6 - 10 continuous 2-legged vertical jumps. Second, VGRF values during 1 legged vertical jumps landing were measured, starting with the uninvolved leg in Groups I and II and with the right leg in Group III. The participant placed the foot of the studied leg in the middle of the force plate and performed 6 - 10 continuous 1-legged jumps. The second leg was flexed 90 degrees at the knee joint. Measurement was then performed for the second leg in the same way. | |  |
| Kuster et al, 1999 [47] Quasi Experimental Swizerland  In English | PA: N/R Level: N/R Time Since ACLR: 33.6$\pm$7.2 months ACLR: (m=24), (f=12); Age: 31.7$\pm$9.9 y/o Control: N/A ACLD: N/A | “The present study used the one-legged stance test to evaluate the enhancement of muscle control and coordination afforded by the use of an elastic compression sleeve after ACL reconstruction. It also included a one-legged drop jump to further stress the balance control system.” | Peak impact loading for the landing phase.   Force-time integrals   Path length (PL),   Root mean square error of the force components   CoP | 100 Hz | | 3 | Participants were required to perform a standing drop jump from a 10-cm-high platform onto a force plate landing on 1 leg and thereafter maintain a one-legged balance for 25 s. This task was repeated on the previously injured leg 3 times without and 3 times with an elastic compression sleeve. | |  |
| Labanca et al, 2016 [48] Cohort Italy  In English | PA: Sports Level: Competitive level Time Since ACLR: 6 months ACLR: (m=53), (f=5); Age: 22.0$\pm$6.0 y/o Control: N/A ACLD: N/A | To investigate the relationship between asymmetrical lower extremities loading 1 mo. after ACL reconstruction measured by means of STS movement and asymmetrical lower extremity loading 6 mo. after surgery measured by means of CMJ. | GRF LSI | 100 Hz | | 3 | Subjects were asked to stand in an upright position with shoes on and hands on their hips. They were then asked to quickly squat with knees flexes to approximately 90 degrees and then jump immediately as high as possible without pausing. A total of 3 CMJ trials with 1-min rest in between were performed for each session. | |  |
| Labanca et al, 2018 [49] Experimental Italy  In English | PA: Sports Level: Competitive Time Since ACLR: 6 months ACLR: (m=63), (f=0); Age: 23.2$\pm$4.6  Control: N/A ACLD: N/A | To investigate the effectiveness of introducing an additional rehabilitation exercise based on NMES of the quadriceps muscle superimposed on voluntary sit-to-stand-to-sit exercises (STSTS) during the early phase of rehabilitation after ACL reconstruction compared with a traditional rehabilitation protocol alone or a traditional protocol associated with STSTS exercises without NMES. | GRF LSI | 100 Hz | | 3 | Shoes on  Patients were asked to stand in an upright position and maintain their hands on their hips during performance of the whole movement. They were asked to quickly squat with knees flexed to approximately 90 degrees and then jump immediately as high as possible without pausing. | |  |
| Lam et al, 2011 [50] Cross Sectional China  In English | PA: N/R Level: N/R Time Since ACLR: 10.3$\pm$3.9 months ACLR: (m=10), (f=0); Age: 27.2$\pm$4.7 y/o Control: N/A ACLD: N/A | To prospectively investigate the range of tibial rotation of ACLD and ACLR knees during a high-demand task | Vertical ground reaction force (only presented in a graph as an example, but not quantified) | 1080 Hz | | N/R | Pivot direction (testing left and right leg)  Participants jumped off a platform, 40cm in height and 10 cm behind the force plate, and landed with both feet on the ground, with only the testing foot on the force plate. After the foot contact, they pivoted 90° to the lateral side of the testing leg, which acted as the core leg during pivoting. They were then instructed to run away with their maximum effort for 3 steps after completing the pivoting movement | |  |
| Markstrom et al, 2020 [51] Cross Sectional Sweden  In English | PA: Athletes Level: Moderate to high (Tegner 7$.0\pm$2.0) Time Since ACLR: Median (IQR): 16.0 (35.2) months ACLR: (m=8), (f=24); Age: 24.1$\pm$4.5 y/o Control: (m=8), (f=24); Age: 22.9$\pm$3.1 y/o ACLD: N/A | To investigate landing control after ACLR with regard to dynamic knee robustness and whole body movement strategies during sports-mimicking side hops, and to evaluate functional performance of hop tests and knee strength. | Peak vGRF | 1200 Hz | | 05-Mar | Barefoot, holding a short rope (25 cm, with knots at each end) with both hands behind their back.  Participants hopped on 1 leg to the side (laterally with respect to the hopping leg) over a distance of 25% of body height, followed by an immediate rebound back to the starting position for the same leg.   Trials were deemed successful if the following criteria were fulfilled: a minimum 3-second single-leg stance after landing without releasing the rope, no contact of the contralateral foot with the floor, and no moving of the ipsilateral foot to maintain balance.  They alternated between legs every trial to reduce fatigue. Participants had ~5 seconds of rest between trials and ~5 minutes of rest between tests. The biomechanical analyses focused on the landing phase after the lateral hop of the SRSH, defined from initial contact (IC; vertical force .20 N) until peak knee flexion | |  |
| Markstrom et al, 2018 [52] Test-retest design Sweden  In English | PA: Athletes and non athletes Level: Moderate (Tegner score: 6.5$\pm$5) Time Since ACLR: Median (IQR): 19.0 (122.0) months ACLR: (m=8), (f=22); Age: 24.5$\pm$4.4 y/o Control: (m=8), (f=22); Age: 22.5$\pm$3.1 y/o ACLD: N/A | To evaluate within-session reliability and agreement for trunk, hip and knee angles and moments for ACLR persons in end phase of or post-rehabilitation and healthy-knee controls during SRSH landings. Further, we aimed to evaluate test-retest reliability and agreement for controls. Finally, we assessed reliability and agreement of Time to Stabilization (TTS), another measure used to evaluate knee function in ACL-injured persons | Time to Stabilization (TTS) | 1200 Hz | | 10 | Barefoot, holding a short rope (25 cm, with knots at each end) with both hands behind their back  Participants stood on 1 leg on the side of 1 tape and were instructed to laterally hop over and land on the other side of the other tape, and to as fast as possible rebound back to the starting position. They were told to control the 2nd Landing and achieve a stable posture as quickly as possible and keeping the foot of the landing leg still on the floor.   Trials were deemed successful provided that: the participant accomplished 3 s of single leg stance after landing without letting go of the rope, did not put the contralateral foot on the floor, and did not make significant adjustments with the ipsilateral foot in order to maintain balance. Participants alternated between legs every trial to avoid fatigue, starting on the non-affected leg for ACLR and dominant leg (defined as the self-preferred leg for kicking a ball) for CTRL. The time between each trial was approximately 5s. | |  |
| Melińska et al, 2015 [53] Cross Sectional Poland  In English | PA: N/R Level: N/R Time Since ACLR: 8 months ACLR: (m=6), (f=0); Age: 26.2$\pm$2.3 y/o Control: (m=22), (f=0); Age: 25.1$\pm$4.3 y/o ACLD: N/A | To evaluate balance during the landing phase in a control group and patients after anterior cruciate ligament reconstruction (ACLR) | Horizontal components ratio of ground reaction force, sum and average of fluctuations of eCoG (estimated body's mass center of gravity) | N/R | | 1 | Warm-up involved trotting (3-4 minutes), 5-7 jumps, and (after a 20s interval) 4-5 squats.  Jump-down performed from 3 step heights (0.1, 0.2, and 0.3 m). Participants jumped down and were instructed to land on toes and metatarsus on both limbs (1 limb per platform) | |  |
| Miranda et al, 2013 [54] Cross Sectional USA  In English | PA: N/R Level: Recreational Time Since ACLR: 60 months ACLR: (m=4), (f=6); Age: 26.96$\pm5.3$ y/o Control: (m=5), (f=5); Age: 25.20$\pm5.2$y/o ACLD: N/A | To compare force plate kinetic data and knee kinematic measurements from male and female ACLINT and ACLREC recreational athletes during a jump-cut maneuver in hopes that the differences would point to plausible risk factors for injury | Peak GRF (magnitude of body weight), peak vertical GRF time, peak vertical GRF magnitude | 5000 Hz | | 10 | Subjects stood 1 m from force plate with knees bent approx. 45 degrees. Upon hearing "go" prompt, subject jumped forward to landing target on force plate, and a visual directional prompt cued subject to cut left or right after landing on the target with 1 leg. Upon landing, subjects performed a side step cut and then jogged past the respective angled targets. | |  |
| *Moya-Angeler et al, 2017 [83] Cohort USA  In English | PA: N/R Level: N/R Time Since ACLR: 6 and 12 months ACLR: (m=74), (f=0); Age: 34.0$\pm$9.0 y/o Control: N/A ACLD: N/A | To evaluate the functional status prior to and at different times after ACLR, and to analyze the changes in the kinetic patterns of the involved and uninvolved limb lower during gait, sprint and 3 hop tests | Drop vertical jump: fallen maximum vertical force (MVF), impulse MVF | N/R | | 3 | All activities performed on force plates.  Gait: 5-m walkway with force plates embedded, walk at self-selected comfortable pace.   Sprint: patient started standing on both platforms, instructed to sprint as fast as possible for 5 s  Single-leg hop: stand on 1 leg, hop as far forward as possible  Drop vertical jump: dropped off 30 cm box and performed maximal jump after landing  Vertical hop test: begin standing on both platforms, hop using arms as countermovement | |  |
| Nagelli et al, 2019 [55] Cohort USA  In English | PA: N/R Level: N/R Time Since ACLR: 7.7$\pm$3.7 months  ACLR: (m=8), (f=10); Age: 19.4$\pm$7.2 y/o Control: (m=4), (f=6); Age: 16.4$\pm$3.6 y/o ACLD: N/A | The primary purpose of the study is to determine whether a neuromuscular training (NMT) program can change single-leg landing knee biomechanics within the group of athletes with ACLR.  The secondary purpose of this study was to compare the post-training single- leg landing knee mechanics between the ACLR cohort and an uninjured, control cohort. | Vertical GRF (N/kg) | 1200 Hz | | 5 | Single-leg drop (SLD) landings of both limbs off of a 30.5- cm plyometric box onto embedded force plates | |  |
| Nagelli et al, 2018 [56] Cohort USA  In English | PA: N/R Level: N/R Time Since ACLR: 7.7 $\pm$3.7 months ACLR: (m=8), (f=10); Age: 19.4$\pm$7.2 y/o Control: (m=4), (f=6); Age: 16.0$\pm$3.7 y/o ACLD: N/A | To quantify the effects of a neuromuscular training (NMT) training program on hip biomechanics and neuro- muscular control in an ACLR cohort. Second, this study sought to frame post training hip biomechanics of an ACLR cohort with reference to the same measures for a group of uninjured control athletes who also participated in the NMT program. | Peak vGRF | 1200 Hz | | 5 | The athletes performed 5 successful drop vertical jumps (DVJs) - a bilateral drop-landing task from a 31-cm-tall box, followed by an immediate maximum effort vertical jump | |  |
| Niederer et al, 2020 [57] Cross Sectional Germany  In English | PA: N/R Level: N/R Time Since ACLR: 39$\pm$12 months ACLR: (m=14), (f=13); Age: 29.7$\pm$3.1 y/o Control: N/A ACLD: N/A | 1) to delineate a potential deficit in the ability to perform unanticipated jump-landing maneuvers, 2) to develop a standardized and reproducible assessment of unanticipated single-leg jump-landing ability and quality, and 3) to assess the duration of such a (potential) unanticipated landing impairment after ACL reconstruction and RTS. | Time to stabilization (calculated over body weight and over the normal force), peak vGRF, path length of the center of pressure | 50 Hz | | 3 | Warm-up included 30 jumping jacks.  Participants performed (bilateral take- off) counter-movement jumps with single leg landings. At takeoff, the participants received visual information indicating the landing leg. A left or right footprint located on the left or right side of a vertical line appeared on a laptop screen placed in the line of sight. Leg side was chosen randomly using a complete randomization. | |  |
| Niemeyer et al, 2019 [58] Cross Sectional Germany  In English | PA: Football (2), basketball (1), tennis (2), handball (2), kickboxing (1), skiing (1), dancing (1), triathalon (1) Level: N/R Time Since ACLR: 12.7$\pm$3.5 months ACLR: (m=5), (f=6); Age: 35.6$\pm$12 y/o Control: N/A ACLD: N/A | 1) Reproduce previous findings of a side-to-side asymmetry in unanticipated jump- landing outcomes after ACL-reconstruction  2) Reveal if the values themselves and/or the LSI of such landings provide more relevant in- formation,  3) Determine if the jump-landing assessment, in comparison to well-established RTS jumping criteria, provides unique information | Time to stabilization and peak GRF at landing | 50 Hz | | 3 | A standardized warm-up, consisting of slow running 2 minutes on a treadmill (10% incline, 10 km/h) and three drop jumps (drop height: 15 cm)  Left and right leg landing  Unanticipated jump landing: perform a counter-movement jump with a single-leg landing; at takeoff visual info depicts landing leg to be used.  Drop jump: performed from 32 cm box; start with bipedal hip-width stance on box: frontal step, drop, explosive reactive jump with shortest possible ground time; instructed to jump as high as they could | |  |
| Oberlander et al, 2013 [59] Cohort Germany  In English | PA: Soccer, basketball, skiing Level: N/R Time Since ACLR: 6 and 12 months ACLR: (m=N/R), (f=); Age: 28.0$\pm$7.0 y/o Control: N/A ACLD: N/A | To examine knee extensor muscular capacity and landing mechanics (i.e., trunk angle characteristics, joint kinetics, and postural dynamic stability control) in a group of unilateral ACL re- construction patients at 6 and 12 months after surgery, using an SLHT on the involved and uninvolved legs. Moreover, we aimed to combine the results from the current work with our previous findings (25) to identify potential changes in the extent of functional recovery, from the ACLD state to 6 and 12 months after ACLR using the same patients. | GRF (magnitude) | 1000 Hz | | 5 | Subjects performed a modified single leg hop test (SLHT) for distance, keeping their hands on their hips and wearing their own sports shoes. This hop was performed with 1 leg over a given distance of 0.75 x body height. Landing had to be on the force plate within a target area corresponding to the given distance +/- 5 cm. Subjects had to perform 5–10 valid SLHTs with each leg. | |  |
| *Orishimo et al, 2010 [84] Cohort USA  In English | PA: N/R Level: N/R Time Since ACLR: 7.2$\pm$2.7 months ACLR: (m=9), (f=4); Age: 33$\pm$10 y/o Control: N/A ACLD: N/A | To compare joint ranges of motion, joint moments and joint powers in the involved and noninvolved legs of patients who have had an ACL reconstruction as they performed the single-leg hop test. | Overall peak GRF magnitude, peak anterior GRF, peak vertical GRF | 960 Hz | | 3 | Alternating the involved and non-involved legs  Takeoff trials: subjects started in single-leg stance with their foot in the center of the force plate. They then performed a single-leg hop for maximal horizontal distance, landing on the same leg on the laboratory floor. There were no restrictions of upper extremity movement. Hop distance was measured from the toe in the starting position to the heel at landing. The average distance of the 3 takeoff trials was measured from the center of the force plate and marked as the starting point for the landing trials.   Landing trials: subjects started in single-leg stance with their toe at the starting point and performed 3 single-leg hops onto the force plate. | |  |
| Paterno et al, 2011 [60] Cross Sectional USA  In English | PA: Jumping, pivoting or cutting activity (Level I/II sports) Level: Competitive Time Since ACLR: 6.9$\pm$1.7 months ACLR: (m=21), (f=35); Age: 16.4$\pm$3.0 y/o Control: (m=12), (f=29); Age: 16.8$\pm$2.3 y/o ACLD: N/A | While asymmetries in loading patterns during dynamic landing tasks were observed in female athletes 2 years after ACLR, it is unknown if there are similar patterns of asymmetry at the time of RTS after ACLR in male athletes. Therefore, the purpose of this study was to determine if a similar pattern of lower limb asymmetries might exist at the time of RTS after ACLR in both males and females. | Peak VGRF (normalized to body weight), peak VGRF Limb Symmetry Index ((involved/un-involved)*100%) | 1200 Hz | | 3 | The participant was positioned on top of a 31-cm box and instructed to drop off the box, with both feet leaving the box simultaneously and each foot landing on a separate force platform, then to immediately execute a maximal effort vertical jump towards an overhead target | |  |
| Patterson et al, 2013 [61] Cross Sectional Ireland  In English | PA: Gaelic football, soccer, hockey, basketball Level: Club or county level Time Since ACLR: 42.0$\pm$38.9 months ACLR: (m=17), (f=0); Age: 20.8$\pm$1.1 y/o Control: (m=17), (f=0); Age: 22.6$\pm$3.4 y/o ACLD: N/A | To examine time to stabilization (TTS) values in a group of female athletes who had returned to full sports participation following ACL reconstruction as well as in a group of controls. TTS from a diagonal landing was compared to TTS from a forward landing. | Time to stabilization for forward land and diagonal land in: AP, ML, RV (resultant vector) directions | 2000 Hz | | 3 | Barefoot  Forward landing trials: each subject standing on top of a 35-cm box with feet shoulder width apart. They were then instructed to place their hands on their hips and to look straight ahead. After an audio cue, each subject stepped forward, leading with the test leg, and dropped from the step, landing on the force plate on the test leg only. Subjects were instructed to stabilize as quickly as possible upon landing and to hold a still position for 15 s   Diagonal landing task: subjects stood bare-foot in single-leg stance at the posterior lateral aspect of a force plate on the non-test leg. They were then required to perform a diagonal jump to land onto the middle of another force plate, land on the test leg and remain still for 15 s. Subjects were instructed to stabilize as quickly as possible upon landing. | |  |
| Pfeiffer et al, 2018 [62] Cross Sectional USA  In English | PA: N/R Level: N/R Time Since ACLR: 49.6$\pm$40.6 months ACLR: (m=9), (f=26); Age: 22.1$\pm$3.4 y/o Control: N/A ACLD: N/A | The primary purpose of this study was to determine if individuals with a unilateral ACLR, who demonstrate greater peak kinematic and kinetic magnitudes in the ACLR and uninjured limb during walking gait also demonstrate greater peak kinematic and kinetic magnitudes in each limb during jump-landing. Additionally, we will determine if those who demonstrate greater kinematic and kinetic asymmetries during walking gait also demonstrate greater asymmetries during jump-landing using limb symmetry indices (LSI). | Peak vGRF, instantaneous loading rate, linear loading rate | 1200 Hz | | 5 | Gait: Participants completed walking trials barefoot. During all walking gait trials, participants were instructed to walk at a self-selected speed over 2 force plates embedded in a staggered formation towards the middle of a 6m walkway so that the entire stance phase for both limbs could be collected during a single trial  Jump landing: All participants wore their own athletic footwear for the jump-landing trials. Participants performed jump- landing from a 30cm box positioned 50% of the participant’s height from the front edge of the force plates on each force plate, and immediately jump vertically as high as possible | |  |
| Pua et al, 2017 [63] Cohort Singapore  In English | PA: N/R Level: Competitive (65 participants) Time Since ACLR: 6 months ACLR: (m=60), (f=10); Age: 25.4$\pm$5.9 y/o Control: N/A ACLD: N/A | To evaluate the prospective associations among quadriceps strength and RTD and single-leg hop for distance, single-leg vertical jump, as well as the vGRF and loading rate during landing from a vertical jump in persons who have undergone ACLR. Also examined performance measures such as hop distance and jump height as they are common clinical measures and provide a gross measure of athletic performance | Normalized vGRF, loading rate | 1000 Hz | | 2 | Single leg vertical countermovement jump. Patients placed hands on hips, instructed to jump as high as they could while maintaining a stable landing | |  |
| **Read et al, 2020 [81] Cross Sectional Qatar  In English | PA: Soccer Level: Professional Time Since ACLR: 7.2$\pm$0.8 and months ACLR: (m=124), (f=0); Age: 23.8$\pm$6.1 y/o Control: (m=204), (f=0); Age: 24.2$\pm$4.7 y/o ACLD: N/A | Our primary aim was to compare the performances of each group with those of matched controls. The second aim was to determine which kinetic variables measured during the CMJ best distinguished between the healthy players and those with a history of ACLR. | Concentric impulse, concentric peak force asymmetry, eccentric mean force asymmetry, eccentric deceleration impulse asymmetry, eccentric rate of force development asymmetry, peak landing force asymmetry | 1000 Hz | | 3 | Countermovement jump. Patients stood upright with hands on hips, remained motionless on force plates for 3 sec, then performed downward motion to self-selected depth, and then immediate upward motion. | |  |
| Richter et al, 2019 [64] Cross Sectional Ireland  In English | PA: Multi-directional sport Level: N/R Time Since ACLR: 9.4$\pm$0.7 months ACLR: (m=156), (f=0); Age: 24.8$\pm$4.8 y/o Control: (m=62), (f=0); Age: 24.8$\pm$4.2 y/o ACLD: N/A | To develop and test a data driven framework (feature generation based on no expert or prior knowledge) to classify movement patterns of normal and rehabilitating athletes using only biomechanical data | vGRF | 1000 Hz | | 3 | A standardized warm-up  Shoes on  Subjects performed 7 exercises (only 3 had force plate data): double leg drop jump, single-leg hop, hurdle hop | |  |
| Rudroff et al, 2003 [65] Cross Sectional Germany  In English | PA: Soccer Level: N/R Time Since ACLR: 24 months ACLR: (m=30), (f=0); Age: 30.9$\pm$5.4 y/o Control: (m=10), (f=0); Age: 31.1$\pm$4.7 y/o ACLD: N/A | To compare the clinical outcome of ACL reconstruction using the four-strand ham- string tendon autografts and ACL reconstruction using the patellar tendon graft 2 yr. after surgery. | Vertical jump-off force, first vertical maximum | 1000 Hz | | Squats: 5 trials; gait, one- and two-legged jumps: 6 trials | Two-legged jump: jumped down from a 26-cm step 6 consecutive times  One-legged jump: initiated from standing anatomical position with hands on hips, flexed hip/knee to 90 degrees and performed maximal jump  Squats: in standing position, femurs rotated externally (feet abducted 20 degrees), lowered center of mass to 90 degrees at approx. 30 deg/sec  Gait: walk barefoot over 2 force plates 6 times; data of last 5 trials used | |  |
| Schilling et al, 2020 [66] Cohort USA  In English | PA: Sports Level: Collegiate Athlete Time Since ACLR: Range (6-71) months ACLR: (m=7), (f=14); Age: 20.3$\pm$1.7 y/o Control: N/A ACLD: N/A | To assess the readiness for return to sport in a sample of division III athletes following ACLR and medical clearance. | vGRF | N/R | | 3 | Participants performed a single maximum single-leg squat test (SLST) while standing on a 12 in.-high step (30.48 cm).   The participants were then asked to perform a single-leg landing task from the step and land on a force plate that was located 12 inches away from the step. | |  |
| Schneider et al, 2017 [67] Cross Sectional USA  In English | PA: Different sports Level: N/R Time Since ACLR: Returned to sport (i.e., more than 6 months) months Dominant/Non-dominant: N/R ACLR: (m=10), (f=26); Age: 16.4$\pm$1.1 y/o Control: (m=45), (f=22); Age: 17.3$\pm$1.9 y/o ACLD: N/A | To evaluate a mass-spring-damper model may serve as an extension of biomechanical data from 3 dimensional motion analysis and epidemiological data. | The force produced by the spring is  Fs = kspring*L The force produced by the damper is  Fd = kdamper*V given that L is the length of the spring (leg deformation) and V is the velocity of the mass.   Spring and Damper ratios LSI | 50 Hz | | 3 | Box height of 31 cm  Subjects were instructed to drop off the box, to land on the ipsilateral foot directly in front of the box and to hold the landing for 3 second  Subjects were instructed to line up at their individual starting positions located at 50% of their maximum double-limb broad jump distance (taken from a previous test). Subjects were instructed to initiate the movement while balancing on 1 foot, to hop as far forward as possible, and to land on the ipsilateral foot with their heels beyond a tapeline located at the front edge of a portable force plate. A landing stabilized for 1-second was required for a successful trial. | |  |
| Shimizu et al, 2019 [68] Cohort USA  In English | PA: N/R Level: N/R Time Since ACLR: Range (6-36) months ACLR: (m=17), (f=14); Age: 31.3$\pm$7.8 y/o Control: N/A ACLD: N/A | To investigate the longitudinal changes in landing mechanics and knee kinematics for patients both before and 3 years after ACLR and to investigate the association between changes in landing mechanics and magnetic resonance (MR) knee kinematics. | vGRF | 1000 Hz | | 3 | The drop-jump task, as previously described,11 involved the participant standing on a 30-cm high platform, stepping off with 1 foot, and landing with 1 foot on each of the force plates. The participant was instructed to land with both feet contacting the ground simultaneously and then immediately jump as high as possible. A successful trial was defined as 1 in which the participant stepped off the platform as opposed to jumping off or lowering himself or herself down, landed with both feet simultaneously with 1 foot on each force plate, and immediately performed a maximal vertical jump. Three successful drop-jump trials were collected and used for analysis. | |  |
| Shimizu et al, 2020 [69] Cohort USA, Japan  In English | PA: N/R Level: N/R Time Since ACLR: Range (6-36) months ACLR: (m=20), (f=16); Age: 31.3$\pm$7.8 y/o Control: (m=9), (f=5); Age: 31.4$\pm$4.9 y/o ACLD: N/A | (1) to investigate the longitudinal changes in meniscal T1r/T2 values and biomechanics during gait and landing tasks after ACLR   (2) to investigate the associations between changes in meniscal composition using T1r/T2 mapping and biomechanics in patients with ACLR. | Peak vGRF | 1000 Hz | | 3 | Shoes on  Participants were instructed to walk at a controlled speed of 1.35 m/s.  Standing on a 30-cm platform, stepping off with 1 foot, and landing with 1 foot on each of the force plates. The participant was instructed to land with both feet contacting the ground simultaneously and then immediately jump as high as possible. A successful trial was defined as 1 in which the participant stepped off the platform as opposed to jumping off or lowering himself or herself down, landed with both feet simultaneously with 1 foot on each force plate, and immediately performed a maximal vertical jump. Three successful drop-jump trials were collected and used for analysis. | |  |
| Shimizu et al, 2019 [70] Cohort USA  In English | PA: N/R Level: N/R Time Since ACLR: Range (6-36) months ACLR: (m=17), (f=14); Age: 31.3$\pm$1.4 y/o Control: (m=10), (f=6); Age: 31.7$\pm$1.3 y/o ACLD: N/A | To investigate the changes in landing biomechanics over a 3-year period and their correlation with cartilage degenerative changes in the MTFJ of the knee after ACLR using MR T1r mapping. | Peak vGRF vGRF impulse | 1000 Hz | | 3 | Standing on a 30-cm high platform, stepping off with 1 foot and landing with 1 foot on each of the force plates. The participants were instructed to land with both feet contacting the ground simultaneously and then immediately jump as high as possible | |  |
| Smeets et al, 2020 [71] Cross Sectional Belgium, UK  In English | PA: A sport that involves cutting, pivoting or jumping Level: Intermediate to high Time Since ACLR: 8.6$\pm$1.8 months ACLR: (m=15), (f=6); Age: 23.8$\pm$4.2 y/o Control: (m=15), (f=6); Age: 21.5$\pm$1.5 y/o ACLD: N/A | to combine conventional biomechanical observation (joint kinematics, kinetics, and muscle activations) to assess single-joint alterations (i.e., biomechanical changes in a single joint) with marker-based PCA to assess whole-body alterations (i.e., a combination of biomechanical changes in multiple joints) during landing strategies. Through this novel combined approach, we want to emphasize that RTS decision should consider both joint-specific alterations as well as whole-body compensatory movements. | vGRF | 1000 Hz | | 3 | A standardized warm-up included 5-min cycling on a stationary bike, 10 squats and 10 squat-jumps.  Single leg hop for distance: to jump as far as possible on 1 leg.  Medial and lateral hop: to jump sideways over a 0.24-mhigh hurdle (1.5 cm wide) on 1 leg, to cover a medio-lateral distance that was half the leg length (i.e., the distance between the anterior superior iliac spine and medial malleolus).  Vertical hop with 90° of medial rotation and vertical hop with 90° of lateral rotation: to jump as high as possible on 1 leg while performing an inward/outward rotation of 90°.  For all 3 tasks, participants were instructed to take off and land on the same leg. Trials were considered valid if the landing was central on the force plate and the participant could maintain his/her balance for 5 s after landing without shuffling on the stance leg. | |  |
| Tsai et al, 2012 [72] Cross Sectional USA  In English | PA: Athletes Level: Recreational Time Since ACLR: 36.2$\pm$18.5 months ACLR: (m=0), (f=10); Age: 25.3$\pm$2.4 y/o Control: (m=0), (f=10); Age: 24.9$\pm$1.7 y/o ACLD: N/A | To examine tibio-femoral compressive and shear forces as well as muscle co-contraction and knee flexion during a single-leg drop-land task between females who have undergone ACLR and healthy female controls. | GRF | 1500 Hz | | 3 | 25cm high platform  For the single-leg drop-land task, subjects started from a single-leg standing position on a platform in front of the force plate. Subjects were instructed to land with the tested foot on the force plate and then jump upward as high as possible. | |  |
| Vairo et al, 2008 [73] Experimental USA  In English | PA: Athletes Level: Recreational  Time Since ACLR: 21.4$\pm$10.7 months ACLR: (m=5), (f=9); Age: 22.5$\pm$4.1 y/o Control: (m=5), (f=9); Age: 22.8$\pm$3.5 y/o ACLD: N/A | To investigate the effects of ISGA ACLR on neuromuscular and biomechanical performance during a single-leg vertical drop landing (VDL). | vGRF Peak vGRF | 1200 | | 3 | Standing erect upon only the lower extremity being tested with the foot in neutral position, participants stepped off a 30 cm high platform placed 11 cm from the edge of the force-plate. Participants were instructed to land in the center of the force-plate on the lower extremity being tested only. To control for countermovement, participants were restricted to perform VDLs with hands upon hips and the contra-lateral knee joint flexed to 90. It was also stressed that the non-tested shank segment did not come into contact with the tested lower extremity. This aimed at limiting horizontal displacement and enabled the participant to land with a more vertical approach. Following a verbal cue, participants dropped off the platform and landed upon the force-plate | |  |
| Ward et al, 2018 [74] Cross Sectional Australia  In English | PA: Athletes Level: Moderate Time Since ACLR: 52.0$\pm$42.0 months ACLR: (m=7), (f=21); Age: 22.4$\pm$3.7 y/o Control: N/A ACLD: N/A | To evaluate the associations between indices of quadriceps neuromuscular function (strength, voluntary activation, and spinal-reflex and cortico-motor excitability) and sagittal-plane knee kinetics (peak KEM), kinematics (knee-flexion angle at initial contact [IC], peak knee-flexion angle, and knee flexion excursion), and peak vGRF during jump landings after ACLR. | Peak vGRF | 1200 | | 3 | Shoes on  Participants performed a jump-landing task from a 30-cm box positioned at 50% of the participant’s height from the front edge of the force plates.31 We instructed them to jump forward off the box to a double-legged landing with 1 foot on each force plate and then immediately jump vertically as high as possible. A trial was considered successful if the participant left the box with both feet at the same time, landed on the force plates, and jumped straight up in the air. If the trial was unsuccessful, a subsequent trial was collected for analysis. | |  |
| Webster et al, 2004 [75] Cross Sectional Australia  In English | PA: Sport Level: High Time Since ACLR: 11.6$\pm$2.6 months ACLR: (m=18), (f=2); Age: 25.5$\pm$6.4 y/o Control: N/A ACLD: N/A | To examine and compare sagittal plane joint angles, moments and ground reaction forces in patients with hamstring tendon (HS) and PT graft ACL reconstructions during 2 functional landing tasks. | Peak vGRF | 400 Hz | | 6 | 15 cm high box  Subjects were instructed to stand on the test leg, place their hands on their hips and, at the experimenter’s count of three, hop forward and land on the same leg, in the center of the force plate. They were further instructed that on landing they were to look straight ahead and stabilize as quickly as possible. Once the experimenter had judged that the subject was stable a cue was given to notify the subject of completion of the activity | |  |
| Webster et al, 2004 [76] Cross Sectional Australia  In English | PA: N/R Level: N/R Time Since ACLR: 7.5 (range 6–9) months ACLR: (m=5), (f=3); Age: 25 (range 18-32) y/o Control: N/A ACLD: N/A | To determine whether, compared to the barefoot state, wearing sports shoes influenced knee kinetics and kinematics during single limb landing in subjects following ACL reconstruction. | Peak vGRF | 400 Hz | | 6 | Subjects were required to perform one-legged vertical hops from a 15 cm high box on to the force plate, both barefoot and whilst wearing sports shoes. The subject was directed to stand on the test leg, with hands on hips, and at the count of three, hop forward and land on the center of the force plate. They were required to keep the foot fixed at the landing position until stable at which time they were instructed by the experimenter to ‘‘walk forward’’ to clear the force plate. | |  |
| Webster et al, 2010 [77] Cross Sectional Australia  In English | PA: Collegiate athletics Level: Division 1 Time Since ACLR: 30$\pm$14.2 months ACLR: (m=0), (f=12); Age: 20.5$\pm$1.2 y/o Control: (m=0), (f=12); Age: 19.3$\pm$1.1 y/o ACLD: N/A | to use TTS to measure differences in dynamic postural control during jump landings in ACLR knees compared with healthy knees among Division I female athletes. | Time to stabilization (TTS) | 180 Hz | | 3 | Athletic clothing and athletic shoes  Participants were instructed to stand behind a mark on the floor that was 70 cm away from the center of the force platform. They were instructed to jump off anteriorly from 2 feet, hit the target on the Vertec with their fingers, and land on the force platform on the designated foot. All participants were right-hand dominant and used the right hand to hit the Vertec. They were instructed to ‘‘stick the landing,’’ place their hands on their hips as soon as possible, and hold the position as motionless as possible for 10 seconds.36 These were the only restrictions placed on the technique of the jump and landing. | |  |
| Wren et al, 2018 [78] Retrospective Cohort USA  In English | PA: N/R Level: N/R Time Since ACLR: 7.2$\pm$1.3 months ACLR: (m=19), (f=27); Age: 15.6$\pm$1.7 y/o Control: (m=12), (f=24); Age: 14.7$\pm$1.5 y/o ACLD: N/A | To assess biomechanics and symmetry of adolescent athletes following ACLR during a single leg hop for distance. | Peak GRF | 2400 Hz | | 3 | 5-min warm-up  For the single leg hop, participants were instructed to stand on 1 leg and jump as far as possible, landing on the same leg on a target force plate. For a trial to be successful, participants were required to stick the landing for a minimum of 2 seconds. | |  |
| Studies used Contact Mats Connected to a Jump system to assess "Jumping" (n=3) | | | | | | | | |  |
| Borin et al, 2017 [87] Experimental  Brasil  In English | PA: Athletes Level: N/R Time Since ACLR: 22.9$\pm$16.1 months ACLR: (m=18), (f=8); Age: Range (18-30) y/o Control: N/A ACLD: N/A | To evaluate the effects of a specific training program for the hip musculature on the functional alterations of athletes of both genders submitted to ACL reconstruction surgery. | Jump height Total Power Relative Power | N/R | | 3 | In order to evaluate the explosive strength of the lower limbs, the technique of Counter Movement Jump (CMJ) was used with the aid of the arms. Athletes stood with the trunk erect and knees in 180° extension. Then, the knees were flexed to ~120° followed by knee 81 extension push the body vertically. The knees remained in extension during the flight phase. The interval between attempts was 10 sec. Three attempts were made from which the best jump was recorded. | |  |
| Papandrea et al, 1990 [90] Cross Sectional Italy  In Italian | PA: Volleyball Level: N/R Time Since ACLR: 24.0 months (SD:N/R) ACLR: (m=2), (f=10); Age: 23.0 (SD:N/R) y/o Control: N/A ACLD: N/A | To analyze jumping ability of volleyball players after ACLR. | LSI | N/R | | 3 squat jumps 10 CMJs | N/R | |  |
| Petschnig et al, 1997 [91] Cross Sectional Austria  In English | PA: N/R Level: N/R Time Since ACLR: 13.7$\pm$1.1 months ACLR: (m=27), (f=0); Age: 28.1$\pm$0.9 y/o Control: (m=50), (f=0); Age: 29.3$\pm$1.1 y/o ACLD: N/A | The first was to further evaluate dynamometric measurements, single and trip hop tests, and one-legged and two-legged vertical jump tests. These tests were chosen to compare untrained subjects with no prior history of knee injury and patients after ACL reconstruction with respect to the uninvolved leg. In addition, we attempted to determine differences between the patient's involved and uninvolved legs, and the dominant and non-dominant leg in a control group. The second objective was to determine the relationship between knee extensor strength of the quadriceps, one-legged hop test for distance, and the vertical jump test. We were also looking for a relationship between the one-legged and two-legged vertical jump tests. The final goal was to determine if there existed any differences in 2 different phases of the follow-up period. | Jump heights calculated from flight time | N/R | | 3 | One-legged and two-legged jump vertical jump: subjects performed three 10-s jumping trials at maximum frequency and jumping as high as possible. Subjects were instructed to keep both their hands on the hips to avoid using them for generating momentum. Best trial used for analysis. | |  |
| Studies used Single-sensor Insoles to assess "landing" (n=2) | | | | | | | | |  |
| Peebles et al, 2019 [92] Cohort USA  In English | PA: N/R Level: N/R Time Since ACLR: 6.9$\pm$1.2  ACLR: (m=9), (f=21); Age: 19.4$\pm$4.2 y/o Control: N/A ACLD: N/A | The purpose of the present study was to determine the effect of wearing a custom fit extension constraint functional knee brace on hop distance and plantar loading symmetry during a single, triple, and crossover hop test throughout the RTS transition in patients with ACLR | Limb symmetry index (%) for each hop type for: impact peak, loading rate, impulse | 100 Hz | | 2 | With vs. without brace; single hop vs. triple hop vs. crossover hop Each of these hop tests were completed both while wearing a custom fit functional knee brace on the surgical limb and without at each testing visit. Single hop: participants were instructed to hop as far as possible while taking off and landing on the same foot.  Triple hop: participants hopped 3 consecutive times without pausing in between hops and the cumulative distance was recorded.  Crossover hop: participants again hopped 3 consecutive times without pausing, but had to laterally cross over a 6- inch-wide strip with each hop while progressing forwards. | |  |
| Peebles et al, 2019 [93] Cross Sectional USA  In English | PA: N/R Level: Recreational Time Since ACLR: 6.95$\pm$1.27 months ACLR: (m=6), (f=19); Age: 18.7$\pm$3.0 y/o Control: (m=12), (f=18); Age: 22.2$\pm$3.8 y/o ACLD: N/A | The first purpose of this study was to compare hop distance symmetry and loading symmetry between ACLR athletes at the time of return to sport and healthy uninjured recreational athletes. The second purpose of this study was to determine the association between hop distance symmetry and loading symmetry | Limb symmetry index (%) for each hop type for: impact peak, loading rate, impulse | 100 Hz | | 2 | During the single hop test, participants were instructed to hop as far as possible while taking off and landing on the same foot. For the triple hop test, participants hopped 3 consecutive times without pausing be- tween hops. Similarly, for the crossover hop test, participants hopped 3 consecutive times without pausing; however, they had to laterally crossover a 6-inch wide strip while still hopping forward. Participants crossed the strip toward the non-hopping leg on the first and third hop and toward the hopping leg on the second hop (38,39). For all 3 tests, participants were required to stick the final landing, defined as maintaining balance for 2 s without touching the ground with the contralateral leg or either hand. | |  |
| Studies used Balance Platforms to assess "jumping" (n=1) | | | | | | | | |  |
| Czamara et al, 2011 [88] Cohort Poland  In English/Polish | PA: Team games, athletics, skiing and dancing Level: 17 athletes competitive 46 athletes amateur Time Since ACLR: 6.0 months (SD:N/R) ACLR: (m=38), (f=25); Age: 27.0$\pm$8.0 y/o Control: N/A ACLD: N/A | An objective evaluation of physical fitness level in the athletes after ACLR, allowing them to return to training, using the measurement devices in the form of a tailored sport-related determinant of functional assessment | Number of jumps Peak and minimum values of GRF | N/R | | N/R | One leg jumps with the measurement of ground reaction force values (N) for the vertical component on the MTD balance platform and counting the number of jumps performed. | |  |
| Studies used Pressure Mats to assess "jumping" (n=1) | | | | | | | | |  |
| Dan et al, 2019 [89] Cross Sectional Australia  In English | PA: N/R Level: N/R Time Since ACLR: Range (8-15) months ACLR: (m=47), (f=18); Age: 33.8$\pm$10.1 y/o Control: (m=17), (f=20); Age: 25.1$\pm$8.5 y/o ACLD: N/A | to explore the utility of this accelerometer and gyroscope system as well as a pressure sensing mat (MatScan, TekScan, South Boston, Massachusetts, USA) in detecting kinetic differences in patients prior to return to sport following ACL reconstruction. | Peak Load flight time | N/R | | N/R | Barefoot Single and double leg | |  |
| *N/R* Not reported, *N/A* Not applicable, *PA* Physical activity, *Level* Activity level, *SD* standard deviation, *IQR* Interquartile range, *ACLR* Anterior cruciate ligament reconstruction, *ACLD* anterior cruciate ligament deficient, *GRF* Ground reaction force, *vGRF* vertical ground reaction force, *pGRF* Posterior grand reaction force, *CoP* center of pressure, *LSI* limb symmetry index, *N/Kg* Newton per kilogram, *AP* anterior posterior, *ML* medial lateral, *RV* resultant vector.  * Studies used force plate for "Landing and Jumping" (n=3)  **Studies used force plate for "Jumping" (n=3) | | | | | | | | |  |

Table 2 Data Extraction Table for Studies Assessing Standing Balance

| **Study Characteristic**  **(author, year, design, country, language)** | **Sample Characteristic**  **(Physical Activity (PA), level, time since surgery, side of surgery, sample size by sex, age)** | **Study Objectives** | **Parameters** | | **Sampling Frequency** | **Number of Repetitions** | | - **Testing Condition or Challenges** - **Protocol Summery** | |
| --- | --- | --- | --- | --- | --- | --- | --- | --- | --- |
| **Studies used Force Plates to assess "Standing Balance" (n=23)** | | | | | | | | | |
| Ahmadi et al, 2020 [15] Quasi Experimental Iran  In English | PA: Soccer players Level: N/R Time Since ACLR: 14.10$\pm$3.99 months ACLR: (m=20), (f=0); Age: 26.55$\pm$3.54 y/o Control: (m=20), (f=0); Age: 25.95$\pm$4.88 y/o ACLD: N/A | Compare external focus (EF) and continuous cognitive task (CCT) on postural stability after ACL reconstruction | Postural sway area, displacement (AP) and (ML) in CoP, velocity of CoP, and the mean power frequency of the CoP | 100 Hz | | 3 | | External focus, continuous cognitive task, control condition  Maintaining balance while standing on a wobble board on a force plate and then calculating balance related outcomes based on measured CoP trajectories | |
| Birmingham et al, 2001 [94] Cross Sectional Canada  In English | PA: N/R Level: N/R Time Since ACLR: 19.4$\pm$14.5 months ACLR: (m=15), (f=15); Age: 27.2$\pm$11.3 y/o Control: N/A ACLD: N/A | To evaluate the effects an ACL brace has on measures of knee proprioception and postural control assessed using testing situations that involved differing sensory inputs and that challenged postural control to varying degrees | CoP length of path CoP medio-lateral displacement CoP anterior-posterior displacement | 60 Hz | | 3 | | Brace/no-brace conditions Single leg standing on a stable platform/7 cm medium density poly-foam Eyes open/closed  The balance tests included: 1) standing on the stable platform with eyes open, 2) standing on a foam mat placed over the platform with eyes open, 3) standing on the platform with eyes closed, and 4) standing on the platform after landing from a maximal single-limb forward hop | |
| Bodkin et al, 2018 [95] Cross Sectional USA In English | PA: N/R Level: N/R Time Since ACLR: 6.5$\pm$1.4 months ACLR: (m=59), (f=49); Age: 21.7$\pm$8.3 y/o Control: (m=51), (f=49); Age: 21.9$\pm$4.0 y/o ACLD: N/A | To assess single-leg postural control in healthy individuals and ACLR patients around the time point of return to sport progression. | CoP velocity | 50 Hz | | 3 | | Starting with the non-involved side  Alternating single leg balance for 10 seconds on each leg, and repeated 3 times. | |
| Bonfim et al, 2005 [96] Cross Sectional Brazil  In Portuguese | PA: N/R Level: N/R Time Since ACLR: 19.6$\pm$6.5 months ACLR: (m=N/R), (f=); Age: 24.4$\pm$4.5 y/o Control: (m=N/R), (f=N/R); Age: 24.4$\pm$3.0 y/o ACLD: N/A | To examine if the performance of postural control of individuals post ACL reconstruction is dependent on the task performed. Specifically, to examine the breadth, the speed and the average frequency oscillation and the displacement area of ​​the center of mass and the pressure center, in the bipedal position and mono-podal, in individuals ACL reconstruction. | Center of pressure displacement in anterior-posterior, and medio-lateral.  Center of pressure velocity | 100 Hz | | 3 | | Standing on 2 legs, arms crossed on chest, eyes closed Standing on right leg, arms crossed on chest, eyes closed, hip neural, knee in 90 flexion Standing on left leg, arms crossed on chest, eyes closed, hip neural, knee in 90 flexion  Three trials for each testing condition. Each trial lasted for 30 seconds, with one-minute break in between trials. | |
| Bonfim et al, 2003 [97] Cross Sectional Brazil  In English | PA: N/R Level: N/R Time Since ACLR: 18 (range 12-30) months ACLR: (m=7), (f=3); Age: 24.4$\pm$4.5 y/o Control: (m=7), (f=3); Age: 24.4$\pm$3.0 y/o ACLD: N/A | To examine position perception and threshold for detection of passive knee motion, latency onset of hamstring muscles, and upright stance control in individuals who had undergone ACL reconstruction | Center of pressure displacement in anterior-posterior, and medio-lateral. | 100 Hz | | 3 | | Standing barefoot on 2 legs, arms crossed on chest, eyes closed Standing on right leg, arms crossed on chest, eyes closed, hip and knee in 90 flexion Standing on left leg, arms crossed on chest, eyes closed, hip neural, knee in 90 flexion  Assuming the testing positions (conditions) during 3 trials for each testing condition. Each trial lasted for 30 seconds, with 1 minute break in between trials. | |
| Brunetti et al, 2006 [98] Experimental  Italy   In English | PA: N/R Level: N/R Time Since ACLR: 9.0 months (SD:N/R) ACLR: (m=30), (f=0); Age: 25.0$\pm$3.0 y/o Control: N/A ACLD: N/A | To find a new method of applying vibratory stimulation in order to permanently restore balance and motor function in patients having undergone ACL reconstruction | CoP velocity Elliptic area (CoP displacement) | 100 Hz | | N/R | | Standing on 1 leg with the knee flexed at 15, the hip joint fully extended, and with their arms crossed over the chest. Eyes open/closed  Standing on the force plate on 1 leg with the knee flexed at 15, the hip joint fully extended, and with their arms crossed over the chest, eyes open/closed for 20 seconds. Short break was given between trials. | |
| DiFabio et al, 2018 [99] Cross Sectional USA  In English | PA: Exercise regularly Level: Recreational Time Since ACLR: 6.86$\pm$3.07 months ACLR: (m=35), (f=41); Age: 21.8$\pm$8.4 y/o Control: (m=35), (f=19); Age: 23.4$\pm$13.1 y/o ACLD: N/A | To determine if the different components of the a Lower Extremity Assessment Protocol (LEAP) provide unique information regarding performance and symmetry in both ACLR and healthy participants using an exploratory factor analysis | Average center of pressure velocity and area of displacement of the center of pressure | 50 Hz | | 3 | | Participants warmed up with 5-min of treadmill walking at a self-selected speed.  Single leg, hands on hips and eyes closed  Participants were tested on the uninvolved or dominant limb first, in a single leg stance with the foot in the middle of the force plate. Participants were instructed to stand on a single limb to hold the position for 10 seconds with their eyes closed and hands on hips. The test was then repeated on the involved limb. If the participant fell out of position, opened their eyes, or put the opposite foot down, the test was repeated. | |
| Dingenen et al, 2015 [100] Cross Sectional Belgium  In English | PA: N/R Level: N/R Time Since ACLR: 23.0$\pm$14.0 months ACLR: (m=5), (f=15); Age: 22.3$\pm$2.3 y/o Control: (m=5), (f=15); Age: 23.4$\pm$2.6 y/o ACLD: N/A | To evaluate postural stability during the transition from DLS to SLS in ACLR subjects and non-injured control subjects. | Contralateral push-off CoP excursion Contralateral push-off CoP displacement Peak CoP velocity Time to new stability point Mean absolute CoP velocity during intermediate phase CoP displacement during 3 seconds after time to new stability point | 500 Hz | | 3 | | Barefoot. Arms hanging at the side. Double/single leg Eyes open/closed  Participants were asked to stand barefoot on a force plate with the feet separated by the width of the hips and the arms hanging loosely at the side. They performed a transition task from DLS (13 s) to SLS (13 s). Both legs of both groups were tested. The leg that was tested first was assigned randomly. The position of the feet during DLS was indicated on a paper lying on the force plate to ensure that subjects returned to the same starting position after each trial. Subjects were instructed to lift 1 leg on the command of the examiner toward approximately 60 of hip flexion within 1 s, using a metronome as a reference. As most postural stability outcomes during this experimental task can be influenced by the speed, it was suggested to standardize the speed of the transitional movement among the 2 groups | |
| Dingenen et al, 2016 [101] Cross Sectional Belgium  In English | PA: N/R Level: N/R Time Since ACLR: 23.0$\pm$14.0 months ACLR: (m=5), (f=15); Age: 22.3$\pm$2.3 y/o Control: (m=5), (f=15); Age: 23.4$\pm$2.6 y/o ACLD: N/A | To investigate muscle activation onset times of knee, hip and ankle muscles of both legs in ACLR and non-injured control subjects | Peak CoP velocity | 500 Hz | | 3 | | Barefoot. Arms hanging at the side. Double/single leg. Eyes open/closed  Participants were asked to stand barefoot on a force plate with the feet separated by the width of the hips and the arms hanging loosely at the side. They performed a transition task from DLS (13 s) to SLS (13 s) (Fig. 1). Both legs of both groups were tested. The leg that was tested first was assigned randomly. The position of the feet during DLS was indicated on a paper lying on the force plate to ensure that subjects returned to the same starting position after each trial. Subjects were instructed to lift 1 leg on the command of the examiner toward approximately 60 of hip flexion within 1 s, using a metronome as a reference. As most postural stability outcomes during this experimental task can be influenced by the speed, it was suggested to standardize the speed of the transitional movement when comparing non-injured and pathological subjects | |
| Ferdowsi et al, 2018 [14] Cross Sectional Iran  In English | PA: Soccer Level: Recreational Time Since ACLR: 16.0 months (SD:N/R) ACLR: (m=20), (f=0); Age: 27.2$\pm$3.7 y/o Control: (m=20), (f=0); Age: 26.2$\pm$3.2 y/o ACLD: N/A | To assess the intra- and inter-session test retest reliability of balance due to the transitional task from DLS to SLS in athletes with and without ACLR | CoP displacement for range sideways (Rsw) and range fore-aft (Rfa), area, and the mean velocity (Mv) of CoP | 500 Hz | | 3 | | Barefoot  Eyes open at a fixed point localized on a facing wall. Each testing procedure started with a 25 second DLS where the athletes were asked to stand barefoot on the center of a single force platform and kept the arms along the body. Next, the athletes were instructed to do transition to SLS on their legs while they maintained 60° hip flexion for 30 seconds on their tested leg. Finally, the athletes’ transition to DLS for 5 seconds on a line lying on the center of the force plate ensure that they were localized at the correct position. It is necessary to mention that the first 5 seconds of SLS phase was considered as the loading phase, while the last 5 seconds of the total testing procedure was regarded as the unloading phase | |
| Frank et al, 2014 [35] Quasi Experimental USA  In English | PA: N/R Level: N/R Time Since ACLR: 35.0$\pm$16.9 months ACLR: (m=0), (f=14); Age: 19.6$\pm$1.5 y/o Control: N/A ACLD: N/A | To investigate the effects of fatigue on lower extremity biomechanics and postural control in active females with ACLR | vGRF CoP sway speed | 1400 Hz | | 5 Double leg jump landings 3 single leg balance | | Participants performed 5-min of light; self-directed stationary bike warm-up followed by 5-min of self-directed stretching prior to testing.  Double-Leg Jump Landing Jump of a 30-cm box placed at a distance equal to 1 half the participant’s height from the leading edge of the force plate. On landing, participants were instructed to jump up as high as possible.   Single-Leg Balance  Barefoot. Hands on hips Eyes closed while standing unshod atop the center of a force plate. Participants were instructed to place their hands on their hips for the duration of the balance task. Each participant attempted to balance on their ACLR limb for 20 seconds while center-of-pressure (COP) data were recorded. | |
| Furlanetto et al, 2016 [36] Cross sectional Brazil  In English | PA: N/R Level: N/R Time Since ACLR: 6.0 months (SD:N/R) ACLR: (m=N/R), (f=); Age: 29.2$\pm$8.1 y/o Control: (m=N/R), (f=N/R); Age: 27.8$\pm$4.0 y/o ACLD: N/A | Evaluate and compare proprioception, postural control and knee function in subjects with and without unilateral ACL reconstruction | Postural control (PC): Amplitude of CoP (anterior-posterior and medial-lateral directions) Step up and down (SUD): first peak vGRF, load application rate (LAR) | 2000 Hz | | 3 trials each (PC), 5 trials each (SUD) | | Postural control (PC): right and left uni-podal support lasting for 30 sec each. The individual was asked to remain still in the indicated position with his/her hands on the anterosuperior iliac crests (ASIC), silently, gaze fixed on a target, located 1m from the eye level of each participant.   Step up and down (SUD): Start with L and R lower limb. 30 cm high wooden box placed on 1st force plate. The test started off the force platform with the patient in static position, legs together and hands on ASIC. The individual climbed the step with 1 of his/her LLs and descended it by stepping on 2nd force plate in a continuous single movement. | |
| Goetschius et al, 2013 [102] Cross Sectional USA  In English | PA: N/R Level: Recreationally active Time Since ACLR: 60$\pm$51.6 months ACLR: (m=10), (f=10); Age: 25.5$\pm$5.5 y/o Control: (m=10), (f=10); Age: 24.6$\pm$5.0 y/o ACLD: N/A | This study aimed to compare the effects of 36 min of continuous exercise on postural control and joint reposition acuity in patients with anterior cruciate ligament reconstruction (ACL-R) and healthy controls. | CoP excursions (medial-lateral and anterior-posterior directions), CoP velocity, CoP area |  | | 3 | | Uni-pedal, eyes closed  Participants standing on the test limb, foot centered on the force plate, contralateral hip and knee flexed to 30 degrees and 45 degrees, and arms held across the chest | |
| Head et al, 2019 [19] Cross Sectional USA, Canada  In English | PA: N/R Level: N/R Time Since ACLR: 7.6 (range 5.3-10.3) months ACLR: (m=4), (f=11); Age: 18.1$\pm$2.9 y/o Control: (m=4), (f=11); Age: 18.1$\pm$2.9 y/o ACLD: N/A | To examine dynamic postural stability using the Dynamic Postural Stability Index (DPSI) in athletes following ACLR at the time of release for RTS, and to compare these findings with healthy controls. A secondary purpose was to examine differences in dynamic postural stability between the involved and uninvolved lower extremities in the ACLR group. | Dynamic Postural Stability Index (DPSI) | 1200 Hz | | 3 | | A f-min warm-up on a stationary bike.  Single limb jump-landing tasks: forward jump (FJ), lateral jump (LJ), and diagonal jump (DJ)  Subjects completed a series of single-limb jump-landing tasks. Subjects were instructed to land on the pre-determined test leg in the center of the force plate, stabilize as quickly as possible, and balance for 10 s with hands on hips, facing straight ahead | |
| Heinert et al, 2018 [103] Cross Sectional USA  In English | PA: N/R Level: N/R Time Since ACLR: 13.9$\pm$4.7 months ACLR: (m=7), (f=7); Age: 18.5$\pm$3.8 y/o Control: N/A ACLD: N/A | The purpose of this study was to examine the DPSI in a surgically reconstructed ACL limb compared to the uninjured leg in athletes that had been cleared for sport. | Stability indices (medial-lateral, anterior-posterior, vertical and total (DPSI)) | 1200 Hz | | 5 | | A 5-min warm-up on a stationary bike at self-selected speed.  Standardized footwear, single leg hop  Subjects performed single landings over a 12-inch hurdle in the anterior direction onto a force platform. The participants began from a distance corresponding with 40% of their height to the force platform. They were allowed to use their arms to propel themselves over the barrier and assist with obtaining postural control. Participants were given instructions to place their hands on their hips immediately following stabilization and hold that position for 10 seconds. | |
| Hoch et al, 2018 [104] Cross Sectional USA  In English | PA: N/R Level: N/R Time Since ACLR: 50.4$\pm$34.8 months ACLR: (m=1), (f=19); Age: 23.2$\pm$4.3 y/o Control: (m=4), (f=36); Age: 22.9$\pm$3.1 y/o ACLD: N/A | 1- To examine the differences in PROMs and CBOs between post-ACLR and healthy control participants  2- To determine the diagnostic accuracy and cut-off scores of these outcomes in order to discriminate between post-ACLR and healthy control participants | (TTB-mean minima) was measured in seconds and provided an estimate of the average amount of time it took the subject to make postural corrections and was assessed in both the anterior-posterior (TTB-mean-minima-AP) and medio-lateral (TTB-mean-minimal-ML) directions | N/R | | 3 | | Single limb (ACLR). Hands on hips. Eyes closed/open  Subjects were instructed to balance on the test limb, keep their hands on their hips at all times, and remain as still as possible. Each subject performed 1 practice and 3 test trials for 10-seconds each on the test limb, first with their eyes open (EO), then eyes closed (EC). | |
| Hoffman et al, 1999 [105] Cross Sectional USA  In English | PA: N/R Level: N/R Time Since ACLR: 9.5 months (SD:N/R) ACLR: (m=8), (f=12); Age: 23.4$\pm$5.8 y/o Control: (m=13), (f=7); Age: 24.0$\pm$4.1 y/o ACLD: N/A | To investigate quadriceps strength and static and dynamic balance in the ACL reconstructed patient and to compare these findings with an age-matched, injury-free control group. | Sway path linear mean Dynamic phase duration (perturbation phase) | 50 Hz | | 4 | | A warm-up session (no details)  Single-leg stance on a force plate, place hands on hips, and focus on a visual target placed 1m away at the eyes level Each participant of the experimental group performed 4 20-second trials on both the legs.  Dynamic balance was done in the same way in static, but with electric perturbation through stimulating the tibial nerve of the supporting leg. | |
| Kuster et al, 1999 [47] Quasi Experimental Swizerland  In English | PA: N/R Level: N/R Time Since ACLR: 33.6$\pm$7.2 months ACLR: (m=24), (f=12); Age: 31.7$\pm$9.9 y/o Control: N/A ACLD: N/A | “The present study used the one-legged stance test to evaluate the enhancement of muscle control and coordination afforded by the use of an elastic compression sleeve after ACL reconstruction. It also included a one-legged drop jump to further stress the balance control system.” | Peak impact loading for the landing phase, force-time integrals, path length (PL), root mean square error,  CoP | 100 Hz | | 3 | | With/without compression sleeves  Participants were required to perform a standing drop jump from a 10-cm-high platform onto a force plate landing on 1 leg and thereafter maintain a one-legged balance for 25 s. This task was repeated on the previously injured leg 3 times without and 3 times with an elastic compression sleeve. | |
| Pahnabi et al, 2014 [106] Cross Sectional Iran  In English | PA: Football Level: Competitive Time Since ACLR: 7$\pm$0.5 months ACLR: (m=N/R), (f=N/R); Age: 23.1$\pm$1.0 y/o Control(m=N/R), (f=N/R); Age: 23.0$\pm$1.1 y/o ACLD: N/A | To determine the sway of the center for gravity in football players with and without ACL reconstruction 7 months after the surgery | Medio-lateral axis (distance X) & anterior–posterior axis (distance Y) movement distance of CoP; velocity of CoP sway | 100 Hz | | 3 | | Left and right side; open and closed eyes; barefoot  All tests were done in unilateral standing on the bare foot on each side. Three trials were carried out with open and closed eyes. When the eyes were open, the subjects’ glance aimed at a fixed point at a 1-m distance on the front wall. The test duration was 30 seconds while keeping the arms along the body. On unilateral standing, the stance foot was at the center of the zero reference of the platform and the testing leg had contact with the opposite leg. Static unilateral standing tests began with the non-ACLR side or ACLR side randomly. The knee was positioned in the 20 degree angle of flexion, valgus and internal rotation (posture of injury). | |
| Peultier-Celli et al, 2017 [107] Cohort France  In English | PA: N/R Level: Amateur or professional Time Since ACLR: 6.0 months (SD:N/R) ACLR: (m=47), (f=20); Age: 29.1$\pm$7.5 y/o Control: N/A ACLD: N/A | To compare an innovative rehabilitation protocol with an “aquatic part” (balneotherapy) and a “dry part” and a conventional rehabilitation protocol (1) in terms of dynamics of recovery and development of the proprioceptive skills in athletes with ACL reconstruction. A secondary objective was to compare both groups in terms of functional improvement, i.e., pain, joint amplitude, muscular strength, and walking performance. | Sway area, sway path, somatosensory contribution to postural control (R_SOM_), the visual contribution to postural control (R_VIS_), the vestibular contribution to postural control (R_VEST_) | 40 Hz | | N/R | | Eyes open on firm support, eyes closed on firm support, vision altered on firm support, eyes open on foam support, eyes closed on foam support, vision altered on foam support  Each subject was asked to stand upright on the platform, barefoot, feet abducted at 30°, heels separated by 3 cm, arms along the body, remaining as stable as possible and breathing normally in 6 conditions to test somatosensory cues | |
| Stensdotter et al, 2013 [108] Cross Sectional Sweden  In English | PA: N/R Level: Moderate  Time Since ACLR: 19.6$\pm$0.9 months ACLR: (m=18), (f=10); Age: 44.7$\pm$4.4 y/o Control: (m=18), (f=10); Age: 47.0$\pm$5.0 y/o ACLD: (m=12), (f=6); Age: 46.2$\pm$4.8 y/o | To compare the ability for single-limb stance more than 20 years after unilateral ACL injury across 2 groups who had either rehabilitation including ACL reconstruction or a tailored physiotherapy program, and compared with knee-healthy controls. | CoP Path CoP SD in ML direction CoP SD in AP direction | 1200 | | N/R | | Eyes open. Barefoot. Arms folded across chest. Controlled room temperature. Silence was maintained  The uplifted foot was held apart from the stance leg, thighs and knees not touching. No further restrictions for leg positions were issued. The person was asked to stand as still as possible, i.e. not to talk or move head and arms, and abstain from all movements not involved in keeping the balance. ACL-injured subjects stood on the uninjured leg first and thereafter on the injured leg. Knee-healthy subjects always started on their dominant leg. | |
| Stensdotter et al, 2016 [109] Cross Sectional Norway  In English | PA: N/R Level: Moderate Time Since ACLR: 20.2$\pm$2.4 months ACLR: (m=21), (f=10); Age: 46.0$\pm$4.1 y/o Control: (m=13), (f=7); Age: 46.7$\pm$4.9 y/o ACLD: (m=22), (f=9); Age: 48.2$\pm$5.5 y/o | To compare postural sway and control strategies in 2 groups of ACL-injured subjects (with or without reconstructive surgery) to uninjured control subjects. | CoP Path CoP SD in ML direction CoP SD in AP direction | 1200 Hz | | N/R | | Barefoot  Quiet standing was performed on a force plate; 3 min for each of 2 conditions; standing with eyes closed on a (1) firm surface and on a (2) compliant surface (Airex balance pad, 495 406 63 mm), barefoot with feet a foot-width apart, and arms folded across the chest. The subject was asked to stand still and abstain from all movements not involved in maintaining quiet standing. | |
| ZouitaBenMoussa et al, 2009 [110] Cross Sectional Tunisia  In English/French | PA: Soccer Players Level: N/R Time Since ACLR: 8.0$\pm$0.5 months ACLR: (m=N/R), (f=); Age: 22.0$\pm$3.1 y/o Control: (m=N/R), (f=N/R); Age: 24.0$\pm$2.0 y/o ACLD: N/A | To analyse postural stability and single-leg hop’’ measurements in post-ACLR subjects and compare them with an age- and activity-matched control group. | Postural sway velocity (deg/s) | N/R | | 3 | | The testing session started with a 5-minute warm-up. Subjects were then instructed to perform several lower body flexibility exercises.  Hands free, barefoot, eyes open/closed  The assessment quantifies postural sway velocity while the athlete stands calmly on 1 foot on the force plate. The relative absence of sway in the ‘‘hold still’’ position indicates better stability. The single-leg stance assessment consisted of 4 sets of 3 trials, normally conducted in the following order: knee fully extended (left, right) (EXT)  knee flexed at 20 (left, right) (FLEX  This assessment quantifies the postural sway velocity of each leg. The sway velocity (in degrees per second) is given for all 3 trials. Subjects were allowed a 1-minute rest between tests. | |
| **Studies used Balance Platforms to assess "Standing Balance" (n=28)** | | | | | | | | | |
| Akhbari et al, 2015 [112] Cross Sectional Iran, Belgium  In English | PA: Soccer Level: N/R Time Since ACLR: 11.5$\pm$2.5 months ACLR: (m=25), (f=0); Age: 28.2$\pm$4.8 y/o Control: (m=19), (f=0); Age: 27.6$\pm$4.0 y/o ACLD: (m=23), (f=0); Age: 26.6$\pm$5.1 y/o | To assess the intra and inter-session test-retest reliability of balance and cognitive tasks under single and dual-task conditions in ACLD, ACLR and matched athletes. | Reaction time (RT), latency and amplitude from the baseline of this maximum excursion. | 500 Hz | | 3 | | Dynamic balance and cognitive tests under single- and dual-task conditions, barefoot with eyes open and close  Standing with 1 leg on a balance platform and responding to perturbation and auditory cognitive tasks under different testing conditions. | |
| Alonso et al, 2009 [113]  Cross Sectional Brazil  In English | PA: Soccer Level: Recreational Time Since ACLR: 36.0$\pm$10.0 months ACLR: (m=24), (f=0); Age: 29$\pm$6 y/o Control: (m=20), (f=0); Age: 26$\pm$6 y/o ACLD: N/A | To compare the dislocation of the center of gravity (CG) and postural balance in sedentary and recreational soccer players with and without reconstruction of the ACL using the Biodex Balance System. | Stability index (anterio-posterior, medio-lateral and general (sum of the first two)) | N/R | | 3 | | Balance on the platform on 1 leg barefoot, arms on chest.  Standing on 1 leg barefoot on the platform and trying to keep balance at different stability levels while having a feedback on the screen about the center of gravity displacement. | |
| An et al, 2015 [114] Cohort South Korea  In English | PA: N/R Level: N/R Time Since ACLR: 6.0 months (SD:N/R) ACLR: (m=10), (f=8); Age: 27.2$\pm$6.6 y/o Control: N/A ACLD: N/A | To investigate whether the proprioceptive and dynamic balancing effects of rehabilitation exercises performed after anterior cruciate ligament reconstruction are different between males and females | Change in dynamic balance functions | N/R | | N/R | | Balance on platform (Condition was not reported)  Standing on the balance platform and stabilizing in response to different stability levels. | |
| Baczkowicz et al, 2013 [115] Cross Sectional Poland  In English | PA: N/R Level: N/R Time Since ACLR: Range (11-13) months ACLR: (m=19), (f=7); Age: 28.4$\pm$6.3 y/o Control: (m=26), (f=11); Age: 27.3$\pm$5.2 y/o ACLD: N/A | To assess neuromuscular control in patients after ACL reconstruction, and in particular to determine, by evaluating dynamic balance control, to what degree deficits of afferent input affect the function of the sensorimotor system | The overall stability index (OSI)  The anterio-posterior stability index (APSI) The medio-lateral stability index (MLSI) | N/R | | 2 | | Hands down, standing barefoot on 1 leg and 2 legs with eyes open  The maintenance of the single-leg and two-leg standing position on an unstable surface was assessed | |
| Bartels et al, 2019 [116] Cohort Germany, USA  In English | PA: N/R Level: N/R Time Since ACLR: 6 months ACLR: (m=14), (f=16); Age: 31.9$\pm$12.4 y/o Control: N/A ACLD: N/A | To assess if postural stability is retained for extended periods of time following surgery. The current study is a follow-up to this previous work with the purpose of extending the postoperative testing periods: six-weeks, twelve-weeks, six-months, one-year, and two-years postoperative | Stability indicator (ST), weight distribution index (WDI), synchronization (foot coordination) and sway intensities (postural subsystems). | 32 Hz | | 1 | | With and without foam pads, eyes open and closed, head is rotated 45 degrees to the right/left or neutral, neck in flexion and extension  Postural regulation was tested at six-months, one-year and two-years post-ACLR under 8 different conditions. | |
| Denti et al, 2000 [117] Cross Sectional Italy  In English | PA: Sports  Level: Professional: 18 Amature: 32 Time Since ACLR: 73.2, (range 60-96) months ACLR: (m=43), (f=7); Age: 30.8 (SD:N/R) y/o Control: (m=29), (f=21); Age: 30.3 (SD:N/R) y/o ACLD: N/A | To investigate the long-term effect of anterior cruciate ligament (ACL) reconstruction on motor control function in the lower extremity. | Balance Index Score | 25 Hz | | 3 | | With/without visual cues  Static tests are performed either in double- or single-limb support and characterize the ability of the subject to maintain stable equilibrium without visual cues from the system. Dynamic tests, which are performed only on double-limb support, characterize motor control function as the subject displaces the platform in response to visual cues from the system. | |
| Gandolfi et al, 2018 [118] Cohort Italy  In English | PA: N/R Level: Non-competitive sports Time Since ACLR: 6.0 months (SD:N/R) ACLR: (m=29), (f=10); Age: 29.6$\pm$10.8 y/o Control: N/A ACLD: N/A | Evaluate the time course of sensorimotor integration processes involved in balance recovery during 1-year follow-up after arthroscopy and to understand whether an association exists between balance performance and semitendinosus muscle morphometric features | Percentage difference of sway (PDS) between eyes open (EO) and eyes closed (EC) | N/R | | 1 | | Barefoot, arms by side  Subject maintains standing position in different sensory conditions. The patient stands barefoot with arms alongside the body and feet in a standardized heel-to-toe position Mean magnitude of sway area (A) (mm2) with eyes open (EO) and eyes closed (EC). Each assessment was performed on a firm (floor) and a compliant surface (foam mats). Each session lasted 30 s. Body sway and sensorimotor integration processes were evaluated by computing sway area with and without vision in the 2 sensory conditions and by calculating the percentage difference of sway (PDS) between EC and EO conditions | |
| Harrison et al, 1994 [119] Cross Sectional Canada  In English | PA: N/R Level: N/R Time Since ACLR: Range (10-18) months ACLR: (m=7), (f=10); Age: 27.0$\pm$7.6 y/o Control: (m=40), (f=38); Age: 24.6$\pm$5.5 y/o ACLD: N/A | To determine whether there is a difference in single-leg standing balance between the ACL-reconstructed leg and the healthy leg. To establish the inter-rater reliability of an observational method of evaluating balance, as this is a critical concern in clinical research. | Postural sway (dispersion index) | 100 Hz | | 1 | | Single leg, arms crossed over chest  For the eyes-open tests, subjects were instructed to cross their arms over the chest with the hands-on opposite shoulders, flex the non-weight-bearing knee to 90 degrees, and fix their eyes on a stationary marking on the wall in front of them. Eyes-closed test was the same, but test started when eyes were closed | |
| Karasel et al, 2010 [120] Cross Sectional Turkey  In English | PA: Athelets 8 Non-athletes 30 Level: N/R Time Since ACLR: 16.0$\pm$9.8 months ACLR: (m=33), (f=5); Age: 27.6$\pm$6.4 y/o Control: N/A ACLD: N/A | To evaluate muscle strength, proprioception, balance, functional capacity, and activity levels of patients who received a modified accelerated rehabilitation program following ACL reconstruction with a PT graft. | Static balance index | N/A | | 3 | | A standardized warm-up for 15 minutes.  Single leg support Arms crossed over shoulders Contralateral knee is at 20 flexion  Participants were asked to keep the cursor in the middle of the screen for 30 seconds | |
| Kocak et al, 2010 [121] Cohort Turkey  In English | PA: Football (9), basketball (5), volleyball (2), handball (3), other (8) Level: 18 professionals and 9 amateurs Time Since ACLR: 6.0 months (SD:N/R) ACLR: (m=22), (f=5); Age: 26.51$\pm$8.24 y/o Control: (m=14), (f=4); Age: 20.88$\pm$3.59 y/o ACLD: N/A | To determine the functional level of activity and postural control after rehabilitation of anterior cruciate ligament reconstructed knees and compare them with non- operated limbs and healthy limbs in control subjects | Static and dynamic balance scores | N/R | | 3 | | Left/right leg standing, static/dynamic conditions  Performing one-leg standing (eyes open and closed), static (eyes open and closed) and dynamic postural control on The Kinaesthetic Ability Trainer-KAT 2000 at the 3, 6 and 12 month post- operation. | |
| Lee et al, 2019 [122] Cohort Korea  In English | PA: Soccer (48), baseball (14), basketball (25), other (9) Level: N/R Time Since ACLR: Range (6-12) months ACLR: (m=59), (f=37); Age: 29.6$\pm$9.2 y/o Control: N/A ACLD: N/A | To evaluate serial change in neuromuscular control in both operated and non-operated knees after ACLR up to 1 year postoperatively by using AT and dynamic postural stability in nonathletic patients who underwent ACLR using hamstring tendon autografts | Overall stability index (OSI) | N/R | | 2 | | Barefoot, 1 leg at a time  Dynamic single leg test; each subject stood barefoot, stood with 90° flexion of the opposite knee on the platform, arms held at the pelvis. Condition changed from most stable level to most unstable | |
| Lim et al, 2019 [123] Cohort Korea  In English | PA: N/R Level: Not professional athletes Time Since ACLR: 6.0 months (SD:N/R) ACLR: (m=19), (f=11); Age: 35.5$\pm$10.4 y/o Control: N/A ACLD: N/A | To investigate differences in improvements in isokinetic knee strength, endurance, and proprioception between patients participating in a home-based rehabilitation (HBR) or in a supervised rehabilitation (SR) exercise program. | Overall stability index (OSI) | N/R | | 2 | | The patients were positioned without shoes and socks on the BSS platform and stood on 1 foot, with the weight bearing knee in a semi-flexed position at 20◦–30◦ and the contralateral knee maintained in 90◦ flexion. The patients put their hands on their waists and maintained an upright posture with the supporting leg while focusing at the screen in front of them. | |
| Mattacola et al, 2002 [124] Cross Sectional USA  In English | PA: N/R Level: N/R Time Since ACLR: 18.0$\pm$10.0 months Dominant/Non-dominant: N/R ACLR: (m=11), (f=9); Age: 25.8$\pm$8.1 y/o Control: (m=11), (f=9); Age: 24.5$\pm$6.9 y/o ACLD: N/A | To compare postural stability, single-leg hop, and isokinetic strength measurements in subjects after ACL reconstruction with an age- and activity- matched control group | Postural stability index (anterior-posterior plane and medial-lateral plane) for single-limb and bilateral limb | N/R | | 1 | | A 5-min warm-up on stationary bicycle followed by several lower body flexibility exercises.  Single limb (right vs. left) and bilateral stance  BSS progressed from most stable to least stable level. Subjects stood with knees flexed 10-15 degrees and looked straight ahead. | |
| Mohammadi-Rad et al, 2016 [125] Cross Sectional Iran  In English | PA: N/R Level: N/R Time Since ACLR: Range (6-12) months ACLR: (m=16), (f=1); Age: 26.8$\pm$6.5 y/o Control: (m=16), (f=1); Age: 26.2$\pm$7.3 y/o ACLD: N/A | To further examine dual-tasking effect on dynamic postural stability in individuals who have undergone ACL-R and a matched control group using BBS | Anterior-posterior stability index (APSI), medial-lateral stability index (MLSI), overall stability index (OSI) | 20 Hz | | 4 | | Four conditions: stability level of 8 with eyes open/closed; stability level of 6 eyes open/closed. Performed with and without auditory Stroop test  Participants were asked to stand on the involved limb in the ACL-R group or the limb matched to the involved limb in the control group over a period of 30 seconds with eyes open and eyes closed. Participants stood barefoot with their hands placed upon the iliac crests and their unsupported foot behind the weight-bearing ankle during testing | |
| Mohammadi-Rad et al, 2012 [126] Cross Sectional Iran  In English | PA: N/R Level: N/R Time Since ACLR: 12.0$\pm6.0$ months ACLR: (m=14), (f=1); Age: 26.0$\pm$7.31 y/o Control: (m=14), (f=1); Age: 23.3$\pm$6.5 y/o ACLD: N/A | To determine the intra- and inter-session reliability of dynamic balance measures obtained using the Biodex Balance System® (BBS) for a group of athletes who had undergone ACLR and a matched control group without ACLR, while using a dual-task paradigm | Overall stability index (OSI), anterior-posterior stability index (APSI), and medial-lateral stability index (MLSI) | 20 Hz | | 4 | | Stability levels: levels 8 and 6; eyes open and closed  Participants were asked to stand on the involved limb (or in the case of the controls, the limb matched to the ACLR limb of their paired subject) on the BBS® platform with their eyes open and then closed for 30 seconds during each trial. Participants stood barefoot with both hands placed upon the iliac crests. | |
| Novaretti et al, 2018 [127] Cohort Brazil  In English | PA: N/R Level: N/R Time Since ACLR: 25.2 (range 12-52.8) months ACLR: (m=47), (f=11); Age: 34.5$\pm$11.3 y/o Control: N/A ACLD: N/A | (1) evaluate rates of return to sport after ACLR, (2) correlate 3 objective tests (isokinetic evaluation, postural stability analysis, and drop vertical jump test) completed 6 months postoperatively to return to pre-injury activity level, (3) correlate patient satisfaction and return to play after ACLR, and (4) compare quadriceps strength deficit cut-off values of 80% and 90% to return to pre-injury sport level | Postural stability analysis | N/R | | 3 | | Left and right limb  Patients were positioned at the center of the platform on a single limb. The tested limb was maintained in 10° of knee flexion, with the non-tested limb flexed and arms crossed with hands resting on the contralateral shoulder. Patients were instructed to maintain posture at the center of the platform for 20 seconds at level 4 stability testing. | |
| Ogrodzka-Ciechanowicz et al, 2018 [128] Cohort Poland  In English | PA: N/R Level: N/R Time Since ACLR: 6.0 months (SD:N/R) Dominant/Non-dominant: N/R ACLR: (m=31), (f=0); Age: 28.4$\pm$9.5 y/o Control: N/A ACLD: N/A | To evaluate effectiveness of rehabilitation in patients before and after ACLR, on the basis of stabilographic indicators. | CoP sway path an x and y axis.  CoP anterio-posterior sway path length in the y axis.  CoP medio-lateral sway path length in the x axis. | N/R | | 2 | | Standing on the right leg with eyes open (the left leg bent in the knee). Standing on the left leg with eyes open (the right leg bent in the knee).  Stood barefoot with arms along body and legs straight; focused attention on a point and asked to stand on 1 leg and bend the other leg so it wouldn't touch the ground. Patients were not allowed to connect lower limbs or support elevated leg on examined leg. | |
| Palm et al, 2015 [129]  Cross Sectional Germany  In German | PA: N/R Level: N/R Time Since ACLR: 20.0 (range 11.7-27.0) months ACLR: (m=22), (f=3); Age: 29.8$\pm$10.1 y/o Control: N/A ACLD: N/A | The aim of this work was to investigate whether through the reconstruction of the ACL if the postural control can be restored. | Overall stability index, medio-lateral stability index, anterio-posterior stability index | N/R | | 3 | | Healthy vs. injured leg; barefoot  Subjects centered foot on platform (single leg), measured in BSS Level 8 | |
| Pasquini et al, 2017 [130] Cross Sectional Italy  In English | PA: N/R Level: Amateur athletes Time Since ACLR: 12.0 months (SD:N/R) ACLR: (m=30), (f=0); Age: 28.17$\pm$7.9 y/o Control: N/A ACLD: N/A | To evaluate neuromuscular recovery in athletes who underwent ACL reconstruction with BPTB and HS autografts | Path of Center Of Mass (Y) Open Eyes  Oscillation Medio-Lateral Open Eyes  Oscillation Anterior-Posterior Open Eyes  Path of Center Of Mass (Y) Closed Eyes (Oscillation Medio-Lateral Closed Eyes  Oscillation Anterior-Posterior Closed Eyes | N/R | | N/R | | Barefoot; open vs. closed eyes; single leg  The recording was performed for a time of 30 seconds bare-foot on the stabilometric platform with arms at their sides and at the beginning with opened eyes staring a point and then with closed eyes | |
| Paterno et al, 2013 [131] Cross Sectional USA  In English | PA: Jumping, pivoting or cutting activity (Level I/II sports) Level: Competitive Time Since ACLR: 7.9$\pm$1.7 months ACLR: (m=21), (f=35); Age: 16.4$\pm$3.0 y/o Control: (m=13), (f=29); Age: 16.8$\pm$2.3 y/o ACLD: N/A | To determine if postural sway deficits during single limb stance on a dynamic, movable platform persist in subjects following ACLR and completion of rehabilitation prior to their return to sport (RTS). | Postural sway | N/R | | 3 | | Left and right limb; eyes open, shoes on  The subject was positioned and balanced centrally on a single limb in the center of the dynamic, unstable platform. The subject stood with the test limb in slight flexion (less than 10 degrees) with the contralateral limb flexed and both arms crossed | |
| Pinheiro et al, 2010 [132] Cross Sectional Portugal  In French | PA: N/R Level: N/R Time Since ACLR: 6.19$\pm$3.43 months ACLR: (m=18), (f=13); Age: 28.9$\pm$5.63 y/o Control: (m=15), (f=16); Age: 25.6$\pm$5.8 y/o ACLD: N/A | To compare postural stability in healthy subjects and patients operated on for ACL reconstruction 2 to 5 years ago. | Total instability index, anterio-posterior stability index, medio-lateral stability index | N/R | | 3 | | Left vs. right limb  Positioned center of foot on platform; supporting limb is slightly flexed, arms crossed, maintain balance for 20 sec | |
| Plocki et al, 2018 [133] Cross Sectional Poland  In English | PA: N/R Level: N/R Time Since ACLR: Range (36-48) months ACLR: (m=39), (f=13); Age: 34.6$\pm$7.3 y/o Control: N/A ACLD: N/A | To compare the postural stability in patients after ACL reconstruction with LARS and autogenous graft | CoP path length, mean CoP deviation in anterio-posterior and lateral direction, Mean CoP velocity in AP and L direction, CoP path length area plotted, distribution of load for healthy lower limb and lower limb after reconstruction | N/R | | 2 | | With double-leg stance: the Romberg test with open eyes and the Romberg test with eyes closed to assess postural stability and the test to assess the distribution of loads with eyes open.  Three tests were performed in the double- leg stance position: the Romberg test with open eyes and the Romberg test with eyes closed to assess postural stability and the test to assess the distribution of loads with eyes open. The tests lasted 30 seconds. | |
| Risberg et al, 2007 [134] RCT Norway  In English | PA: N/R Level: N/R Time Since ACLR: 6.0 months (SD:N/R) ACLR: (m=47), (f=27); Age: 28.4 (range 16.7-40.3) y/o Control: N/A ACLD: N/A | To determine the effect of an NT program versus a traditional ST program on knee function (Cincinnati Knee Score) following ACL reconstruction. A secondary aim was to evaluate the effect on muscle strength, other patient-related out- come measures (visual analog scale [VAS] and 36-Item Short-Form Health Survey [SF-36]),17 pain, functional performance (hop tests), proprioception, and balance. | Balance index (static - uninvolved leg, static - involved leg, dynamic) | N/R | | 3 | | Static and dynamic balance; involved vs. uninvolved limb  Each subject completed a 1-leg static balance test on each leg (3 trials on each leg) and a 2-leg dynamic test (3 trials). The position of the feet was recorded, and the same position was identified at the follow-up tests. | |
| Shiraishi et al, 1996 [135] Cross Sectional Japan  In English | PA: N/R Level: N/R Time Since ACLR: 2.1$\pm$1.8 months ACLR: (m=22), (f=31); Age: 21.5$\pm$6.4 y/o Control: (m=15), (f=15); Age: 22.7$\pm$2.9 y/o ACLD: (m=15), (f=15); Age: 23.7$\pm$5.3 y/o | To examine the hypotheses that anterior cruciate ligament (ACL) reconstruction improves the proprioception of the knee beyond the level of ACLD knees, and that proprioception of the knee correlates well with knee function after ACL reconstruction. | Length of the movement of CoP | N/R | | 3 | | Single leg Knee flexed to 20 degrees Contralateral knee flexed to 90  Standing on 1 leg and looking at a fixed mark for 20 seconds | |
| Unver et al, 2005 [136] Cohort Turkey  In Turkish | PA: Sports Level: Recreational Time Since ACLR: Range (6-12) months ACLR: (m=10), (f=0); Age: 29.4$\pm$6.6 y/o Control: (m=10), (f=0); Age: 29.4$\pm$6.6 y/o ACLD: N/A | To determine the functional level of activity and postural control of anterior cruciate ligaments reconstructed patients following rehabilitation and to compare the results with healthy individuals. | Balance Score Index | N/R | | 3 | | Balance was assessed at difficulty level 6  Arms crossed around chest Focus point is at 180 cm high, and 130 cm away  Static: Maintain the position on each leg for 30 seconds while focusing on the focus point  Dynamic: Standing on both legs, and move a ball using the platform 360 degrees within 30 seconds. | |
| Vathrakokilis et al, 2008 [137] Exerimental Greece  In English | PA: Athletes Level: Compititive  Time Since ACLR: 22 (range 8-30) months ACLR: (m=17), (f=7); Age: 28.6$\pm$6.1 y/o Control: N/A ACLD: N/A | To assess the influence of a balance-training program on knee joint proprioception, between ACLR patients who had a lack of proprioceptive ability on the reconstructed limb in relation to the healthy one and they had undergone ACLR at a mean of 22 months (range 8–30) before the initiation of the study. | Stability (time) in sagittal plane. Dynamic stability (time) in sagittal plane.  Stability (time) in frontal plane. Dynamic stability (time) in frontal plane.  Stability (time) in all directions. Over all Stability index AP Stability index ML Stability index | N/R | | 3 | | Balance evaluations in the current study made at stability level 2 on the electronic stability system, for both legs (injured and healthy). Balance ability was assessed in all subjects at baseline and after the completion of the 8-week balance program. All the participants were instructed to focus on the visual feedback screen directly in front of them and to maintain the cursor at the center of the bull’s-eye on the screen. They performed three 20 sec trials out of which only the best score was recorded | |
| Wrzesien et al, 2019 [138] Cross Sectional Poland  In English | PA: basketball players Level: Professional Time Since ACLR: 38.4$\pm$14.8 months ACLR: (m=0), (f=10); Age: 23.3$\pm$3.9 y/o Control: (m=0), (f=10); Age: 22.0$\pm$4.1 y/o ACLD: N/A | To assess muscle strength, postural stability and quality of movement patterns according to the FMS method in female basketball players after surgical reconstruction of the ACL who returned to professional sports. | Overall Stability Index ML Stability Index AP Stability Index | N/R | | 3 | | 10-min warm-up consisted of the following exercise;  a) two series of eight repetitions in the pattern of both feet squat with the load equal to the body mass of the subject, b) two series of eight repetitions in the pattern of one foot squat with the load equal to 1/2 of the body mass of the subject, c) two series of eight repetitions in the pattern of one foot squat with the load equal to 3/4 of the body mass of the subject.  The subjects did 1 leg stance on dynamo-graphic platform. The protocol consisted of 3 trials lasting 30 seconds each, with 10-second break, without changing body position. There were 3 trials on stable surface and 3 trials on unstable surface, whose degree of stability was 4.  We restricted the view of the cursor on the screen of the platform so that the subjects could not correct the setting of the base of the platform. | |
| Zallinger et al, 2004 [139] Cohort Austria  In German | PA: N/R Level: N/R Time Since ACLR: Range (6-12) months ACLR: Sex: N/R; Age: N/R Control: N/A ACLD: N/A | To assess knee stability after rehabilitation post ACLR | Stability Index | N/R | | N/R | | N/R | |
| **Studies used Wii Balance Boards to assess "Standing Balance" (n=4)** | | | | | | | | | |
| Clark et al, 2017 [140] Cross Sectional Australia  In English | PA: N/R Level: N/R Time Since ACLR: 12.8 (range 10.1-18.0) months ACLR: (m=234), (f=180); Age: 27.8$\pm$10.3 y/o Control: N/A ACLD: N/A | To examine inter-limb and sex differences during single leg-standing balance in a large cohort of patients 1 year post-ACLR.   To examine whether static single leg balance was associated with demographic or physical function variables, as this has not been well elucidated in a large cohort of patients and has implications for the importance of implementing this testing protocol. | Range of AP & ML displacement of the CoP Path length in the AP & ML of the CoP Fast sway and slow sway in AP & ML directions of the CoP | N/A | | | N/R | Eyes open  Participants stood with a barefoot on a Nintendo Wii Balance Board (WBB) (Nintendo, Japan), with the longitudinal axis of the foot positioned on the long axis of the board, and aligned with the centre of the board. They were instructed to remain as still as possible throughout the test and the limbs were tested sequentially and independently. This technique has been validated against typical laboratory force platforms in numerous studies | |
| Clark et al, 2014 [141]  Retrospective Cohort Australia  In English | PA: The majority play sport Level: Recreational  Time Since ACLR: 10.7$\pm$4.3 months ACLR: (m=30), (f=15); Age: 26.0$\pm$9.8 y/o Control: (m=30), (f=15); Age: 26.4$\pm$9.8 y/o ACLD: N/A | To assess balance using traditional, wavelet and signal irregularity based measures in a group of ACLR and matched control subjects. | CoP path velocity CoP amplitude (AP) CoP standard deviation (SD), Moderate frequency (cm/s) Low frequency (cm/s) Very low frequency (cm/s) Ultra low frequency (cm/s) Sample entropy | N/A | | | 3 | Participants stood barefooted on the WBB on 1 leg, with the middle of the longitudinal axis of their foot aligned with a line showing the centre of the WBB in the AP plane. They were instructed to stand as still as possible for 30 s on their ACLR limb or matched limb for the control group. Specific positioning regarding the weight bearing knee (slightly flexed to aproximately 20), non-weight bearing knee (flexed to 90), non-weight bearing hip (neutral flexion/extension) and hands (on hips) was adjusted on the basis of visual observation by the investigator. Participants fixed their gaze on a white dot displayed on a computer monitor positioned at eye-level 1.4 m from the WBB. | |
| Culvenor et al, 2016 [142] Cross Sectional Australia  In English | PA: N/R Level: N/R Time Since ACLR: 13.0$\pm$1.0 months ACLR: (m=66), (f=31); Age: Median (IQR): 28 (23-35) y/o Control: (m=20), (f=28); Age: Median (IQR): 30 (25-34) y/o ACLD: N/A | To determine whether dynamic postural control during a single-leg squat is impaired following ACLR compared with the uninjured contralateral limb and with healthy controls.   To evaluate the relationship between dynamic postural control and self-reported and objective function in the ACLR group. | CoP path velocity, cm/s ML range, cm ML SD, cm AP range, cm AP SD, cm | N/A | | | 5 | Eyes open. Arms crossed on chest. Barefoot  From the starting position of full knee extension in single-leg stance, participants were instructed to squat until their buttocks just touched the plinth (placed at 60 cm high behind the participant), then return to the starting position. This was repeated 5 times, and no specific instructions were provided regarding trunk position or movement. A metronome was used to control the speed of the 5 repetitions (2 seconds lowering, 2 seconds rising). The non– weight-bearing leg was held in hip flexion and knee extension, so that the foot was positioned in front of the body and just off the ground. | |
| Howells et al, 2013 [143] Cross Sectional Australia  In English | PA: Sports Level: (4–7 days/week) 9  (1–3 days/week) 25 (1–3 times/month) 2  (no sports) 9 Time Since ACLR: 10.7$\pm$4.3 months ACLR: (m=30), (f=15); Age: 26.0$\pm$9.8 y/o Control: (m=30), (f=15); Age: 26.4$\pm$9.8 y/o ACLD: N/A | To compare postural control, with and without a secondary task that required controlled movement of the upper limb, in patients following ACL reconstruction with a control group. | CoP path AP CoP path ML CoP path total | N/R | | | 3 | Barefoot. Knee of supporting knee at 20, other knee 90 flexion, hands on hips or (1 arm performing abd/adduction), gazing on a white dot placed 1.4 me at eye level  Standing on 1 leg for 30 seconds while holding an accelerometer in the hand on the contralateral side to the supporting leg. Participants were asked to abduct the shoulder through the full ROM while the elbow is extended. The accelerometer movement resulted in the movement of a red marker on the computer screen. Participants were instructed to match this red ‘angle’ marker to the movement of a larger yellow ‘target’ marker that moved vertically up and down on a sliding scale. This target marker was controlled by a sine wave oscillating at 0.33 Hz, resulting in 10 full adduction/abduction cycles per 30s trial. Participants were reminded that the aim of the test was to ‘stand as still as possible. | |
| **Studies used Pressure Mats to assess "Standing Balance" (n=2)** | | | | | | | | |  |
| Chaves et al, 2012 [144] Cross Sectional Brazil  In English | PA: Soccer Level: Professional Time Since ACLR: Range (4-12) months  ACLR: (m=22), (f=0); Age: 21.8$\pm$4.4 y/o Control: N/A ACLD: N/A | To evaluate the neuromuscular efficiency of the vastus medialis oblique (VMO) and postural balance in high-performance soccer athletes after anterior cruciate ligament (ACL) reconstruction, between 4 and 12 month post-operation, comparing to the unaffected limb. | Ellipse surface | N/R | | | 2 | For the bi-podalic evaluation, the athlete was instructed to remain in a standing position with feet put into a triangular shape, which accompanies the appliance, at an external rotation of 15º, along with arms at their sides, gaze directed to the horizon, and keep their temporomadibular joint relaxed (open mouth) for 51 seconds. For the mono-podalic test, the individuals were supported by their left foot, keeping the right foot elevated with the knee flexed and then reversed, holding each position for 5 seconds. All tests were performed twice, first with open eyes and the second with closed eyes. | |
| Kouvelioti et al, 2015 [145] Cross Sectional Greece  In English | PA: Basketball, handball, soccer, volleyball Level: N/R Time Since ACLR: Median 24 months (IQR:N/R)  ACLR: Sex: N/R; Age: 24.4$\pm$3.5 y/o Control: Sex: N/R; Age: 26.7$\pm$2.4 y/o ACLD: N/A | To examine the test– retest reliability of balance variables measured in double-leg and single-limb stance in subjects who underwent ACL reconstruction and controls | Sway ellipse, SD of CoP in x and y directions, total center of pressure path, center of pressure velocity, sway area | 25 Hz | | | 3 | Double leg balance test, right and left leg balance test; barefoot  Subjects stood erect, as motionless as possible, eyes looking straight ahead, feet shoulder width apart at arms at sides. Instructed to keep quiet stance posture for 30s | |
| *N/R* Not reported, *N/A* Not applicable, *PA* Physical activity, *Level* Activity level, *SD* standard deviation, *IQR* Interquartile range, *ACLR* Anterior cruciate ligament reconstruction, *ACLD* anterior cruciate ligament deficient, *CoP* center of pressure, *AP* anterior posterior, *ML* medial lateral, *DPSI* Dynamic postural sway index, *TTB* Time to balance. | | | | | | | | | |

Table 3 Data Extraction Table for Studying Assessing Gait

| **Study Characteristic**  **(author, year, design, country, language)** | **Sample Characteristic**  **(Physical Activity (PA), level, time since surgery, side of surgery, sample size by sex, age)** | **Study Objectives** | **Parameters** | | **Sampling Frequency** | **Number of Repetitions** | - **Testing Condition or Challenges** - **Protocol Summery** |
| --- | --- | --- | --- | --- | --- | --- | --- |
| **Studies used Force Plates to assess "Gait" (n=26)** | | | | | | | |
| Azus et al, 2018[166] Cohort USA  In English | PA: N/R Level: N/R Time Since ACLR: 6.0 months (SD:N/R)  ACLR: (m=25), (f=20); Age: 29.6$\pm$8.5 y/o Control: N/A ACLD: N/A | To evaluate the correlations between gait patterns and Knee injury and Osteoarthritis Outcome Score (KOOS) survey data at 3 time points (pre-surgery, six- and twelve-month post-surgery) in subjects with ACL injury and reconstruction. | GRF | 1000 Hz | | 4 | Walking a successful trial the foot of the tested limb was within borders of either of the force plates from initial contact to toe-off |
| Blackburn et al, 2016 [164] Cross Sectional USA  In English | PA: N/R Level: Exercise at least 30 minutes 3 times per week (Level N/R) Time Since ACLR: 49.0$\pm$39.0 months ACLR: (m=11), (f=28); Age: 22.0$\pm$3.0 y/o Control: N/A ACLD: N/A | To examine relationships between several indices of quadriceps function and gait biomechanics linked to knee OA development in individuals with ACLR. | Peak vGRF  vGRF Linear Loading Rate vGRF Instantaneous Loading Rate Heel-strike Transient Heel-strike Transient Linear Loading Rate Heel-strike Transient Instantaneous Loading Rate | 1200 Hz | | 5 | Barefoot  Patients were asked to walk 5 times on a 7m walkway that has force plates embedded in it. |
| Blackburn et al, 2016 [146] Cross Sectional USA  In English | PA: N/R Level: N/R Time Since ACLR: 46.2$\pm$39.7 months ACLR: (m=0), (f=29); Age: 21.7$\pm$3.1 y/o Control: N/A ACLD: N/A | To compare loading characteristics between ACLR and contralateral limbs  To compare loading characteristics between individuals who consistently displayed heel-strike transient (HST) during gait and those who did not using the classification schema developed by Radin et al. (1986) to determine the validity of the HST as an indicator of impulsive loading. | Peak vGRF (xBW) Linear Loading Rate (xBW/s) Instantaneous Loading Rate (xBW/s) | 1200 Hz | | 5 | Subjects translated at least 3m barefoot via 3-5 steps (at self-selected speed) prior to contact with the first force plate, and completed at least 2 steps following contact with the second force plate |
| Blackburn et al, 2020 [147] Cross Sectional USA  In English | PA: N/R Level: Exercise regularly for 20 minutes 3/week. (Level N/R) Time Since ACLR: 27$\pm$16 months ACLR: (m=20), (f=52); Age: 21$.0\pm$3.0 y/o Control: N/A ACLD: N/A | to compare somatosensory function (proprioception and vibratory perception) in the ACLR limb to the contralateral limb, and to evaluate associations between somatosensory function and gait biomechanics previously linked to PTOA development | Peak vGRF vGRF Loading Rate | 1200 Hz | | 5 | At least 5 practice trials were performed to determine the average preferred gait speed and ensure subjects could consistently strike the force plates without noticeably altering their gait |
| Blackburn et al, 2019 [148] Retrospective Cohort USA  In English | PA: N/R Level: Exercise regularly for 20 minutes 3/week. (Level N/R) Time Since ACLR: 27$\pm$15 months ACLR: (m=15), (f=35); Age: 20.0$\pm$3.0 y/o Control: (m=19), (f=6); Age: 20$\pm$1.0 y/o ACLD: N/A | To compare co-activation during walking between the ACLR and contralateral limbs, as well as healthy control subjects.   to evaluate relationships between co-activation and gait biomechanics in individuals with ACLR | Peak vGRF vGRF Linear Loading Rate vGRF Instantaneous Loading Rate # of trials Heel-strike Transient | 1200 Hz | | 5 | At least 5 practice trials were performed to determine the average preferred gait speed and ensure subjects could consistently strike the force plates without noticeably altering their gait |
| Bulgheroni et al, 1997 [149] Cross sectional Italy  In English | PA: N/R Level: N/R Time Since ACLR: 17.0$\pm$5.0 months ACLR: (m=15), (f=0); Age: 27.0$\pm6.0$ y/o Control: (m=5), (f=0); Age: 28.0$\pm3.0$ y/o ACLD: (m=10), (f=0); Age: 25.0$\pm3.0$ y/o | To analyze the changes in select gait parameters following anterior cruciate ligament (ACL) reconstruction | GRF | 500 Hz | | 5 | Each subject was asked to perform at least 5 trials of walking at his natural cadence. A 20-m distance was used to allow the subject to reach a steady state of walking. |
| Colne et al, 2006 [167] Cross Sectional France  In English | PA: The majority play sport Level: Recreational  Time Since ACLR: 11.0$\pm$12.0 months ACLR: (m=N/R), (f=); Age: 27.0$\pm$8.0 y/o Control: (m=N/R), (f=N/R); Age: 29$\pm$11.0 y/o ACLD: (m=N/R), (f=N/R); Age: 38$\pm$10.0 y/o | To study the dynamics of balance recovery and the muscular activities after a forward fall in patients presenting an ACL lesion, and in control subjects. | Balance recovery duration. Toe-off latency. Heel-off latency. Swing phase duration.  Braking time. Velocity of the step. Negative peak of the vertical acceleration of the (CoG) before toe-off of the swing limb. Positive peak of the vertical acceleration of the reaction phase (before toe-off). Vertical velocity of the CoG at heel contact. Variation of the height of the CoG at heel contact. Step Length. | 500 Hz | | 10 | The subject stands on a force plate in a forward inclined posture at 15 degree. The body is straight, the arms hang alongside the body and the eyes look straight ahead. The subject is held by a restraining device composed of an abdominal belt and a horizontal steel cable connected to an electromagnet mounted on a dynamometer. The device is released, without the subject’s knowledge, causing the subject to fall forward. The subject is instructed to take a few steps to recover balance. Participants were instructed to move forward with either the injured side or the healthy side. |
| Johnston et al, 2019 [150] Cross Sectional USA  In English | PA: N/R Level: N/R Time Since ACLR: 27.1$\pm$27.9 months ACLR: (m=34), (f=64); Age: 21.8$\pm$3.2 y/o Control: N/A ACLD: N/A | To compare walking gait biomechanics previously implicated in the mechanical pathogenesis of knee osteoarthritis between individuals with HT and PT grafts. | Peak vGRF vGRF loading rate | 1200 Hz | | 5 | Participants were asked to walk barefoot at their self-selected speed on a walkway with embedded force plates. |
| Lim et al, 2015 [168] Cohort South Korea  In English | PA: N/R Level: N/R Time Since ACLR: 6.0 months (SD:N/R) ACLR: (m=N/R), (f=); Age: 31.6$\pm$7.0 y/o Control: (m=N/R), (f=); Age: 33.4$\pm$6.0 y/o ACLD: N/A | To compare the early functional recovery using biomechanical properties between ACL- and PCL-reconstructed patients and to determine the biomechanical deficit of PCL-reconstructed patients compared to ACL-reconstructed patients | GRF | 1200 Hz | | 3 | 5-min warm up (no details)  Participants were required to perform a cutting 45°, 90°, 135°, and 180° turn walking, and 180° turn running task along the laboratory gateway. |
| Luc-Harkey et al, 2016 [151] Cross Sectional USA  In English | PA: Sports Level: Recreational Time Since ACLR: 43.5$\pm$37.7 months ACLR: (m=12), (f=29); Age: 21.8$\pm$3.2 y/o Control: N/A ACLD: N/A | To determine if involved limb sagittal plane knee kinematics (knee flexion angle at heelstrike, peak knee flexion angle, knee flexion excursion) predict kinetics (peak vGRF, vGRF loading rate) in the involved limb of individuals with ACLr during walking gait. Additionally, in order to determine if inter-limb differences in the selected kinematics predict inter-limb differences in kinetics, we sought to determine if kinematic limb symmetry index LSI) is predictive of kinetic LSI in individuals with ACLr during walking gait. | Peak vGRF normalized to body weight Instantaneous vGRF loading rate Linear vGRF loading rate  vGRF LSI | 1200 Hz | | 5 | Barefoot wearing tight fitting spandex shorts  Five walking gait trials were then collected and were considered acceptable for data analysis if 1) both feet individually struck a single force plate, 2) participants maintained a forward eye gaze and did not aim for the force plates, 3) gait speed was within ±5% of the average speed determined during practice trials, and 4) gait kinematics were not visibly altered during the trial (e.g. trip or stutter step). |
| Mantashloo et al, 2020 [152] Cross Sectional Iran  In English | PA: N/R Level: N/R Time Since ACLR: More than 6 months ACLR: (m=28), (f=0); Age: 23.7$\pm$2.0 y/o Control: (m=28), (f=0); Age: 24.6$\pm$2.4 y/o ACLD: N/A | To examine the symmetry of vGRF (frst and second peak) and selected knee muscle (Gastrocnemius (GC), rectus femoris(RF), and biceps femoris(BF)) activity in male subjects with and without unilateral ACL reconstruction gait cycles | First and second peaks of vGRF Phase related SI of vGRF | 500 Hz | | 3 | Warm-up was given (no details)  The subjects were asked to cross an 8 m path through the force plate. Subjects needed to place both feet on the force plate and cross it. They were asked to walk normally; if any of the legs were not completely on the force plate, the test was repeated. The tests were repeated long enough to obtain 3hree correct tests |
| Milandri et al, 2017 [153] Cross Sectional South Africa  In English | PA: N/R Level: N/R Time Since ACLR: Approx. 60 months ACLR: (m=15), (f=0); Age: 37.4$\pm$10.7 y/o Control: (m=15), (f=0); Age: 28.6$\pm$6.8 y/o ACLD: N/A | To investigate biomechanics in males long after ACL-reconstruction during both common conditions of walking and running.  To address gap in the literature for males with primary ACL injury and links to biomechanical deviations, and for tests after ACL- reconstruction. To better understand chronic joint loading in males long after ACL-reconstruction surgery | vGRF | N/R | | 5 | Participants were requested to perform straight-line barefoot walking and jogging at self-selected speeds, to allow recording of natural level-ground gait |
| Moya-Angeler et al, 2017 [83] Cohort USA  In English | PA: N/R Level: N/R Time Since ACLR: 6.0 months (SD:N/R) ACLR: (m=74), (f=0); Age: 34.0$\pm$9.0 y/o Control: N/A ACLD: N/A | To evaluate the functional status prior to and at different times after ACLR, and to analyze the changes in the kinetic patterns of the involved and uninvolved limb lower during gait, sprint and 3 hop tests | Gait: anterior-posterior shifting point (APSP), heel maximum vertical force (MVF), single-limb MVF, impulse MVF, maximum anterior force (MAF) and maximum posterior force (MPF)  Sprint: MVF | N/R | | 3 | All activities performed on force plates.  Gait: 5-m walkway with force plates embedded, walk at self-selected comfrotable pace.   Sprint: patient started standing on both platforms, instructed to sprint as fast as possible for 5s  Single-leg hop: stand on 1 leg, hop as far forward as possible  Drop vertical jump: dropped off 30 cm box and performed maximal jump after landing  Vertical hop test: begin standing on both platforms, hop using arms as countermovement |
| Perraton et al, 2018 [154] Cross Sectional Australia  In English | PA: Level I/II sports Level: N/R Time Since ACLR: 16.5$\pm$3 months ACLR: (m=38), (f=23); Age: 28.5$\pm$6.5 y/o Control: N/A ACLD: N/A | To compare knee joint moments measured during overland running of individuals with satisfactory and poor knee function—i.e. self-reported knee function and/or hop tests. The secondary aim was to compare sagittal plane knee kinematics, quadriceps strength and vertical ground reaction force (vGRF) of both groups to help explain any differences in knee joint moments observed between groups. | Peak vGRF (per kg body mass) | 1080 Hz | | 3 | Shoes on.  Participants were instructed to look forward (to avoid targeting force plates) and run at a comfortable speed. To increase external generalisability, running speed was not constrained. Participants wore Nike Straprunner IV running sandals (Nike, Beaverton, US). Three trials involving a complete foot strike on a single force plate were acquired for each participant. At the end of the running trials, partici- pants were asked whether they experienced knee pain during running (yes/no). |
| Pfeiffer et al, 2018 [155] Cross Sectional USA  In English | PA: N/R Level: N/R Time Since ACLR: 49.6$\pm$40.6 months ACLR: (m=9), (f=26); Age: 22.1$\pm$3.4 y/o Control: N/A ACLD: N/A | The primary purpose of this study was to determine if individuals with a unilateral ACLR, who demonstrate greater peak kinematic and kinetic magnitudes in the ACLR and uninjured limb during walking gait also demonstrate greater peak kinematic and kinetic magnitudes in each limb during jump-landing. Additionally, we will determine if those who demonstrate greater kinematic and kinetic asymmetries during walking gait also demonstrate greater asymmetries during jump-landing using limb symmetry indices (LSI). | Instantaneous loading rate, linear loading rate | 1200 Hz | | 5 | Gait: all participants in this cohort completed all walking trials barefoot. During all walking gait trials, participants were instructed to walk at a self-selected speed over 2 force plates embedded in a staggered formation towards the middle of a 6m walkway so that the entire stance phase for both limbs could be collected during a single trial  Jump landing: All participants wore their own athletic footwear for the jump-landing trials. Participants performed jump- landing from a 30cm box positioned 50% of the participant’s height from the front edge of the force plates on each force plate, and immediately jump vertically as high as possible |
| Pietrosimone et al, 2016 [156] Cross Sectional USA  In English | PA: N/R Level: N/R Time Since ACLR: 43.2$\pm$36.4 months ACLR: (m=9), (f=11); Age: 22.0$\pm$3.6 y/o Control: N/A ACLD: N/A | The primary purpose of the current study was to determine if habitual walking speed, recorded in a motion analysis laboratory, associates with serum biomarkers of collagen and proteoglycan breakdown in individuals with an ACLR. Identifying relationships between walking speed and biomarkers of collagen and proteoglycan breakdown would be an initial step in determining whether walking speed may be a useful clinical indicator of post- traumatic OA development in individuals with an ACLR | Peak vGRF and peak vGRF loading rate | 1200 Hz | | 5 | Participants were instructed to walk barefoot at a self-selected speed described as “comfortably walking over a sidewalk.” Participants were instructed to focus on an X marked on the wall in the laboratory and walk across the entire 6-meter capture volume. |
| Pietrosimone et al, 2016 [157] Cross Sectional USA  In English | PA: N/R Level: N/R Time Since ACLR: 37.9$\pm$29.3 months ACLR: (m=8), (f=11); Age: 21.6$\pm$3.4 y/o Control: N/A ACLD: N/A | To explore the associations between peak vGRF and vGRF loading rate and serum biomarkers of collagen breakdown (collagen type II cleavage product [C2C]), collagen synthesis (collagen type II C-propeptide [CPII]), collagen degradation: synthesis ratios (collagen breakdown: collagen synthesis [C2C:CPII]), and proteogly- can breakdown (aggrecan) in the injured and uninjured limb of individuals with ACLR. | peak vGRF, peak vGRF, loading rate | 1000 Hz | | 5 | Participants were instructed to walk barefoot at a self-selected speed over 2 force plates embedded in a 6-m walkway. The 2 force plates were staggered such that the entire stance phase for both the right and left limbs could be collected from a single trial. Participants were instructed to look straight ahead and maintain a constant speed |
| Rudroff et al, 2003 [65] Cross Sectional Germany  In English | PA: Soccer Level: N/R Time Since ACLR: 24.0 months (SD:N/R) ACLR: (m=30), (f=0); Age: 30.9$\pm$5.4 y/o Control: (m=10), (f=0); Age: 31.1$\pm$4.7 y/o ACLD: N/A | To compare the clinical outcome of ACL reconstruction using the four-strand hamstring tendon autografts and ACL reconstruction using the patellar tendon graft 2 yr. after surgery. | Vertical jump-off force, first vertical maximum | 1000 Hz | | Squats: 5 trials; gait, one- and two-legged jumps: 6 trials | Gait: walk barefoot over 2 force plates 6 times; data of last 5 trials used |
| Schliemann et al, 2018 [158] Cohort  Germany  In English | PA: N/R Level: N/R Time Since ACLR: Range (6-12) months ACLR: (m=22), (f=8); Age: 29.1$\pm$12.0 y/o Control: N/A ACLD: N/A | To compare the early functional results after DIS with those after ACL reconstruction in a prospective randomized study. | vGRF | 600 Hz | | N/R | Patients walked at self-selected speed across the force plates and repeated trials were stored for further analyses |
| Schmalz et al, 1998 [159] Cohort Germany  In German | PA: Athletes Level: Recreational  Time Since ACLR: Range (6-12) months ACLR: Sex: N/R; Age: 29.0$\pm$6.0 y/o Control: Sex: N/R; Age: 28.0$\pm$5.0 y/o ACLD: N/A | To evaluate the rehabiliation of a group of patients with patellar tendon autograft reconstructed knees by means of gait parameters in the first postoperative year | GRF | 400 Hz | | 12-Oct | Participants walked at a fast speed 10-12 times, and the GRF was measured. |
| Shimizu et al, 2020 [69] Cohort USA, Japan  In English | PA: N/R Level: N/R Time Since ACLR: Range (6-36) months ACLR: (m=20), (f=16); Age: 31.5$\pm$7.6 y/o Control: (m=9), (f=5); Age: 31.4$\pm$4.9 y/o ACLD: N/A | (1) To investigate the longitudinal changes in meniscal T1r/T2 values and biomechanics during gait and landing tasks after ACLR   (2) To investigate the associations between changes in meniscal composition using T1r/T2 mapping and biomechanics in patients with ACLR. | Peak vGRF | 1000 Hz | | 3 | Participants were instructed to walk at a controlled speed of 1.35 m/s. with shoes on |
| Sritharan et al, 2020 [165] Cross Sectional Australia  In English | PA: N/R Level: Moderate Time Since ACLR: 17.0$\pm$3.0 months ACLR: (m=33), (f=22); Age: 28.0$\pm$7.0 y/o Control: N/A ACLD: N/A | To determine whether impairments in lower limb biomechanics during running are evident in the ACLR limb, compared with the uninjured limb, at 12 to 24 months after ACLR. | vGRF AP GRF | 1080 Hz | | 3 | Running trials were repeated until 3 trials involving a complete foot-strike on a single force plate were acquired for each leg for every with shoes on |
| Teng et al, 2017 [160] Cohort USA  In English | PA: N/R Level: N/R Time Since ACLR: Range (6-12) months ACLR: (m=20), (f=13); Age: 30.6$\pm$8.6 y/o Control: (m=8), (f=4); Age: 31.7$\pm$5.5 y/o ACLD: N/A | The primary purpose of this study was to examine whether gait characteristics (ie, peak KFM, KFA, and vGRF) observed before and 6 months and 1 year after ACLR are associated with prospective changes in MTFJ cartilage T1r and T2 at 6 months, 1 year, and 2 years after ACLR.  The secondary purpose was to compare gait characteristics (ie, peak KFM, KFA, and vGRF) observed before and 6 months and 1 year after ACLR to those of healthy controls. | Peak vGRF | 1000 | | 3 | Participants were instructed to walk at a controlled speed of 1.3 m/s. A trial was considered successful when the foot of the tested limb fell within the borders of the force platform from initial contact to toe-off and the speed was within 65% of the target speed. |
| Webster et al, 2012 [161] Cross Sectional Australia  In English | PA: N/R Level: N/R Time Since ACLR: 10.3$\pm$2.7 months ACLR: (m=32), (f=0); Age: 25.7$\pm$6.2 y/o Control: (m=32), (f=0); Age: 25.0$\pm$5.0 y/o ACLD: N/A | To compare the knee adduction moment recorded during level gait between a group of patients with patellar tendon ACL reconstruction, a group with hamstring tendon ACL reconstruction and a control comparison group. | vGRF | N/R | | 3 | Subjects were asked to walk barefooted up and down the walkway several times at their own pace until they were relaxed and accustomed to the markers. This also enabled a starting point to be identified so that the subject would contact the force plate in normal stride. Subjects were then asked to complete a number of walks at their self-selected comfortable speed whilst data were collected. They were not aware of the presence of the force plates until data collected was completed. Data collection continued until a minimum of 3 trials with good force plate contact was recorded for both left and right limbs. |
| Webster et al, 2012 [162] Cohort Australia  In English | PA: N/R Level: N/R Time Since ACLR: Range (10.0$\pm$2.0 to 39.6$\pm$4.8) months ACLR: (m=13), (f=3); Age: 26.0$\pm$6.0 y/o Control: N/A ACLD: N/A | To conduct a longitudinal gait study in a group of patients who had undergone ACL reconstruction surgery in order to examine the extent to which gait patterns at an early initial assessment (within 12 months of surgery) are maintained or changed at follow-up of greater than 3 years after surgery. | vGRF | N/R | | 3 | Subjects were asked to walk barefooted up and down the walkway several times at their own pace until they were relaxed and accustomed to the markers. This also enabled a starting point to be identified so that the subject would contact the force plate in normal stride. Subjects were then asked to complete a number of walks at their self-selected comfortable speed whilst data were collected. They were not aware of the presence of the force plates until data collected was completed. Data collection continued until a minimum of 3 trials with good force plate contact was recorded for both left and right limbs. |
| Wellsandt et al, 2017 [163] Cross Sectional USA  In English | PA: Soccer, basketball, skiing and tennis Level: 17 level 1 13 level 2 Time Since ACLR: 6.7$\pm$0.7 months ACLR: (m=19), (f=11); Age: 30.5$\pm$11.1 y/o Control: N/A ACLD: N/A | To determine if ground reaction forces, knee joint moments, and muscle co-contraction predict knee joint contact forces 6 months after ACL reconstruction. | vGRF | 1080 | | 3 | Patients walked at a self-selected speed which was maintained (5%) throughout the testing session. |
| **Studies used Force Measuring Treadmills to assess "Gait" (n=6)** | | | | | | | |
| Evans-Pickett et al, 2020 [169] Cross-Over USA  In English | PA: N/R Level: N/R Time Since ACLR: 9.0$\pm$1.4 months ACLR: (m=8), (f=4); Age: 20.5$\pm$3.8 y/o Control: N/A ACLD: N/A | to evaluate the effects of modifying the vGRF impact peak of stance on the stance waveforms of 4 lower extremity biomechanical variables associated with post-traumatic OA development: (i.e. vGRF, knee flexion angle, internal knee extension moment, and knee abduction moment). | vGRF | 1200 Hz | | N/A ( GRF was measured in the last 5 minutes of walking) | Walking on the treadmil on a predetermined walking speed. |
| Goetschius et al, 2018 [170] Cross Sectional USA  In English | PA: N/R Level: N/R Time Since ACLR: 52.7$\pm$14.9 months ACLR: (m=17), (f=39); Age: 22.9$\pm$3.5 y/o Control: (m=7), (f=13); Age: 22.4$\pm$3.2 y/o ACLD: N/A | To evaluate and compare the presence of abnormal knee and hip joint biomechanics during walking and jogging in groups of individuals at early, mid, and late time frames after unilateral ACLR surgery and a group of healthy controls. | vGRF | 1000 Hz | | 10 | 5-min warm-up periods before each task (no details)  Preferred jogging shoes, standardized speeds of 1.34 m/s and 2.68 m/s  Walking and jogging motion capture analysis of knee and hip kinetics and kinematics were measured in the sagittal and frontal planes on a split-belt instrumented treadmill |
| Luc-Harkey et al, 2018 [172] Experimental USA  In English | PA: Sports Level: Recreational Time Since ACLR: 47.8$\pm$27.0 months ACLR: (m=9), (f=21); Age: 20.4$\pm$2.9 y/o Control: N/A ACLD: N/A | To determine if peak vGRF, vGRF loading rate (vGRF-LR), the root mean square error (RMSE) between actual vGRF and target vGRF displayed via RTBF, and perceived difficulty of altering peak vGRF during walking differ when Real-Time Biofeedback Condition (RTBF) is provided to promote high-loading (increased vGRF), low-loading (decreased vGRF) and symmetrical loading in individuals with ACLR. | Peak Vertical Ground Reaction Force Instantaneous Vertical Ground Reaction Force Loading Rate Root Mean Square Error | 1000 | | 5 | Pre-determined self selected walking speed. Real-Time Biofeedback Conditions/Without  Once participants began walking on the treadmill, kinetic and kinematic outcomes were collected during five 60-second trials during each testing session, including Baseline,Acquisition1 (first intervention minute), Acquisition19 (final intervention minute), Recall1 (first post-intervention minute) and Recall45 (45-minutes post-intervention)  All participants were provided with a strategy that focused on manipulating the vertical displacement of their center of mass (CoM) to maximize the likelihood that participants would consistently reach the target. Specifically, participants were told that increasing or decreasing their vertical displacement of their CoM may result in a subsequent increase or decrease in peak vGRF. RTBF was not provided during the assessment of recall (Recall1, Recall45) and participants were instructed to “walk in the same manner as when attempting to match each vertical bar to the target line. |
| Luc-Harkey et al, 2018 [173] Cross Sectional USA  In English | PA: Sports Level: Recreational Time Since ACLR: 47.8$\pm$27.0 months ACLR: (m=9), (f=21); Age: 20.4 (2.9) y/o Control: N/A ACLD: N/A | To determine if peak vGRF and instantaneous vGRF loading rate on the ACLR limb and inter-limb asymmetry (limb symmetry index [LSI] = injured limb/uninjured limb) of these loading characteristics associate with the change in serum COMP concentration following a 20-minute bout of walking in individuals with ACLR. | ACLR limb peak vGRF (xBW) Contralateral limb vGRF (xBW) Peak vGRF limb symmetry index (%) ACL limb instantaneous vGRF loading rate (xBW/s)  Contralateral limb instantaneous vGRF loading rate (xBW/s)  Instantaneous vGRF loading rate limb symmetry index (%) | 1000 | | N/A | Pre-determined self-selected over-ground walking speed was used to set the speed of the instrumented treadmill for each participant. Participants then walked on the instrumented treadmill for 5 min to allow for acclimation and then rested quietly for 30 min prior to collection of the first blood sample (COMPpre). Following collection of the first blood sample, participants walked on the instrumented treadmill for 1 min to allow for collection of the peak vGRF and instantaneous vGRF loading rate, and continued walking at their self-selected speed for 20 min. A second blood sample was collected immediately following completion of the 20 min of walking (COMPpost). |
| Luc-Harkey et al, 2018 [171] Cross Sectional USA  In English | PA: Sports Level: Recreational Time Since ACLR: 47.8$\pm$27.0 months ACLR: (m=9), (f=21); Age: 20.4$\pm$2.9 y/o Control: N/A ACLD: N/A | To determine the associations between kinesiophobia and walking gait characteristics in physically active individuals with ACLR. Specifically, we separately determined the associations between kinesiophobia and 1) self-selected walking speed, 2) ACLR limb biomechanical outcomes (peak vGRF, instantaneous vGRF loading rate, peak KEM and knee flexion excursion, and 3) limb symmetry indices (LSI) of these biomechanical outcomes (peak vGRF LSI, instantaneous vGRF loading rate LSI, peak KEM LSI and knee flexion excursion LSI). | vGRF Instantaneous vGRF Loading Rate vGRF LSI | 1000 | | N/A | Participants walked on the treadmill at a pre-determined self selected walking speed for 5 min prior to collection of lower extremity biomechanical outcomes to allow for acclimation to treadmill walking. We collected kinematics and kinetics during a 60-s trial |
| Morgan et al, 2019 [174] Cross Sectional USA  In English | PA: N/R Level: N/R Time Since ACLR: 6.0 months (SD:N/R)  ACLR: (m=7), (f=8); Age: 21.2$\pm$8.4 y/o Control: (m=8), (f=7); Age: 21.0$\pm$4.4 y/o ACLD: N/A | To use Autoregressive (AR) modeling to delineate differences in dynamic stability between control and post-ACLR individuals as a result of peak vGRF data during running. vGRF data were analyzed during running because running is a more demanding task than walking, thus differences in motor control and dynamic stability would become more apparent | Peak vGRF (in relation to body weight) | 1200 Hz | | 1 | Shoes on.  Participants ran at a self-selected speed to get acclimated to the instrumented split-belt treadmill. Once acclimated, participants were instructed to jog at a comfortable pace. |
| **Studies used Pressure Mats to assess "Gait" (n=1)** | | | | | | | |
| Armitano-Lago et al, 2020 [175] Cross Sectional USA  In English | PA: N/R Level: N/R Time Since ACLR: 8.9$\pm$6.0 months ACLR: (m=8), (f=8); Age: 29.2$\pm$6.9 y/o Control: (m=8), (f=8); Age: 28.9$\pm$6.2 y/o ACLD: N/A | 1- To assess whether individuals with a history of ACLR exhibit altered neuro-motor function when compared to healthy controls.  2- To examine spatiotemporal, balance, ankle dorsiflexion ROM, proprioception, joint laxity, patellar tendon reflex latency, and quadriceps strength measures to provide a robust picture of the participants overall neuro-motor function and to help identify the locus of differences between the ACLR and control individuals | Spatiotemporal gait measures (i.e., velocity, cadence, step length and width) | 150 Hz | | 3 | Preferred walking speed, and fast walking speed  Participants walked a total of 28 ft. with a 20 ft. walking surface positioned in the center of the path. |
| *N/R* Not reported, *N/A* Not applicable, *PA* Physical activity, *Level* Activity level, *SD* standard deviation, *IQR* Interquartile range, *ACLR* Anterior cruciate ligament reconstruction, *ACLD* anterior cruciate ligament deficient, *GRF* Ground reaction force, *vGRF* vertical ground reaction force, *xBW* Normalized to body weight, *CoG* center of gravity, *LSI* limb symmetry index, *SI* Symmetry index. | | | | | | | |

Table 4 Data Extraction Table for Studies Assessing Cutting Movements/Change of Directions

| **Study Characteristic**  **(author, year, design, country, language)** | **Sample Characteristic**  **(Physical Activity (PA), level, time since surgery, side of surgery, sample size by sex, age)** | **Study Objectives** | **Parameters** | | **Sampling Frequency** | **Number of Repetitions** | - **Testing Condition or Challenges** - **Protocol Summery** |
| --- | --- | --- | --- | --- | --- | --- | --- |
| **Studies used Force Plates to assess "Cutting Movement/Change in Direction" (n=8)** | | | | | | | |
| Bjornaraa et al, 2011 [177] Cross Sectional USA  In English | PA: N/R Level: N/R Time Since ACLR: 55.2$\pm$32.4 months ACLR: (m=0), (f=17); Age: 26.5$\pm$6.3 y/o Control: (m=0), (f=17); Age: 25.3$\pm$6.0 y/o ACLD: N/A | 1) Determine if subjects with ACL reconstruction display different knee displacements, velocities, and time to peak GRF during cutting activities than healthy subjects 2) Observe if subjects with visual disruption display differences in these variables than with vision available 3) Determine if visual deprivation alters these same variables in subjects with ACL reconstruction more significantly than in healthy subjects. Additionally, limb to limb comparisons will be completed within the ACLR group and healthy group to determine whether asymmetries exist between surgical and non-surgical or dominant and non-dominant extremities, respectively | Time to peak GRF during cutting activities | 1000 Hz | | 10 | Shoes on, full vision/disturbed vision  Cutting movement during which knee position was measured via a 3D electromagnetic system. Visual conditions were randomized to disrupt vision for 1 second as the subject began the cutting movement, or allow full vision for movement duration. Independent variables were lead/push off leg (ACLR limb or healthy non-dominant limb) and vision (disrupted or full) |
| Chang et al, 2018 [28] Quasi Experimental South Korea  In English | PA: N/R Level: N/R Time Since ACLR: ACLR: 35.2$\pm$13.2 months ACLR: (m=0), (f=18); Age: ACLR: 19.9$\pm1.2$ Control: (m=0), (f=12); Age: 21.0$\pm$2.6 y/o ACLD: N/A | To compare the landing biomechanics of ACLR females who pass or fail an FTB to the matched-limb landing biomechanics of healthy females before and after completion of a sustained exercise protocol | Peak vGRF | 1560 Hz | | 3 | 5-min submaximal warm-up on a stationary bike  For double leg jump landing, participants stood atop a 30-cm high box placed 50% of their height from the front edge of the force plate. They were instructed to jump forward off the box and land on the force plates with both feet then immediately jump vertically. For single leg jump landing, participants stood on the floor behind a line marked 50% of their height from the front edge of the force plate. Then, they were instructed to jump over a 17-cm high hurdle placed 25% of their height from the force plate, land on 1 foot (testing foot), and then cut as quickly as possible to the other direction of the landing foot (e.g. right foot landing then cut to the left side). |
| Chang et al, 2020 [29] Cross Sectional South Korea  In English | PA: N/R Level: N/R Time Since ACLR: 35.2$\pm$18.4 months ACLR: (m=0), (f=18); Age: 19.9$\pm$1.2 y/o Control: (m=0), (f=12); Age: 21.0$\pm$2.6 y/o ACLD: N/A | To compare the knee joint landing and cutting biomechanics asymmetry of ACLR females that pass and fail an FTB with healthy females before and after the completion of a sustained exercise protocol. It was hypothesized that there would be no differences in landing and cutting mechanics asymmetry between ACLR females that pass an FTB (ACLR-pass) and healthy females; but that ACLR females that fail an FTB (ACLR-fail) would exhibit different landing and cutting mechanics asymmetry compared to ACLR-pass and healthy females. | Peak vGRF vGRF loading rate | 1560 Hz | | 3 | Participants warmed-up for 5-min on a stationary bike at self-selected speed  For double leg jump landing, participants stood atop a 30-cm high box placed 50% of their height from the front edge of the force plate. They were instructed to jump forward off the box and land on the force plates with both feet then immediately jump vertically. For single leg jump landing, participants stood on the floor behind a line marked 50% of their height from the front edge of the force plate. Then, they were instructed to jump over a 17-cm high hurdle placed 25% of their height from the force plate, land on 1 foot (testing foot), and then cut as quickly as possible to the other direction of the landing foot (e.g. right foot landing then cut to the left side). |
| King et al, 2018 [176] Cross Sectional  Ireland  In English | PA: Multidirectional field sports (i.e. Gaelic Football, Soccer, Hurling, Rugby). Level: N/R Time Since ACLR: 8.8$\pm$0.7 months ACLR: (m=156), (f=0); Age: 24.8$\pm$4.8 y/o Control: N/A ACLD: N/A | The first aim of this study was to identify differences in timed performance and biomechanical variables through the kinetic chain between the ACLR and non-ACLR limbs during stance phase of a 90° planned and unplanned CoD. The second aim was to identify differences in kinematic and kinetic variables, between planned and unplanned CoD for each leg | GRF | 1000Hz | | 2 submaximal trials and 3 maximal trials | A standardized warm-up including 2-min jog, 5 bodyweight squats, 2 submaximal, and 3 maximal countermovement jumps  Shoes on.  Participants carried out 90 degrees maximal effort, planned and unplanned CoD tests in a 3D motion capture laboratory 9 months after ACLR. Statistical parametric mapping (2 x 2 ANOVA; limb x test) was used to identify differences in CoD time and biomechanical measures between limbs and between tests |
| King et al, 2019 [85] Cross Sectional  Ireland  In English | PA: Multidirectional field sports Level: N/R Time Since ACLR: 9.4$\pm$0.7 months ACLR: (m=156), (f=0); Age: 24.8$\pm$4.2 y/o Control: (m=62), (f=0); Age: 24.7$\pm$3.9 y/o ACLD: N/A | To identify differences in asymmetry of biomechanical and performance variables during jump and CoD testing between athletes who were 9 months post-ACLR and a matched healthy cohort | Ground reaction force in vertical, medial and posterior directions | 1000 Hz | | 3 | A standardized warm-up including 2-min jog, 5 bodyweight squats, 2 submaximal, and 3 maximal countermovement jumps  Shoes on  The testing protocol included the double-legged drop jump from 30 cm, the single-legged drop jump from 20 cm, the single-legged hop for distance, and 90° planned and unplanned change of direction |
| Lanier et al, 2020 [178] Cross Sectional USA  In English | PA: Level I and II sports Level: N/R Time Since ACLR: 8.0$\pm$1.8 months ACLR: (m=3), (f=8); Age: 21.0$\pm$7.8 y/o Control: (m=11), (f=16); Age: 21.0$\pm$1.1 y/o ACLD: (m=7), (f=3); Age: 24$\pm$8.2 y/o | To determine how ACL injury, ACL reconstruction, and participation in high‐performance athletics affects control strategies produced during a novel task that simulates forces produced during cutting movements | Lyapunov exponent (LyE) (high LyE suggests greater variability and poorer motor control) | N/R | | 3 | Participants generate force in a back and forth manner, continuously, and to the beat of a metronome set at 60 beats per minute. Participants received real‐time visual feedback of their AP or ML force production |
| Lim et al, 2015 [168] Cohort South Korea  In English | PA: N/R Level: N/R Time Since ACLR: 6.0 months (SD:N/R) ACLR: (m=N/R), (f=); Age: 31.6$\pm$7.0 y/o Control: (m=N/R), (f=); Age: 33.4$\pm$6.0 y/o ACLD: N/A | To compare the early functional recovery using biomechanical properties between ACL- and PCL-reconstructed patients and to determine the biomechanical deficit of PCL-reconstructed patients compared to ACL-reconstructed patients | Ground reaction force | 1200 Hz | | 3 | 5-min warm-up prior to testing  Participants were required to perform a cutting 45°, 90°, 135°, and 180° turn walking, and 180° turn running task along the laboratory gateway. |
| Miranda et al, 2013 [54] Cross Sectional USA  In English | PA: N/R Level: Recreational Time Since ACLR: 60.0 months (SD:N/R) ACLR: (m=4), (f=6); Age: 26.96$\pm5.3$ y/o Control: (m=5), (f=5); Age: 25.20$\pm5.2$y/o ACLD: N/A | To compare force plate kinetic data and knee kinematic measurements from male and female ACLINT and ACLREC recreational athletes during a jump-cut maneuver in hopes that the differences would point to plausible risk factors for injury | Peak GRF (magnitude of body weight), peak vertical GRF time, peak vertical GRF magnitude | 5000 Hz | | 10 | Subjects stood 1 m from force plate with knees bent approx. 45 degrees. Upon hearing "go" prompt, subject jumped forward to landing target on force plate, and a visual directional prompt cued subject to cut left or right after landing on the target with 1 leg. Upon landing, subjects performed a side step cut and then jogged past the respective angled targets. |
| *N/R* Not reported, *N/A* Not applicable, *PA* Physical activity, *Level* Activity level, *SD* standard deviation, *ACLR* Anterior cruciate ligament reconstruction, *ACLD* anterior cruciate ligament deficient, *GRF* Ground reaction force, *vGRF* vertical ground reaction force. | | | | | | | |

Table 5 Data Extraction Table for Studies Assessing Squatting

| **Study Characteristic**  **(author, year, design, country, language)** | **Sample Characteristic**  **(Physical Activity (PA), level, time since surgery, side of surgery, sample size by sex, age)** | **Study Objectives** | **Parameters** | | **Sampling Frequency** | **Number of Repetitions** | - **Testing Condition or Challenges** - **Protocol Summery** |
| --- | --- | --- | --- | --- | --- | --- | --- |
| **Studies used Force Plates to assess "Squatting" (n=5)** | | | | | | | |
| Rudroff et al, 2003 [65] Cross Sectional Germany  In English | PA: Soccer Level: N/R Time Since ACLR: 24.0 months (SD:N/R) ACLR: (m=30), (f=0); Age: 30.9$\pm$5.4 y/o Control: (m=10), (f=0); Age: 31.1$\pm$4.7 y/o ACLD: N/A | To compare the clinical outcome of ACL reconstruction using the four-strand ham- string tendon autografts and ACL reconstruction using the patellar tendon graft 2 yr. after surgery. | Vertical jump-off force, first vertical maximum | 1000 Hz | | Squats: 5 trials; gait, one- and two-legged jumps: 6 trials | Squats: in standing position, femurs rotated externally (feet abducted 20 degrees), lowered center of mass to 90 degrees at approx. 30 deg/sec |
| Salem et al, 2003 [179] Cross Sectional USA  In English | PA: N/R Level: N/R Time Since ACLR: 7.5$\pm$3.0 months ACLR: (m=7), (f=1); Age: 27.9$\pm$6.8 y/o Control: N/A ACLD: N/A | To characterize the bilateral lower-extremity kinematics and kinetics associated with squatting exercise after anterior cruciate ligament (ACL) reconstruction. | Peak vertical ground reaction force | 600 Hz | | 3 | 5-min warm-up on a stationary bike  Standing with 1 foot on each of 2 force platforms, were instructed to assume a stance width consistent with that used during their previous rehabilitation training. They were then instructed to “descend (at self-selected movement pace) to a level to where your posterior thighs are parallel to the floor and then ascend.” Participants performed 3 sets of 10 repetitions of the back squat exercise using a resistance weight of 35% body weight. |
| Sanford et al, 2016 [180] Cross Sectional USA  In English | PA: Sport Level: N/R Time Since ACLR: 86.4$\pm75$.6 months ACLR: (m=3), (f=5); Age: 28.0$\pm$7.0 y/o Control: (m=3), (f=5); Age: 25.0$\pm$4.0 y/o ACLD: N/A | To test whether ACL reconstructed subjects have symmetric three-dimensional ground reaction forces as assessed using PCA and symmetric AP translation rates of the femur with respect to the tibia when compared with healthy control subjects. | AP GRF ML GRF vGRF | 1000 Hz | | 2 | Barefoot. Feet shoulder width apart.  Keep the torso as upright as possible and to perform continuous bilateral squats to a comfortable level of knee flexion with arms held straight out in front of the chest.  Participants were asked to squat at a self-selected pace for an interval of 25 s for 2 trials of data collection. |
| Schilling et al, 2020 [66] Cohort USA  In English | PA: Sports Level: Collegiate Athlete Time Since ACLR: Range (6-71) months ACLR: (m=7), (f=14); Age: 20.3$\pm$1.7 y/o Control: N/A ACLD: N/A | To assess the readiness for return to sport in a sample of division III athletes following ACLR and medical clearance. | vGRF | N/R | | 3 | Participants performed a single maximum single-leg squat test (SLST) while standing on a 12 in.-high step (30.48 cm).   The participants were then asked to perform a single-leg landing task from the step and land on a force plate that was located 12 inches away from the step. |
| Webster et al, 2015 [181] Cross Sectional Australia  In English | PA: Sports Level: N/R Time Since ACLR: 17.3$\pm$2.3 months ACLR: (m=10), (f=0); Age: 23.0$\pm$3.0 y/o Control: (m=10), (f=0); Age: 32.0$\pm$2.0 y/o ACLD: N/A | To analyze weight-bearing symmetry along with hip and knee joint symmetry during a double leg squat in a group who had undergone ACLR surgery, both at baseline and following fatigue, and compare symmetry to an uninjured control group. | vGRF Weight bearing symmetry | N/R | | 3 | During testing, participants were asked to stand with 1 foot on each force plate with a comfortable stance width. Ten consecutive double limb squats were then performed at a steady pace (4 s/squat; 2 s descent, 2 s ascent). Participants were instructed to descend to a level where the thighs were parallel to the floor, whilst keeping the arms parallel to the ground and then ascend to a full upright position. |
| **Studies used Pressure Mats to assess "Squatting" (n=1)** | | | | | | | |
| Dan et al, 2019 [89] Cross Sectional Australia  In English | PA: N/R Level: N/R Time Since ACLR: Range (8-15) months ACLR: (m=47), (f=18); Age: 33.8$\pm$10.1 y/o Control: (m=17), (f=20); Age: 25.1$\pm$8.6 | To explore the utility of this accelerometer and gyroscope system as well as a pressure sensing mat in detecting kinetic differences in patients prior to return to sport following ACL reconstruction. | Peak Load | N/R | | N/R | Barefoot Single and double leg |
| *N/R* Not reported, *N/A* Not applicable, *PA* Physical activity, *Level* Activity level, *SD* standard deviation, *ACLR* Anterior cruciate ligament reconstruction, *ACLD* anterior cruciate ligament deficient, *GRF* Ground reaction force, *vGRF* vertical ground reaction force, *AP* Anterior Posterior, *ML* Medial Lateral. | | | | | | | |

Table 6 Data Extraction Table for Studies Assessing Stop Jump, Step-Over & Lunges

| **Study Characteristic**  **(author, year, design, country, language)** | **Sample Characteristic**  **(Physical Activity (PA), level, time since surgery, side of surgery, sample size by sex, age)** | **Study Objectives** | **Parameters** | | **Sampling Frequency** | **Number of Repetitions** | - **Testing Condition or Challenges** - **Protocol Summery** |
| --- | --- | --- | --- | --- | --- | --- | --- |
| **Studies used Force Plates to assess "Stop Jump" (n=2)** | | | | | | | |
| Queen et al, 2020 [182] Cross Sectional USA  In English | PA: N/R Level: N/R Time Since ACLR: 6.6$\pm$1.6 months ACLR: (m=13), (f=9); Age: 17.3$\pm$2.2 y/o Control: (m=13), (f=9); Age: 22.3$\pm$2.7 y/o ACLD: N/A | To test the performance of these indices on previously unpublished data on ACL-R patients and to propose a new index to resolve some of these limitations. | Indices for peak vGRF (ratio index, gait asymmetry index, symmetry index, symmetry angle, normalized symmetry index) | 2400 Hz | | 5 | Bilateral  Participants were told to approach as quickly as possible and to jump as high as they felt was safely possible, and no instructions about landing position or technique were provided. |
| Renner et al, 2018 [183] Cohort USA  In English | PA: N/R Level: Recreational or high school sport Time Since ACLR: 6.0 months (SD:N/R)  ACLR: (m=9), (f=14); Age: 16.0$\pm$1.3 y/o Control: N/A ACLD: N/A | To examine differences in movement and loading patterns across time and between limbs in ACLR patients over 4 visits in the first year post-ACLR. | Peak vertical GRF, Peak posterior GRF, loading rate, impulse | 2400 Hz | | 5 | Jump task includes several running steps, a jump off 1 foot, a two-footed landing, and a subsequent two-footed jump. Participants were told to approach the force plate as quickly as possible and to jump as high as was safely possible. No instruction on landing was given |
| **Studies used Force Plates to assess "Step-Over & Lunges" (n=1)** | | | | | | | |
| Mattacola et al, 2004 [184] Cross Sectional USA  In English | PA: N/R Level: N/R Time Since ACLR: 14.5$\pm$4.8 months Dominant/Non-dominant: N/R ACLR: (m=13), (f=5); Age: 22.8$\pm$5.8 y/o Control: (m=10), (f=8); Age: 22.5$\pm$4.1 y/o ACLD: N/A | To evaluate the performance of an ACL reconstruction group and a control group during 2 functional tests. | The lift-up index Movement time The impact index  Lunge distance, Contact time, Impact index, and Force impulse. | N/R | | 3 | 5-min warm-up on the treadmill.  To perform the step-up-and-over test, each individual started by standing with both feet stationary on the force plate. They then stepped with 1 leg (lead leg) up onto a box 12-in tall, which was also on the force plate. The lagging leg was carried up and over the box, landing on the surface opposite from the original starting position. The individual started in a standing position, lunged forward with 1 leg, and then returned to the original standing position |
| *N/R* Not reported, *N/A* Not applicable, *PA* Physical activity, *Level* Activity level, *SD* standard deviation, *ACLR* Anterior cruciate ligament reconstruction, *ACLD* anterior cruciate ligament deficient, *GRF* Ground reaction force, *vGRF* vertical ground reaction force. | | | | | | | |
